# Polycondensation as a Universal Method for Preparing High‐Density Single‐Atom Catalyst Libraries

**DOI:** 10.1002/adma.202507627

**Published:** 2025-07-04

**Authors:** Jaques‐Christopher Schmidt, Jan Romano‐deGea, Dragos C. Stoian, Mounir Mensi, Miyeon Chang, Ariana Serban, Satyadeep Waiba, Xinbang Wu, Lindsey E. K. Frederiksen, Rosie J. Somerville, Roland C. Turnell‐Ritson, Xunhui Wang, Laura Piveteau, Daniel Ortiz, Niccolò Martinolli, David Reyes, Jordi Espín, Timo M. O. Felder, Stefano Di Leone, Pascal Miéville, Shoubhik Das, Paul J. Dyson

**Affiliations:** ^1^ Institute of Chemical Sciences and Engineering École Polytechnique Fédérale de Lausanne (EPFL) Lausanne 1015 Switzerland; ^2^ The Swiss‐Norwegian Beamlines (SNBL) European Synchrotron Radiation Facility (ESRF) Grenoble 38000 France; ^3^ Department of Chemistry University of Bayreuth 95447 Bayreuth Germany; ^4^ Department of Chemistry Chemistry Research Laboratory University of Oxford 12 Mansfield Road Oxford OX1 3TA UK; ^5^ Nanoelectronic Devices Laboratory (Nanolab) Institute of Electrical and Micro Engineering École Polytechnique Fédérale de Lausanne (EPFL) Lausanne 1015 Switzerland; ^6^ Interdisciplinary Center for Electron Microscopy École Polytechnique Fédérale de Lausanne (EPFL) Lausanne 1015 Switzerland; ^7^ Swiss Cat+ West Hub École Polytechnique Fédérale de Lausanne (EPFL) Lausanne 1015 Switzerland

**Keywords:** automation, coordination polymers, electrocatalysis, photocatalysis, single atom catalysts, universal method

## Abstract

Single‐atom catalysts (SACs) present a promising subclass of classic heterogeneous catalysts by maximizing metal dispersion and enhancing efficiency. Although high‐density SACs (HD‐SACs) are reported, their synthesis is typically constrained to specific metal‐support combinations and high‐temperature annealing, limiting their translation to wider applications. Herein, a universal bottom‐up approach for the preparation of mono‐ and bimetallic HD‐SACs based on the polycondensation of 1,2,4,5‐benzenetetramine with a wide range of metal monomers containing 1,10‐phenanthroline‐5,6‐dione ligands is introduced. The synthesized materials are atomically dispersed and exhibit metal loadings up to 27.5 wt% with high structural stability. Their versatility as catalysts is explored in electrocatalytic and photocatalytic applications. The materials exhibit remarkable stability under operational conditions. Furthermore, this synthetic strategy is scaled up and automated, demonstrating the robustness and reproducibility and laying the groundwork for self‐optimizing data‐driven materials discovery.

## Graphical abstract

This paper presents a universal bottom‐up polycondensation strategy for synthesizing high‐density single‐atom catalysts (HD‐SACs) with metal loadings up to 27.5 wt%. Using various metal monomers with 1,10‐phenanthroline‐5,6‐dione ligands, it generates atomically dispersed mono‐ and bimetallic HD‐SACs. The materials demonstrate high stability in electrocatalytic and photocatalytic applications, and the method is compatible with automation and scale‐up.

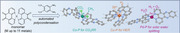

## 1 Introduction

Supported metal‐based catalysts are the state‐of‐the‐art in many reactions due to their high activity, stability, and recyclability.^[^ ^1^, ^2^, ^3^, ^4^, ^5^, ^6^ ^]^ These catalysts often contain metal nanoparticles, but typically only surface or interfacial atoms are involved in the catalytic activity, with much of the catalytically active metal not being utilized.^[^ ^7^, ^8^, ^9^ ^]^ Improving the efficiency of catalytic materials with respect to metal loading, by maximizing the dispersion of precious metals, has become a key target for the design of new catalysts.^[^ ^10^, ^11^, ^12^, ^13^, ^14^ ^]^ Heterogeneous single‐atom catalysts (SACs) consist of metals atomically dispersed on solid supports and have the potential to push boundaries of activity, as well as selectivity due to their monodisperse nature.^[^ ^15^, ^16^, ^17^, ^18^, ^19^, ^20^, ^21^, ^22^ ^]^ High metal loadings are desirable for implementing SACs in large‐scale technical applications, as they increase productivity per unit reactor area or volume. Although many methods have been published for producing SACs with metal loadings up to 2 wt%,^[^ ^23^, ^24^ ^]^ most reported cases of high‐density SACs (HD‐SACs), defined herein as having metal contents over 5 wt%, are typically limited to specific metal/support combinations and cannot be readily generalized.^[^ ^14^ ^]^ Methods for the preparation of HD‐SACs applicable to a wide range of transition metals are rare, and limited to finely tuned annealing methods (up to 23^[^ ^25^ ^]^ and 44.8 wt%^[^ ^26^ ^]^), modified graphene quantum dots as support material (up to 40 wt%), ^[^ ^27^ ^]^ atomic trapping (up to 33.4 wt%)^[^ ^28^ ^]^ and 3D‐printing approaches (up to 20.8 wt%).^[^ ^29^ ^]^ This limitation arises as most methods involve a heating step at elevated temperatures (>300 °C), restricting the number of suitable metals for each support, as the metal‐support interactions need to be sufficiently strong to prevent aggregation.^[^ ^15^ ^]^ Furthermore, the majority of the reported procedures involve the use of supports with pre‐introduced coordination sites without well‐defined structures, thereby reducing the tunability of the coordination environment. Additionally, the production of heterometallic SACs is barely addressed in the literature, although they could result in important new processes.

Stabilizing metal sites in a porous organic polymer (POP) is a promising approach to producing SACs without the need for high‐temperature annealing, thereby reducing the risk of aggregation of metal species into clusters or nanoparticles.^[^ ^30^ ^]^ Atomically dispersed metal sites integrated into amorphous coordination polymers (ACP), a subclass of POP‐SACs, further favor coordination site fine‐tuning due to the wide variety of ligands that can be used in the polymerization process, making them a useful tool for producing highly stable catalysts with defined structures.^[^ ^31^, ^32^ ^]^ The reported synthetic approaches lack proof of translatability to other elements, highlighting the need for robust and general methods.

**Figure 1:**
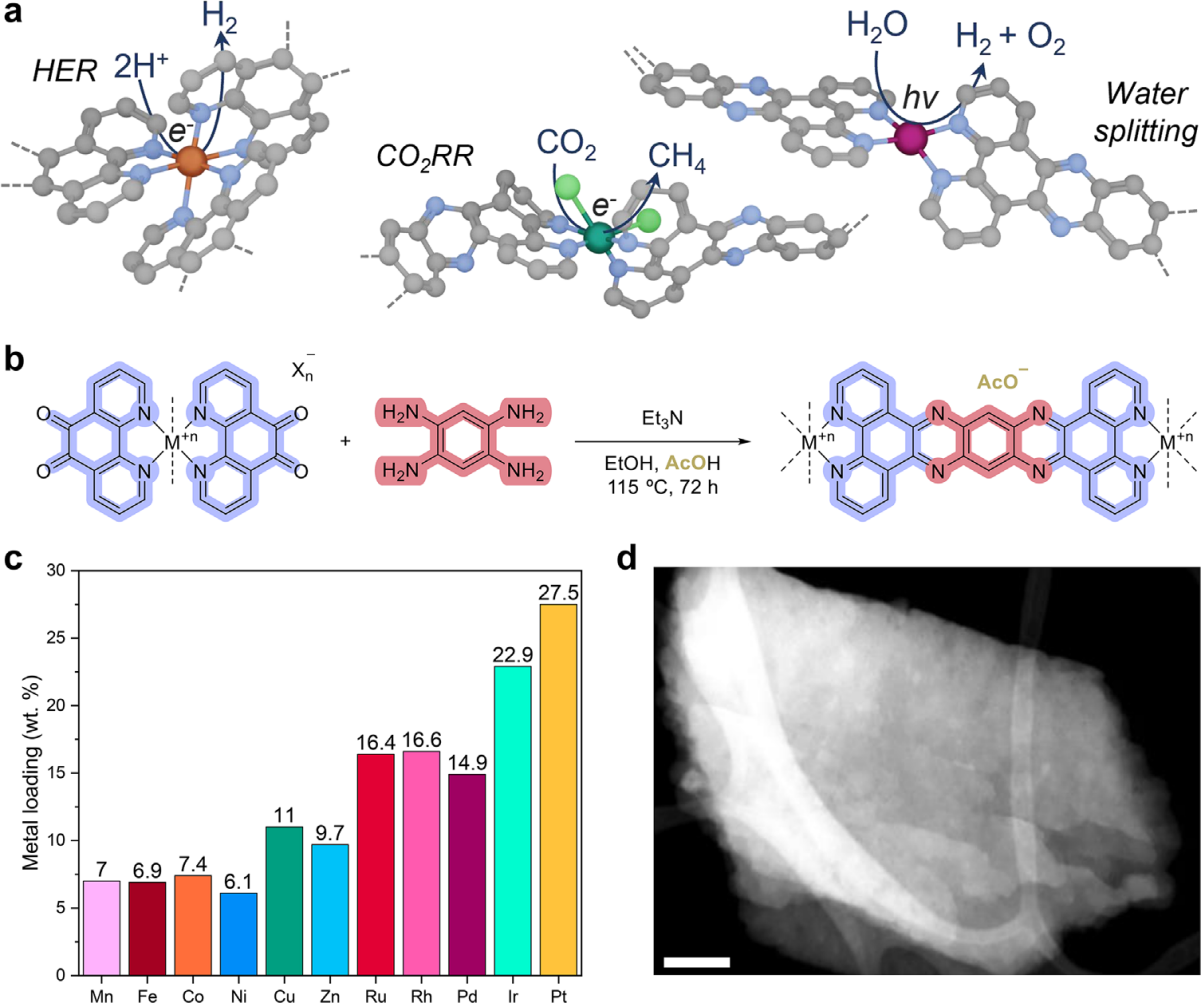
Synthesis of HD‐SACs. a) Representative use cases of the HD‐SACs in this paper. b) Polycondensation reaction between the corresponding **Metal‐M** and 1,2,4,5‐benzenetetramine to give **tatppb**‐supported HD‐SACs. Dashed lines indicate another **tatppb** unit or other ligands. **Metal‐M** containing chloride ligands or counterions undergo substitution by acetates. c) Metal loadings of the monometallic HD‐SACs. **d**, HAADF‐STEM image of **Rh‐P**. Scale bar 100 nm.

Herein, we report a universal, bottom‐up approach for the preparation of mono‐ and bimetallic ACP self‐supported HD‐SACs. The approach comprises preparing metal monomers containing two or three 1,10‐phenantholine‐5,6‐dione ligands, which subsequently undergo polycondensation with 1,2,4,5‐benzenetetramine to afford coordination polymers of interconnecting ligands with a defined structure and nitrogen‐coordinating functionalities that anchor the metals in well‐defined topologies. We have applied this versatile method to eleven metals, but it is potentially universally applicable to any metal able to coordinate to at least two 1,10‐phenanthroline ligands. A review of the literature, including the crystallographic CCDC database, identifies over 51 metals and metalloids with at least one compound that meets the required structure (more details in Figure S1, Supporting Information). For the reported HD‐SACs, the atomic dispersion of the metals was confirmed, and the chemical structure and morphology of the materials were determined. The synthesized materials have been explored as electrocatalysts and photocatalysts in synthetic fuel generation (**Figure** **1**a), with the HD‐SACs displaying high stability under the reaction conditions. Furthermore, a scale‐up to gram‐scale production further confirms the robustness and scalability of the approach. The synthesis was also automated, demonstrating the reproducibility of the approach and establishing the foundations for the automated optimization and discovery of new heterometallic SAC materials.

## 2 Results and Discussion

### 2.1 Polycondensation Approach

The polycondensation approach introduced here was used to generate mono‐ and bimetallic HD‐SACs from eleven metals (Figure S1, Supporting Information in green), but is potentially universally applicable to the entire transition metal series, and in principle to any other metals that can coordinate two 1,10‐phenanthroline ligands (see Figure S1, Supporting Information for more detailed information). The approach relies on the production of metal complexes that are bound to at least two 1,10‐phenanthroline‐5,6‐dione ligands, which then undergo polycondensation with 1,2,4,5‐benzenetetramine to form pyrazine groups that link the metal centers together in a polymeric structure (Figure 1b). The strength of the proposed method lies in achieving homogeneously dispersed metal loadings by pre‐anchoring the metal atoms within the well‐defined nitrogen binding sites of the 1,10‐phenanthroline‐5,6‐dione ligand, which leads to stable atomically dispersed materials after polycondensation. Furthermore, the reaction conditions are relatively mild (115 °C), avoiding the sintering of the metal atoms into clusters or nanoparticles during the reaction. Our approach provides a platform for the production of ACP‐POPs, which could be fine‐tuned depending on the scientific objective. In comparison to other approaches for HD‐SACs libraries using supports like nitrogen‐doped carbon or metal oxides, the modifications of the developed SACs are more easily achieved. For example, exchanging the 1,2,4,5‐benzeneteramine for a naphthalene‐ and anthracene analogue would increase the π‐conjugation of the material with ramifications in photocatalysis, or introducing specific substituents in the 1,10‐phenanthroline‐5,6‐dione ligand would tune the sterics around the metal or modulate the affinity for certain substrates, further improving the material performance. However, it is worth noting that these advantages come with a higher material cost in comparison to using commonly accessible supports. The procedure involves suspending in a closed vessel the metal monomer(s) (labeled as **Metal‐M**, e.g., **Ru‐M**, see Supporting Information for synthesis and characterization) and the connecting unit, i.e., 1,2,4,5‐benzenetetramine, in a mixture of ethanol and acetic acid (Figure 1b). The polycondensation reaction is initiated by the addition of triethylamine to deprotonate the 1,2,4,5‐benzenetetrammonium hydrochloride salt, and then the mixture is heated under reflux for 72 h to ensure full conversion. The reaction may be monitored by Fourier‐transformed infrared (FTIR) spectroscopy, with the disappearance of the carbonyl stretch in the metal monomer (ca. 1700 cm^−1^) indicating completion of the reaction (see FTIR discussion in Section 2.2). After polymerization, insoluble dark powders (labelled as **Metal‐P**, e.g., **Ru‐P**) are obtained. Compared to top‐down approaches and other methods for the preparation of atomically dispersed materials, this bottom‐up approach enables control over the chemical structure of the materials and coordination environment of the metals. The obtained materials are composed of metals connected by planar‐conjugated bisphenanthroline units (9,11,20,22‐tetraazatetrapyridopentacene, **tatppb**). Depending on the geometry of the metal monomer, three different polymer architectures can be expected (Figure S2, Supporting Information). Four‐coordinate square planar metal monomers with two 1,10‐phenanthroline‐5,6‐dione ligands result in distorted square‐planar linear structures, i.e., **Pd‐P** and **Pt‐P**. In contrast, six‐coordinate octahedral complexes with two 1,10‐phenanthroline‐5,6‐dione ligands and two chloride ligands lead to bent linear structures, i.e., **Mn‐P**, **Cu‐P**, **Zn‐P**, **Ru‐P**, **Rh‐P,** and **Ir‐P**. Six‐coordinate octahedral complexes with three 1,10‐phenanthroline‐5,6‐dione ligands yield structures expanding in three axes directions, i.e., **Fe‐P**, **Co‐P,** and **Ni‐P**. Furthermore, the method was extended to the synthesis of bimetallic systems, i.e., **CoCu‐P** and **CoRu‐P**, demonstrating the scope of the method to synthesize a tunable homogenously distributed material library with controlled metal ratios.

Analysis of the obtained HD‐SACs by inductively coupled plasma mass spectrometry (ICP‐MS) shows that the polycondensation method leads to materials with metal loadings between 6.1 and 27.5 wt.% (Figure 1c), as expected from the different metal‐to‐ligand ratios and the intrinsic molecular weights of the different metal monomer employed. Scanning electron microscopy (SEM) revealed the formation of amorphous materials composed of microstructures a few hundred nanometers wide (Figure S3, Supporting Information) that in the high‐angle annular dark‐field scanning transmission electron microscopy (HAADF‐STEM) images appear to be plate‐like material (Figure 1d; Figure S4, Supporting Information). Powder X‐ray diffraction (pXRD) patterns confirm the absence of crystalline structures or nanoparticles in all the prepared HD‐SACs (Figure S6, Supporting Information), rather highlighting their amorphous character, with the exception of **Ru‐P** and **Rh‐P**. For these, the pXRD pattern indicates the formation of an ordered crystalline polymer structure that are not identical to **Ru‐M** and **Rh‐M** and that could not be identified or isolated.

Brunauer‐Emmett‐Teller (BET) surface areas of the HD‐SACs determined from N_2_ adsorption‐desorption isotherms at 77 K ranged from 13 to 63 m^2^ g^−1^ and showed no significant hysteresis or micro‐porosity (Figure S7a, Supporting Information). The areal density of metal atoms per nm^2^ was calculated based on the BET results of the HD‐SACs (Figure S7b, Supporting Information). The electrical conductivity of the materials was measured using a standard three‐electrode procedure, revealing conductivities between 3.6 × 10^−10^ and 8.2 × 10^−11^ S cm^−1^, comparable to some undoped conductive polymers (see Supporting Information).^[^ ^33^, ^34^ ^]^ Thermogravimetric analysis (TGA) measurements coupled with differential scanning calorimetry (DSC) and mass spectrometry (MS) were used to determine the thermal stability of the HD‐SACs under an inert atmosphere. A steady decrease in mass is observed in the TGA spectra of the HD‐SACs (Figure S8, Supporting Information). Residual water or solvent is released below 200 °C, at which point there is an initial release of CO_2_ and CH_4_, presumably from the decarboxylation of acetate (arising from anion exchange during synthesis, Figure 1a). No nitrogen species (in the 16–44 m/z range) are observed until 600 °C, when ammonia is detected, indicating that until this temperature, the coordination environment of active sites likely remains stable. The HD‐SACs progressively lose mass without any distinctive DSC features up to 900 °C, retaining ≈65% of the original mass in all cases. **Ru‐P** and **Co‐P** annealed under inert atmosphere at 300 and 500 °C, respectively, do not show any metal aggregation when examined using HAADF‐STEM, further confirming the high stability of the coordination environment around the metal sites (Figure S9, Supporting Information).

### 2.2 Atomic State and Chemical Structure

**Figure 2:**
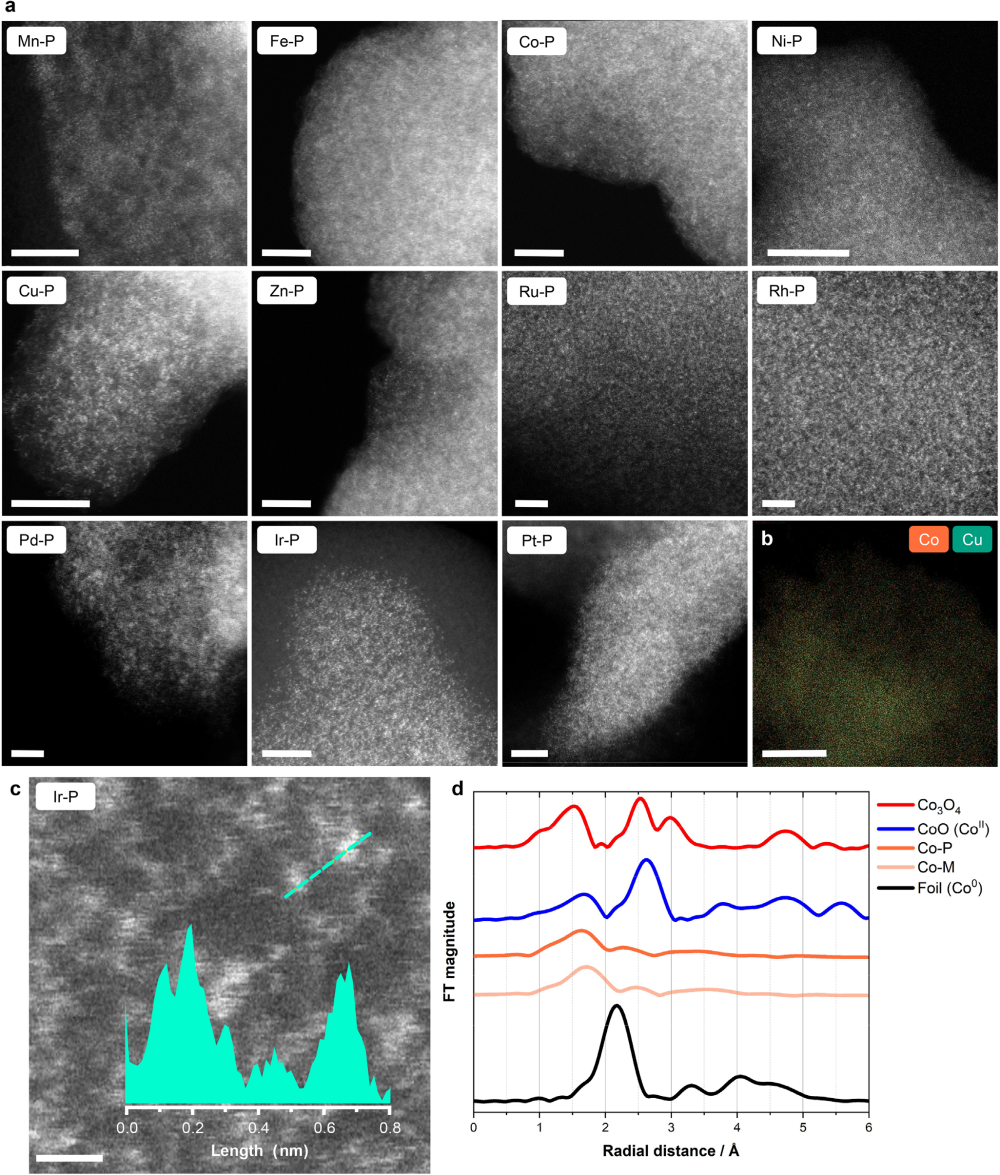
Electron microscopy and spectroscopic characterization of the HD‐SACs. a) Atomic‐resolution HAADF‐STEM images of the HD‐SACs. Scale bars 5 nm. b) EDX map of Co and Cu in **CoCu‐P**. Scale bar 100 nm. c) Enlarged area of the HAADF‐STEM image of **Ir‐P**. Scale bar 0.5 nm. The graph shows the intensity profile along the dashed line. d) Fourier transformation (not corrected for phase shift) of the Co L‐edge EXAFS spectra of **Co‐M**, **Co‐P**, and references.

HAADF‐STEM images of the HD‐SACs are shown in **Figure** **2**a. The high density of bright features observed is characteristic of isolated metal atoms. Elemental mapping by energy‐dispersive X‐ray (EDX) spectroscopy of the HD‐SACs confirms the homogeneous metal distribution and the absence of aggregates (Figure S4, Supporting Information). The bright dots in the HAADF‐STEM image of **Ir‐P** have diameters of around 0.2 nm, which is comparable to the ionic diameter of Ir^III^ (0.16 nm), indicative of the presence of isolated single atoms (Figure 2c). HAADF‐STEM images of bimetallic **CoCu‐P** and **CoRu‐P** also show the atomic dispersion of these metals in the bimetallic systems (Figure S10, Supporting Information), and EDX mapping confirms the homogeneous distribution of the two metals in the systems (Figure 2b; Figure S4, Supporting Information). These combinations were chosen to showcase the homogeneous distribution for systems with only first row transition metals (**CoCu‐P**) and with transition metals from different rows (**CoRu‐P**) due to their disparity in size.

The Fourier‐transformed extended X‐ray absorption fine structure (EXAFS) spectra of the prepared HD‐SACs display major absorption peaks around 1.5–1.7 Å. These correspond to the M−N bonds lengths of coordinated **tatppb** (Figure 2d; Figure S11, Supporting Information). The monomers containing M−Cl bonds (Table S1, Supporting Information), exhibit peaks around 2 Å (corresponding to the metal−chloride bond), which decrease in intensity or disappear in the corresponding HD‐SACs. This change is coupled with an intensity increase of the peaks around 1.5–1.7 Å assigned to the formation of M−O bonds, indicating ligand exchange of the chloride ligands for acetates (Figure S11, Supporting Information). The substitution of chloride ligands is also observed in the Cl 2*p* edge X‐ray photoelectron spectroscopy (XPS) spectra, indicated by a decrease in the intensity or disappearance of the chloride peak. No peaks arising from metal−metal interactions in the range of 2.1–2.7 Å are observed for any HD‐SAC, further indicating the absence of metal−metal bonds, excluding the formation of clusters and nanoparticles during synthesis. XPS of the metal edge in the HD‐SACs does not show any signs of metal aggregates (Figure S12, Supporting Information), further validating the success of the polycondensation approach to produce a wide variety of HD‐SACs. Additionally, the EXAFS spectra of the monomers and HD‐SACs are strikingly similar, indicating that the coordination environment is maintained due to the mild reaction conditions employed.

The X‐ray absorption near‐edge structure (XANES) and XPS spectra of the metal edge of the HD‐SACs indicate that no elemental metal is formed during the polycondensation reaction (Table S2, Supporting Information). With the exception of **Co‐P** and **Cu‐P**, the oxidation state of the metal species in the monomer and HD‐SACs samples is comparable (Figure S13, Supporting Information), highlighting the advantage of our approach using relatively mild conditions. A slight increase in the M^2+^ oxidation state from **Co‐M** to **Co‐P** is observed in the Co K‐edge XANES spectra. In contrast, a reduction from Cu^2+^ to a mixed Cu^+1/+2^ system is observed for the copper samples at the Cu K‐edge, which is also apparent in the XPS spectra (Figure S12, Supporting Information).

To confirm that the HD‐SACs are self‐supported by the expected **tatppb** structure, further characterization was performed. The N 1*s* edge XPS spectra of the HD‐SACs show an increase in intensity and broadening of the peak compared to the monomers (Figure S14, Supporting Information), attributed to the pyrazine nitrogen atoms in the **tatppb** structure. The ^13^C solid state NMR spectra of the HD‐SACs are characterized by the disappearance of the carbonyl carbon peak around 175 ppm, which arises from the monomers (Figure S15, Supporting Information), indicating the successful polycondensation reaction. Furthermore, broadening of the remaining peaks and the appearance of new peaks are observed. These peaks are similar to those observed for the pyrazine fragment in **tatppb** (134 ppm for C−H in the central ring, 141 ppm for the imine carbon in the pyrazine fragment, and 157 ppm for the imine carbon in the phenanthroline), which confirms the formation of the expected structure (Figure S16, Supporting Information). Peaks attributable to 1,2,4,5‐benzenetetramine (124 and 120 ppm) are not observed. FTIR spectra of the HD‐SACs (Figure S17, Supporting Information) are dominated by the complete disappearance of the ν_C═O_ band of the monomers (ca. 1700 cm^−1^), indicating the successful polycondensation. Furthermore, the HD‐SACs spectra shared similarities with the spectrum of **tatppb** (Figure S18, Supporting Information). Bands corresponding to aromatic C═N and C═C bond stretching (1300–1600 cm^−1^) and bending (900–1100 cm^−1^) modes, as well as fundamental collective vibrations in the fingerprint region (assigned from computational modelling), were observed in the HD‐SACs samples (Figure S19, Supporting Information), further confirming that the expected **tatppb** structure is successfully formed during polycondensation. MALDI mass spectrometry of the HD‐SACs revealed the presence of fragment ions with the mass of **tatppb** (m/z = 487) and of some of the metals coordinated to **tatppb** (Figure S20, Supporting Information). Furthermore, no ions corresponding to unreacted metal monomers are observed. X‐band (9.5 GHz) EPR spectra of the monomers and HD‐SACs containing paramagnetic metal ions (Mn^II^, Fe^II^, Co^II^, Ni^II^, Cu^II^) were recorded (Figure S21, Supporting Information) and fitted to extract the EPR spectral parameters (Table S3, Supporting Information). The EPR spectra exhibit the expected peaks corresponding to the respective metals, and the fitted EPR parameters are consistent with the structures (see Supporting Information for more details). The EPR spectra of the HD‐SACs are more complicated than those of the monomers, and the coordination environment of the metal centers could not be fully resolved due to the broad line width. However, the nature of the metal could be confirmed, and the coordination geometry estimated. Notably, the metals in the paramagnetic HD‐SACs appear to favor lower spin, supported by the shorter M–N bond lengths of the polymers observed in the EXAFS spectra, which would result in a wider band gap. The EPR spectra of the HD‐SACs samples, expected to be diamagnetic (**Zn‐P**, **Ru‐P**, **Rh‐P**, **Pd‐P**, **Ir‐P**, and **Pt‐P**) revealed the presence of isotropic and narrow peaks, typical of organic radicals (Figure S22 and Table S3, Supporting Information). The signals could be assigned to the reduction of the **tatppb**, which can accommodate up to four electrons in its conjugated structure.^[^ ^35^, ^36^ ^]^ This phenomenon has been previously reported in transition metal complexes bearing phenanthroline ligands with extended conjugation.^[^ ^37^ ^]^

### 2.3 Automation and Scale‐Up of the HD‐SACs

**Figure 3:**
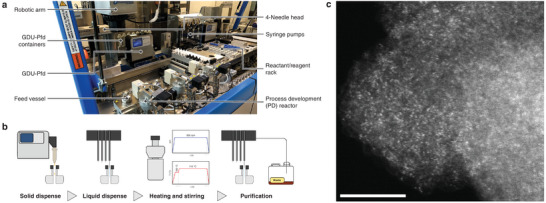
Automated HD‐SAC synthesis procedure. a) Robotic synthesis platform and tools operated, and b) flowchart of the synthetic protocol. c) HAADF‐STEM images of **Co‐P** prepared by an automated method (see Figure 2a for Co‐P prepared via the regular method). Scale bar 5 nm.

To further demonstrate the universality and robustness of the developed method, and in accordance with the current emphasis on automation and standardization of chemistry,^[^ ^38^, ^39^, ^40^, ^41^ ^]^ the synthesis of **Co‐P**, **Mn‐M**, **Co‐M**, **Cu‐M**, and **Zn‐M** was automated using a robotic platform (Figure S23 and Video S1, Supporting Information).^[^ ^42^, ^43^ ^]^ The synthesized monomers were obtained in comparable yields (see Supporting Information), and the characterization data were in agreement with that of the products obtained by conventional synthetic methods (Figures S24–S26, Supporting Information). The synthesis of **Co‐P** was automated on a gram scale using a ten‐fold scale‐up compared to the initial procedure (**Figure** **3**a,b; Video S2, Supporting Information). HAADF‐STEM confirmed the synthesis of a HD‐SAC material (Figure 3c), and no appreciable differences were observed between the materials synthesized using either the conventional or automated methods (Figures S27–S30, Supporting Information). The robust control over reaction parameters facilitates precise manipulation of the stoichiometry of multiple reagents, which could be advantageous in the preparation of heterometallic materials with differing metal ratios. In addition, the synthesis of **Co‐P** was scaled up by 10‐fold using the conventional method with comparable results (Figures S26–S29 and S31, Supporting Information), further highlighting the robustness of this approach.

### 2.4 Application of the HD‐SACs

**Figure 4:**
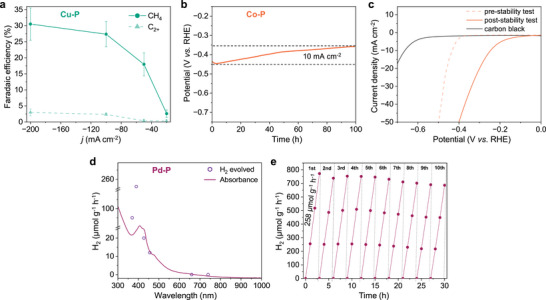
Applications of the HD‐SACs in electrocatalytic and photocatalytic applications. a) Faradaic efficiency of different CO_2_RR products with **Cu‐P** under different current densities in 1 m KOH. Error bars are shown for the measured FE of H_2_ and CH_4_. **b,** chronoamperometry analysis of the **Co‐P** with an overpotential of 0.43 V versus RHE measured at an applied current density of −10 mA cm^−2^ over 100 h and, c) LSV curves of the **Co‐P** before and after the stability test and of the carbon black support. d) Sacrificial agent free photocatalytic OWS activity of **Pd‐P** irradiated at different wavelengths and UV–vis absorbance spectra. e) Long‐term photocatalytic OWS with **Pd‐P**.

SACs are widely utilized in electrocatalysis as their highly dispersed single‐metal sites enhance catalytic activity while achieving excellent product selectivity due to their unique structural properties.^[^ ^44^, ^45^ ^]^ However, atomically dispersed metallic sites often suffer from the aggregation of the metal atoms.^[^ ^46^ ^]^ Among our library of HD‐SACs, the **Cu‐P** catalyst was evaluated for CO_2_ reduction reaction (CO_2_RR), as atomically dispersed Cu has been shown to be a promising source to catalyze CO_2_RR to hydrocarbons and oxygenates.^[^ ^47^, ^48^, ^49^ ^]^ Especially, atomic Cu sites are often prone to aggregation to Cu clusters,^[^ ^50^, ^51^, ^52^ ^]^ where at least three or more Cu atoms are required to generate C_2+_ products.^[^ ^53^ ^]^ Therefore, the product distribution of **Cu‐P** during CO_2_RR gives an indication of the stability of the atomic site. **Cu‐P** achieved a high selectivity of CO_2_ to CH_4_ with negligible selectivity to C_2_H_4_ (**Figure** **4**a) in 1 m aqueous KOH. The lack of C−C coupling products supports that the single‐atom distribution is maintained during operation. At commercially relevant current densities (≈−200 mA cm^−2^), the catalyst achieves a Faradaic efficiency (FE) for CH_4_ of 31 ± 5%, whereas the FE of other carbon products was not significant (Figure S32, Supporting Information). At lower current densities, **Cu‐P** also promotes the competing hydrogen evolution reaction (HER), which is typical of Cu‐based electrocatalysts.^[^ ^54^ ^]^ However, the excellent selectivity to a major carbon product (CH_4_) at higher current densities demonstrates the stability of the single‐atom sites in the developed **Cu‐P**. The stability of **Cu‐P** under CO_2_RR was assessed for 3 h of continuous reaction at −200 mA cm^−2^ (Figure S33a, Supporting Information). No significant changes in conversion or selectivity were observed. Additionally, **Cu‐P** did not show any signs of aggregation under HAADF‐STEM, confirming the stability of the HD‐SAC (Figure S33b, Supporting Information). The stability of **Co‐P** was assessed as a model catalyst for HER due to its high activity as a non‐noble metal electrocatalyst.^[^ ^55^ ^]^ Notably, the catalyst maintains a steady overpotential over 100 h of continuous operation (Figure 4b), demonstrating the excellent stability of the single atom sites in **Co‐P**. HAADF‐STEM images of **Co‐P** after continuous operation for 100 h showed that the atomic dispersion remains intact and that no aggregates are observed (Figure S34, Supporting Information). The enhancement observed in the electrochemical stability is in accordance with the improved activity from the linear sweep voltammetry (LSV) curves (Figure 4c), where an overpotential of 0.24 V versus reversible hydrogen electrode (RHE) was achieved. The improvement in activity is likely due to activation of the metal sites over time, presumably through ligand dissociation reactions, which lead to active Co−N_6−x_ sites. The stability of **Co‐P** was also evaluated at higher current densities (−200 mA cm^−2^) over 5 h (Figure S35a, Supporting Information), which did not lead to any aggregation of Co‐sites, further confirming the inherent stability of the produced HD‐SACs (Figure S35b, Supporting Information).

The catalytic performance and durability of the HD‐SACs were systematically evaluated for their activity in the photocatalytic overall water splitting (OWS). Among the synthesized HD‐SACs, **Pd‐P** was identified as a promising candidate due to its underexplored potential in OWS applications, where Pd is conventionally incorporated into composite systems with other co‐catalysts or used alongside sacrificial agents to enhance overall photocatalytic activity.^[^ ^56^, ^57^, ^58^ ^]^ Remarkably, **Pd‐P** achieved a hydrogen evolution rate of 258 µmol g^−1^ h^−1^ under 390 nm irradiation (Figure 4d), which overperforms other reported sacrificial‐agent‐free, palladium catalysts and SAC for OWS (Table S4, Supporting Information). Notably, **Pd‐P** facilitated OWS without the use of an external sacrificial agent, which is conventionally employed in photocatalytic systems to mitigate charge recombination or serve as an electron donor.^[^ ^59^, ^60^, ^61^, ^62^, ^63^, ^64^ ^]^ Furthermore, the **Pd‐P** catalyst exhibits exceptional stability, maintaining consistent performance over ten consecutive cycles with only a slight reduction in performance (Figure 4e). No Pd‐aggregates were observed under HAADF‐STEM, highlighting the stability of Pd‐sites during continuous operation (Figure S36a, Supporting Information), and no changes were observed in the EPR spectra of **Pd‐P** after operation (Figure S36b, Supporting Information). The valence band (VB) energy and the band gap of **Pd‐P** were estimated from UV Photoelectron Spectroscopy (UPS) and UV–vis spectroscopy measurements, respectively (Figure S37, Supporting Information). The VB energy of **Pd‐P** is 4.18 ± 0.03 eV, and the band gap was estimated to be 1.83 eV. Therefore, the VB and conduction band (CB) energies were −0.32 and +1.51 V (vs NHE), respectively. These values are lower and higher, respectively, than the half‐reaction potentials of the HER (0 V) and OER (+1.23 V), consistent with the photocatalyzed OWS reaction catalyzed by **Pd‐P**. Photoluminescence (PL) spectroscopy reveals quenched emission intensity for **Pd‐P**, indicating suppressed electron‐hole recombination and suggesting enhanced charge separation efficiency, which is critical for photocatalytic activity (Figure S38, Supporting Information). Intriguingly, **Pd‐P** exhibits a broad impedance arc as seen in Electrochemical impedance spectroscopy (EIS) (Figure S38, Supporting Information). Despite this and the low electrical conductivity of the materials, **Pd‐P** demonstrated appreciable photocatalytic performance, which may be attributed to the presence of ligand‐centered redox‐active states.^[^ ^65^ ^]^ EPR spectroscopy provided evidence for these states, which could facilitate local charge storage and transfer through hopping or redox‐mediated mechanisms, enabling photogenerated carriers to participate in catalytic redox reactions without necessitating long‐range bulk conductivity (Figure S22, Supporting Information).^[^ ^58^ ^]^ The combination of these properties underscores the intrinsic efficiency of **Pd‐P** as an HD‐SAC for sustainable hydrogen production under visible‐light‐driven, sacrificial agent‐free photocatalytic conditions.

## 3 Conclusion

We have developed a universal bottom‐up polycondensation approach for the synthesis of HD‐SACs with controlled atomic dispersion, metal coordination environments, and high stability. This strategy enables the synthesis of a diverse range of mono‐ and bimetallic HD‐SACs, employing pre‐anchored metal monomers and a self‐supporting conjugated ligand framework, therefore preventing aggregation and ensuring atomic‐level dispersion. The synthesized materials demonstrate high stability in electrocatalysis and photocatalysis. Moreover, the scalability and adaptability of the method to automation highlight its potential for industrial production and future materials discovery. This work not only advances the rational design of HD‐SACs but also establishes a reproducible and modular approach for the systematic development of mono‐ and multimetallic catalytic systems.

## 4 Experimental Section

The synthesis and characterization of the metal monomers are available in the Supporting Information. Full characterization of the HD‐SACs, including XAS, XPS, FTIR, ssNMR, HAADF‐STEM, and elemental mapping, and catalytic procedures, is also available.

### HD‐SAC Synthesis

The appropriate metal precursor (0.15 mmol, 1 eq., for monomers with two 1,10‐phenanthroline‐5,6‐dione ligands, or 0.10 mmol, 0.67 eq., for monomers with three 1,10‐phenanthroline‐5,6‐dione ligands) and 1,2,4,5‐benzenetetramine tetrahydrochloride (42 mg, 0.15 mmol, 1 eq.) were loaded in a 10 mL glass vial which was closed with an aluminum cap with a septum. The solids were suspended in degassed EtOH (0.9 mL) and degassed acetic acid (3.02 mL, 53 mmol, ≈350 eq.), and the mixture was stirred for 30 min at 100 °C. Afterward, Et_3_N (150 µL, 8.6 mmol, 7.2 eq.) was added and the mixture was further stirred at 115 °C for 72 h. The suspension was filtered through a nylon membrane filter (0.45 µm), washed with H_2_O (100 mL) and methanol (25 mL), and dried under vacuum at 70 °C for 24 h. Yields are consistent and between 60 and 100 mg of HD‐SAC are obtained at this reaction scale, depending on the metal loading and the architecture arising from the corresponding metal monomer (see Supporting Information for additional details).

## Author Contributions

J.‐C.S. and J.R.‐dG. contributed equally to this work. J.R.‐dG. and J.‐C.S. conceived the idea, analyzed and interpreted the data, and led the project. J.R.‐dG. and J.‐C.S. wrote the manuscript with input from all other authors. J.R.‐dG., J.‐C.S., R.J.S., and XH.W. conducted the synthesis and characterization of the precursors and materials. D.C.S. and M.M. measured and analyzed the XAS and XPS data, respectively. M.C., A.S., and XB.W. carried out the electrochemical experiments. S.W. and S.D. studied the photocatalytic OWS reaction. S.D.L. and P.M. developed the automation of the monomer and HD‐SACs synthesis. N.M. quantified the electrical conductivity of the samples. D.O. executed the MS analysis. J.R.‐dG. and L.P. conducted the ssNMR and EPR spectroscopy experiments. D.R. was in charge of the high‐resolution HAADF‐STEM measurements. L.E.K.F. performed SEM characterization. J.E. and T.M.O.F. carried out the BET surface area measurements. All authors contributed to the discussion of the results and revision of the manuscript. P.J.D. directed and supervised the project.

## Conflict of Interest

The authors declare no conflict of interest.

## Supporting information



Supporting Information

Supplemental Video 1

Supplemental Video 2

## Data Availability

The data that support the findings of this study are available in the supplementary material of this article. Additional raw data generated in this study is openly available at https://doi.org/10.5281/zenodo.15525104.

## References

[1] S. Singh , R. Kumar , H. D. Setiabudi , S. Nanda , D.‐V. N. Vo , Appl. Catal., A 2018, 559, 57.

[2] Z. Shi , X. Wang , J. Ge , C. Liu , W. Xing , Nanoscale 2020, 12, 13249. 32568352 10.1039/d0nr02410d

[3] J. C. Védrine , ChemSusChem 2019, 12, 577.30496640

[4] L. Han , S. Cai , M. Gao , J. Hasegawa , P. Wang , J. Zhang , L. Shi , D. Zhang , Chem. Rev. 2019, 119, 10916. 31415159 10.1021/acs.chemrev.9b00202

[5] I. M. Rizwanul Fattah , H. C. Ong , T. M. I. Mahlia , M. Mofijur , A. S. Silitonga , S. M. A. Rahman , A. Ahmad , Front. Energy Res. 2020, 8, 101.

[6] J. Martínez , A. Ortiz , I. Ortiz , Appl. Catal., B 2017, 207, 42.

[7] C. Xie , Z. Niu , D. Kim , M. Li , P. Yang , Chem. Rev. 2020, 120, 1184. 31580651 10.1021/acs.chemrev.9b00220

[8] D. Astruc , Chem. Rev. 2020, 120, 461. 31964144 10.1021/acs.chemrev.8b00696

[9] B. Wu , N. Zheng , Nano Today 2013, 8, 168.

[10] L. Lin , W. Zhou , R. Gao , S. Yao , X. Zhang , W. Xu , S. Zheng , Z. Jiang , Q. Yu , Y.‐W. Li , C. Shi , X.‐D. Wen , D. Ma , Nature 2017, 544, 80. 28329760 10.1038/nature21672

[11] S. Ding , M. J. Hülsey , J. Pérez‐Ramírez , N. Yan , Joule 2019, 3, 2897.

[12] Z. Li , S. Ji , Y. Liu , X. Cao , S. Tian , Y. Chen , Z. Niu , Y. Li , Chem. Rev. 2020, 120, 623. 31868347 10.1021/acs.chemrev.9b00311

[13] B. Singh , V. Sharma , R. P. Gaikwad , P. Fornasiero , R. Zbořil , M. B. Gawande , Small 2021, 17, 2006473. 10.1002/smll.202006473 33624397

[14] S. K. Kaiser , Z. Chen , D. Faust Akl , S. Mitchell , J. Pérez‐Ramírez , Chem. Rev. 2020, 120, 11703. 33085890 10.1021/acs.chemrev.0c00576

[15] X.‐F. Yang , A. Wang , B. Qiao , J. Li , J. Liu , T. Zhang , Acc. Chem. Res. 2013, 46, 1740. 23815772 10.1021/ar300361m

[16] Y. Li , Z. Wu , P. Lu , X. Wang , W. Liu , Z. Liu , J. Ma , W. Ren , Z. Jiang , X. Bao , Adv. Sci. 2020, 7, 1903089. 10.1002/advs.201903089 PMC7055577 32154084

[17] B. Qiao , A. Wang , X. Yang , L. F. Allard , Z. Jiang , Y. Cui , J. Liu , J. Li , T. Zhang , Nature Chem 2011, 3, 634. 21778984 10.1038/nchem.1095

[18] Y. Gu , B. Xi , W. Tian , H. Zhang , Q. Fu , S. Xiong , Adv. Mater. 2021, 33, 2100429. 10.1002/adma.202100429 33998069

[19] T. Zheng , K. Jiang , N. Ta , Y. Hu , J. Zeng , J. Liu , H. Wang , Joule 2019, 3, 265.

[20] L. Zhang , M. Zhou , A. Wang , T. Zhang , Chem. Rev. 2020, 120, 683. 31549814 10.1021/acs.chemrev.9b00230

[21] A. Wang , J. Li , T. Zhang , Nat. Rev. Chem. 2018, 2, 65.

[22] Y. Xue , B. Huang , Y. Yi , Y. Guo , Z. Zuo , Y. Li , Z. Jia , H. Liu , Y. Li , Nat. Commun. 2018, 9, 1460. 29654234 10.1038/s41467-018-03896-4 PMC5899097

[23] J. Wang , Z. Li , Y. Wu , Y. Li , Adv. Mater. 2018, 30, 1801649. 10.1002/adma.201801649 30276871

[24] S. Ji , Y. Chen , X. Wang , Z. Zhang , D. Wang , Y. Li , Chem. Rev. 2020, 120, 11900. 32242408 10.1021/acs.chemrev.9b00818

[25] X. Hai , S. Xi , S. Mitchell , K. Harrath , H. Xu , D. F. Akl , D. Kong , J. Li , Z. Li , T. Sun , H. Yang , Y. Cui , C. Su , X. Zhao , J. Li , J. Pérez‐Ramírez , J. Lu , Nat. Nanotechnol. 2022, 17, 174. 34824400 10.1038/s41565-021-01022-y

[26] Y. Wang , C. Li , X. Han , J. Bai , X. Wang , L. Zheng , C. Hong , Z. Li , J. Bai , K. Leng , Y. Lin , Y. Qu , Nat. Commun. 2024, 15, 5675. 38971885 10.1038/s41467-024-50061-1 PMC11227521

[27] C. Xia , Y. Qiu , Y. Xia , P. Zhu , G. King , X. Zhang , Z. Wu , J. Y. Kim , D. A. Cullen , D. Zheng , P. Li , M. Shakouri , E. Heredia , P. Cui , H. N. Alshareef , Y. Hu , H. Wang , Nat. Chem. 2021, 13, 887. 34168326 10.1038/s41557-021-00734-x

[28] J. Chang , W. Jing , X. Yong , A. Cao , J. Yu , H. Wu , C. Wan , S. Wang , G. I. N. Waterhouse , B. Yang , Z. Tang , X. Duan , S. Lu , Nat. Synth 2024, 3, 1427.

[29] F. Xie , X. Cui , X. Zhi , D. Yao , B. Johannessen , T. Lin , J. Tang , T. B. F. Woodfield , L. Gu , S.‐Z. Qiao , Nat. Synth 2023, 2, 129.

[30] H. Li , F. Pan , C. Qin , T. Wang , K. Chen , Adv. Energy Mater. 2023, 13, 2301378.

[31] Z.‐J. Gong , Y. S. L. V. Narayana , Y.‐C. Lin , W.‐H. Huang , W.‐N. Su , Y.‐P. Li , M. Higuchi , W.‐Y. Yu , Appl. Catal., B 2022, 312, 121383.

[32] A. Winter , U. S. Schubert , Chem. Soc. Rev. 2016, 45, 5311. 27218823 10.1039/c6cs00182c

[33] N. K. , C. S. Rout , RSC Adv. 2021, 11, 5659. 35686160 10.1039/d0ra07800j PMC9133880

[34] T.‐H. Le , Y. Kim , H. Yoon , Polymers 2017, 9, 150.30970829

[35] N. R. De Tacconi , R. O. Lezna , R. Konduri , F. Ongeri , K. Rajeshwar , F. M. MacDonnell , Chemistry A European J 2005, 11, 4327. 10.1002/chem.200401287 15887195

[36] W. Guo , S. O. Obare , Tetrahedron Lett. 2008, 49, 4933.

[37] A. Klein , W. Kaim , E. Waldhör , H.‐D. Hausen , J. Chem. Soc., Perkin Trans. 2 1995, 2121.

[38] P. Q. Velasco , K. Hippalgaonkar , B. Ramalingam , Beilstein J. Org. Chem. 2025, 21, 10. 39811684 10.3762/bjoc.21.3 PMC11730176

[39] S. P. Schmid , L. Schlosser , F. Glorius , K. Jorner , Beilstein J. Org. Chem. 2024, 20, 2280. 39290209 10.3762/bjoc.20.196 PMC11406055

[40] T. Dai , S. Vijayakrishnan , F. T. Szczypiński , J.‐F. Ayme , E. Simaei , T. Fellowes , R. Clowes , L. Kotopanov , C. E. Shields , Z. Zhou , J. W. Ward , A. I. Cooper , Nature 2024, 635, 890. 39506122 10.1038/s41586-024-08173-7 PMC11602721

[41] M. Eisenstein , Nature 2025, 637, 1008. 39833414 10.1038/d41586-025-00075-6

[42] P. Laveille , P. Miéville , S. Chatterjee , E. Clerc , J.‐C. Cousty , F. De Nanteuil , E. Lam , E. Mariano , A. Ramirez , U. Randrianarisoa , K. Villat , C. Copéret , N. Cramer , Chimia 2023, 77, 154. 38047820 10.2533/chimia.2023.154

[43] A. Ramirez , E. Lam , D. P. Gutierrez , Y. Hou , H. Tribukait , L. M. Roch , C. Copéret , P. Laveille , Chem Catalysis 2024, 4, 100888.

[44] Q. Zhang , J. Guan , Adv. Funct. Mater. 2020, 30, 2000768.

[45] T. Sun , S. Mitchell , J. Li , P. Lyu , X. Wu , J. Pérez‐Ramírez , J. Lu , Adv. Mater. 2021, 33, 2003075. 10.1002/adma.202003075 33283369

[46] Y. Yang , Y. Yang , Z. Pei , K.‐H. Wu , C. Tan , H. Wang , L. Wei , A. Mahmood , C. Yan , J. Dong , S. Zhao , Y. Chen , Matter 2020, 3, 1442.

[47] H. Xie , T. Wang , J. Liang , Q. Li , S. Sun , Nano Today 2018, 21, 41.

[48] Y. Li , B. Guan , Z. Zhuang , J. Chen , L. Zhu , Z. Ma , X. Hu , C. Zhu , S. Zhao , K. Shu , H. Dang , T. Zhu , Z. Huang , Adv. Funct. Mater. 2025, 35, 2417732.

[49] Y. Dai , H. Li , C. Wang , W. Xue , M. Zhang , D. Zhao , J. Xue , J. Li , L. Luo , C. Liu , X. Li , P. Cui , Q. Jiang , T. Zheng , S. Gu , Y. Zhang , J. Xiao , C. Xia , J. Zeng , Nat. Commun. 2023, 14, 3382. 37291114 10.1038/s41467-023-39048-6 PMC10250324

[50] D. Karapinar , N. T. Huan , N. Ranjbar Sahraie , J. Li , D. Wakerley , N. Touati , S. Zanna , D. Taverna , L. H. Galvão Tizei , A. Zitolo , F. Jaouen , V. Mougel , M. Fontecave , Angew. Chem., Int. Ed. 2019, 58, 15098. 10.1002/anie.201907994 31453650

[51] C. E. Creissen , M. Fontecave , Nat. Commun. 2022, 13, 2280. 35477712 10.1038/s41467-022-30027-x PMC9046394

[52] H. Xu , D. Rebollar , H. He , L. Chong , Y. Liu , C. Liu , C.‐J. Sun , T. Li , J. V. Muntean , R. E. Winans , D.‐J. Liu , T. Xu , Nat. Energy 2020, 5, 623.

[53] J. Zhang , Y. Wang , Y. Li , J. Am. Chem. Soc. 2024, 146, 14954. 38804682 10.1021/jacs.4c05669

[54] G. L. De Gregorio , T. Burdyny , A. Loiudice , P. Iyengar , W. A. Smith , R. Buonsanti , ACS Catal. 2020, 10, 4854. 32391186 10.1021/acscatal.0c00297 PMC7199425

[55] R. Sun , X. Huang , J. Jiang , W. Xu , S. Zhou , Y. Wei , M. Li , Y. Chen , S. Han , Dalton Trans. 2022, 51, 15205. 36125033 10.1039/d2dt02189g

[56] B. Rusinque , S. Escobedo , H. De Lasa , Catalysts 2021, 11, 405.

[57] L. Liu , X. Wu , L. Wang , X. Xu , L. Gan , Z. Si , J. Li , Q. Zhang , Y. Liu , Y. Zhao , R. Ran , X. Wu , D. Weng , F. Kang , Commun Chem 2019, 2, 18.

[58] Y. Bai , C. Li , L. Liu , Y. Yamaguchi , M. Bahri , H. Yang , A. Gardner , M. A. Zwijnenburg , N. D. Browning , A. J. Cowan , A. Kudo , A. I. Cooper , R. S. Sprick , Angew Chem Int Ed 2022, 61, 202201299. 10.1002/anie.202201299 PMC9321674 35377540

[59] J. Liu , Y. Liu , N. Liu , Y. Han , X. Zhang , H. Huang , Y. Lifshitz , S.‐T. Lee , J. Zhong , Z. Kang , Science 2015, 347, 970. 25722405 10.1126/science.aaa3145

[60] Q. Wang , T. Hisatomi , Q. Jia , H. Tokudome , M. Zhong , C. Wang , Z. Pan , T. Takata , M. Nakabayashi , N. Shibata , Y. Li , I. D. Sharp , A. Kudo , T. Yamada , K. Domen , Nature Mater 2016, 15, 611. 26950596 10.1038/nmat4589

[61] M. G. Kibria , F. A. Chowdhury , S. Zhao , B. AlOtaibi , M. L. Trudeau , H. Guo , Z. Mi , Nat. Commun. 2015, 6, 6797. 25854846 10.1038/ncomms7797

[62] T. Takata , J. Jiang , Y. Sakata , M. Nakabayashi , N. Shibata , V. Nandal , K. Seki , T. Hisatomi , K. Domen , Nature 2020, 581, 411. 32461647 10.1038/s41586-020-2278-9

[63] Z. Wang , C. Li , K. Domen , Chem. Soc. Rev. 2019, 48, 2109. 30328438 10.1039/c8cs00542g

[64] C. Avcıoǧlu , S. Avcıoǧlu , M. F. Bekheet , A. Gurlo , ACS Appl. Energy Mater. 2023, 6, 1134.

[65] M. Avais , R. M. Thakur , E. Fox , J. L. Lutkenhaus , E. B. Pentzer , Chem. Sci. 2025, 16, 8357. 40213372 10.1039/d5sc00051c PMC11980798

