# Supplementary material for: Polycondensation as a Universal Method for Preparing High‐Density Single‐Atom Catalyst Libraries
Source: Adv Mater. 2025 Jul 4;37(38):2507627. doi: 10.1002/adma.202507627 (PMC12464651; doi:10.1002/adma.202507627)
Supplement: Supplementary file 1 — Supporting Information [file ADMA-37-2507627-s001.pdf]

ADVANCED MATERIALS

Supporting Information

for *Adv. Mater.*, DOI 10.1002/adma.202507627

Polycondensation as a Universal Method for Preparing High-Density Single-Atom Catalyst Libraries

Jaques-Christopher Schmidt, Jan Romano-deGea, Dragos C. Stoian, Mounir Mensi, Miyeon Chang, Ariana Serban, Satyadeep Waiba, Xinbang Wu, Lindsey E. K. Frederiksen, Rosie J. Somerville, Roland C. Turnell-Ritson, Xunhui Wang, Laura Piveteau, Daniel Ortiz, Niccolò Martinolli, David Reyes, Jordi Espín, Timo M. O. Felder, Stefano Di Leone, Pascal Miéville, Shoubhik Das and Paul J. Dyson**

Supporting Information

Polycondensation as a universal method for preparing high-density single atom catalyst libraries

Jaques-Christopher Schmidt[1†], Jan Romano-deGea[1†], Dragos C. Stoian[2], Mounir Mensi[1], Miyeon Chang[1], Ariana Serban[1], Satyadeep Waiba[3], Xinbang Wu[1], Lindsey E. K. Frederiksen[1], Rosie J. Somerville[1], Roland C. Turnell-Ritson[4], Xunhui Wang[1], Laura Piveteau[1], Daniel Ortiz[1], Niccolò Martinolli[5], David Reyes[6], Jordi Espín[1], Timo M. O. Felder[1], Stefano Di Leone[7], Pascal Miéville[7], Shoubhik Das[3], and Paul J. Dyson[1*]*

[†]These authors contributed equally: Jaques-Christopher Schmidt and Jan Romano-deGea.

Table of Contents

Materials and methods 2
Materials 2
Methods 2
Synthesis and characterization 7
Synthesis of 9,11,20,22-tetraazatetrapyrido [3,2-*a*:2‘,3‘-*c*:3‘ ‘,2‘ ‘-l:2‘ ‘‘,3‘ ‘‘-*n*]pentacene (tatppb) 7
Synthesis of the metal monomers 8
Automated synthesis of Mn-M, Co-M, Cu-M and Zn-M 14
HD-SAC polymer synthesis 14
Automated polymer synthesis of Co-P 16
Catalytic studies 17
Supporting figures 21
Supporting spectra 57
Supporting references 70

Materials and methods

Materials

All chemicals were either of reagent or analytical grade and were used as purchased from commercial sources without additional purification. 1,10-phenanthroline-5,6-dione was obtained from Combi-Blocks. 1,2,4,5-benzenetetramine tetrahydrochloride was obtained from Fluorochem. $MnCl_2 \cdot 4H_2O$, $FeSO_4 \cdot 6H_2O$, $CoCl_2$, $Ni(OAc)_2 \cdot 4H_2O$, $CuCl_2 \cdot 2H_2O$, $ZnCl_2$, $Pd(NO_3)_2$ were obtained from Sigma Aldrich. $RuCl_3{\cdot}xH_2O$, $RhCl_3{\cdot}xH_2O$, $IrCl_3{\cdot}xH_2O$, and K_2PtCl_4 were obtained from Precious Metals Online (PMO). Reactions were performed under an inert atmosphere (N_2) using standard Schlenk techniques with solvents dried using a solvent purification system (Innovative Technologies Inc.) or dried over molecular sieves (3 Å). $Ru(DMSO)_4Cl_2$[1] and $Pt(DMSO)_2Cl_2$[2] were synthesized according to literature procedures.

Methods

NMR spectroscopy: Solution NMR spectra were acquired on a Bruker 400 MHz spectrometer (9.4 T) equipped with a console AVIII HD and a 5 mm liquid-state BBO Z-gradient field three channel ($^1H/^2H$/BBF) probe head. Experiments were performed at room temperature while locking to the deuterium signal of the d_6-DMSO. ^1H and ^{13}C chemical shifts were referenced to $Si(CH_3)_4$ ($\delta(^1H, ^{13}C) = 0$ ppm) using the signals of the residual protons and of the ^{13}C of the deuterated solvent as secondary reference. Chemical shifts are reported in ppm and coupling constants (J) are reported in Hz. The following abbreviations were used to designate multiplicities: s = singlet, d = doublet, t = triplet, q = quartet, quint = quintuplet, sept = septuplet, hept = heptuplet, m = multiplet, and combination thereof. One-dimensional (1D) ^1H and ^{13}C spectra were acquired using the standard pulse sequences from the Bruker library. NMR spectra were processed with MestreNova 14.2.1 (Mestrelab Research S.L.) and TopSpin 4.2.0 (Bruker).

Solid-state ^{13}C magic angle spinning (MAS) NMR spectra were recorded on 400 MHz and 500 MHz Bruker spectrometers (9.4 and 11.7 T) equipped with an Avance III HD consoles and 3,2 mm HXY triple channel CPMAS probes. Samples were packed into 3.2 mm zirconia rotors under ambient conditions and spun at 15-20 kHz using dry nitrogen gas at room temperature. Whenever possible, ^{13}C CP MAS spectra were obtained by polarization transfer from ^1H to ^{13}C to enhance the signal of the latter. The spectra were recorded using variable amplitude during contact times of 1 ms.[3] Recycling delays were set to 1.3*T_1 of the protons and high-power proton decoupling was applied during acquisition of all spectra using the spin64 pulse sequence.[4] Between 8192 and 32768 transients were summed to obtain the presented the spectra. If no signal was obtained by CP, ^{13}C one-pulse or Hahn echo sequences,

followed by high-power proton decoupling during acquisition using the spin64 pulse sequence were applied.[4] τ_{90} pulse lengths were in the range of 3.4-3.8 µs and were rotor synchronized to two rotor periods. Up to 20480 scans were cumulated for the displayed spectra. ^{13}C chemical shifts were referenced to the secondary reference adamantane, whose more deshielded signal appears at 38.48 ppm.

EPR spectroscopy: EPR spectra were measured using a Bruker EMX nano X-band (9 GHz) spectrometer using a 0.4 mT modulation amplitude, a 100 kHz modulation frequency, and a 0.3 mW of microwave power. Sealed glass capillaries containing the solid samples were placed in a standard 4 mm quartz sample tube. The temperature of the sample was regulated and maintained throughout the measurement by a nitrogen evaporator thermostat. Measurements were carried at 100 or 293 K. 1D field/sweep experiments were acquired using the standard transition metal settings included in the Bruker instrument library: the instrument field was centered at 3200 G, the sweep width was set to 5600.4 G and the time constant to 4.29 ms. EPR spectra were measured with an attenuation of 25 dB (~20 mW) to avoid microwave saturation of the resonance absorption curve. The number of scans was typically set to 16, to improve the signal-to-noise ratio. The Xenon software (Bruker) was used for baseline correction and the EasySpin 6.0 suite in MATLAB for fitting and spectral simulation.

High resolution mass spectra (HRMS): HRMS spectra were acquired using either a Thermo Orbitrap Elite instrument with an LTQ-Orbitrap analyzer or a Waters XEVO G2-S QTOF instrument with a QTOF analyzer.

Matrix-assisted laser desorption/ionization time-of-flight mass spectrometry (MALDI-TOF-MS): MALDI-TOF-MS analysis was conducted using a Bruker Autoflex system. Analyte (1 mg) and DHB matrix (2,5-dihydroxybenzoic acid, 1 mg) were weighed separately and transferred into a glass vial. Subsequently, 100 µL of dichloromethane was added, and the mixture was vortexed for approximately 30 seconds. Immediately thereafter, 1 µL of the undiluted sample was deposited onto a plate well, lightly flattened using a microspatula to achieve flush alignment with the surface of the target, and allowed to air-dry completely. Typically, a single spectrum was acquired by firing 1000 laser shots at 75% of the maximum laser power. Spectra were calibrated and referenced to a PEG700 sample.

Elemental analysis (EA): EA was performed using a Thermo Scientific Flash 2000 Organic Elemental Analyser.

Powder X-ray diffraction (pXRD): pXRD of all polymers was recorded from $2\theta = 1–75°$ on a Bruker D8 Discover Vario diffractometer by using Cu Kα (L = 1.54 Å)

radiation that was operated at 40 kV and 40 mA with an angular step size of 0.002° and a counting time of 12 s per step.

Fourier transformed infrared (FTIR) spectroscopy: IR spectra (400–4000 cm^{-1}) were recorded on a Perkin Elmer Spectrum Two ATR-FTIR spectrometer. The following abbreviations were used to describe the bands: s = strong, m = medium, w = weak, br = broad. Assignment of vibrational modes is based on available literature and supported by computational assignments: ν = stretching, δ = bending, ρ = rocking, ω = wagging, τ = twisting, as = asymmetric, s = symmetric.

Inductively coupled plasma mass spectrometry (ICP-MS): (ICP-MS) samples were weighted and submitted to acidic digestion with 4 mL of aqua regia consisting of concentrated acids HNO_3 (69%, ROTIPURAN Supra, Roth) and HCl (35%, ROTIPURAN Supra, Roth) freshly mixed in 1:3 ratio. Digestion was performed using microwave oven (ETHOS.lab, MLS MWS Mikrowelle GmbH) with the following program: 15 minutes to heat up to 210°C and keep at 210°C for 25 minutes. After the digestion the samples were diluted 300 times with 2% HNO_3 solution and metals of interest were quantified by ICP-MS using KED mode with He as a collision gas on NexIon 350 D ICP-MS instrument (PerkinElmer). Yttrium was added as an internal standard at concentration of 2 ppb to all the solution and metals quantitation was performed using external calibration curve with standards in 0.05-50 ppb range. All measurements were performed in triplicate.

Scanning transmission electron microscopy (STEM): STEM images for all polymers were obtained on a FEI Tecnai Osiris electron microscope operating at 200 KeV, and high resolution (HR) STEM images for all polymers were obtained on a FEI Titan Themis electron microscope operating at 300 keV using a convergence angle of 20 mrad. A High-angle annular dark field (HAADF) detector was used for all STEM imaging. Energy-dispersive X-ray spectroscopy (EDX) was performed in the range 0-20 or 0-40 KeV with a dispersion of 10 and 20 eV respectively.

Thermogravimetric analysis (TGA)/Differential scanning calorimetry (DSC)/Mass spectrometry (MS): TGA of the polymers under an inert nitrogen atmosphere were performed on a Netzsch TGA/DSC/MS instrument (STA 449 F3 Jupiter and QMS 403 Aeolos Quadro). Around 30 mg of the sample was filled in an Al_2O_3 85 mL crucible. The sample was first purged under N_2 with a flow rate of 70 mL min^{-1} for 30 minutes at 30 °C. Afterwards the sample was heated to 900 °C (5 °C min^{-1}) and held for 30 minutes. During measurements, the change in mass and the heat flux by differential scanning calorimetry were recorded. The measurement was background corrected by measuring the empty crucible. The evolved gases were analyzed by mass spectrometry within the range m/z 16-44 (step width of 1 amu,

settling time of 20 ms, integration time of 20 ms). For the TGA of the polymers under oxidizing atmosphere, the sample was first purged under N_2 with a flow rate of 70 mL min^{-1} for 30 minutes at 30 °C. Afterwards, diluted oxygen (5% O_2 in N_2 at 72 mL min-1) was introduced and the sample was heated to 900 °C (5 °C min^{-1}) and held for 30 minutes.

BET surface area measurements: Nitrogen adsorption measurements were performed on a Belsorp Max-II instrument at 77 K. The samples were activated at 125°C for 12 hours under vacuum prior to the measurement. The specific surface area was calculated by the BET method using BETSI software.[5]

Conductivity measurements: Volume conductivity measurements of the pressed polymer pellets were performed using a standard three-electrode configuration, as explained by Blythe.[6] Briefly, a voltage V was applied on an electrode contacted with the entire bottom surface of the polymer disk. On the top surface two electrodes were placed, an outer ring and an inner disk, not touching each other. Both top electrodes were forced to 0 V and the current I flowing from the inner disc was measured. This current is assumed to flow through the portion of polymer pellet beneath the inner disk, in accordance with a perpendicular electric field lines approximation. The resistivity of the polymer pellet, then, depends on the measured resistance, the area of the top inner electrode and the pellet thickness, measured with a caliper, through the formula $\rho = R \cdot \frac{\pi \cdot r^2}{t} = \frac{1}{\sigma}$, where ρ [Ω·cm] is the volume electrical resistivity, R [Ω] is the extrapolated resistance corresponding to the reciprocal of the slope of the line interpolating the data points in a I [A] – V [V] plot (measured I, V swept from -5 to +5 V), r [cm] is the radius of the inner top electrode, t [cm] is the thickness of the polymer pellet, and σ [S·cm^{-1}] is the volume electrical conductivity. In the analyzed voltage range, the I – V plots are linear and the intercept reasonably close to zero, suggesting the absence of transient components.

Standard 3-electrode measurement systems for high-resistivity materials rely on metal or conductive rubber electrodes pressed on the polymer disc or on blade electrodes. Because of the fragility of the polymer pellets, these systems were not employable. Instead, a silicon chip with 100 nm silicon oxide layer was used as a substrate. Four tungsten pads were deposited by sputtering and lift-off, leaving enough space for the polymer pellet among them. A bottom layer of conductive silver paste was spread, connecting the four pads, and served both as bottom electrode and as a glue to attach the pellet to the substrate. The top outer ring served as a guard electrode to exclude any spurious current due to surface conduction, where dirt and moisture play a role. Turned aluminum disk and ring were used as top electrodes, also glued to the pellet by silver paste. A tungsten pad and the aluminum top electrodes were finally

contacted with tungsten probes in a Microtech Cascade Summit probe station, and the electrical measurements were acquired with a Keithley 4200A-SCS parameter analyzer. Both the pad-to-pad resistance and the resistance across two opposite points of the ring electrode were measured and found to be of a few Ω, negligible with respect to the resistance of the polymer pellet itself. The contact resistance between the pellet and the silver paste is also assumed to be small, justifying not using a 4-probe setup.

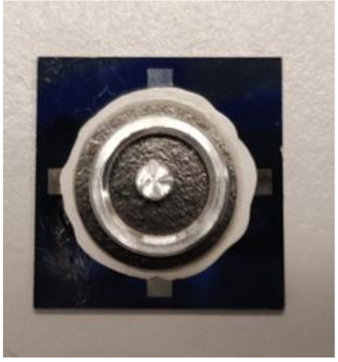
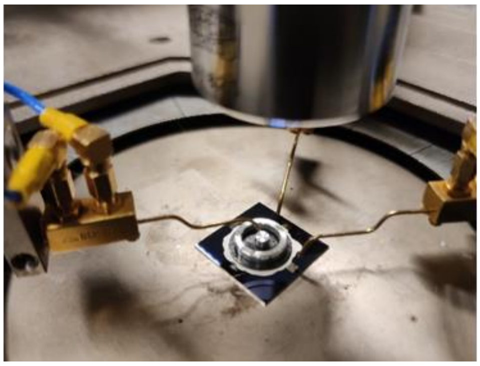
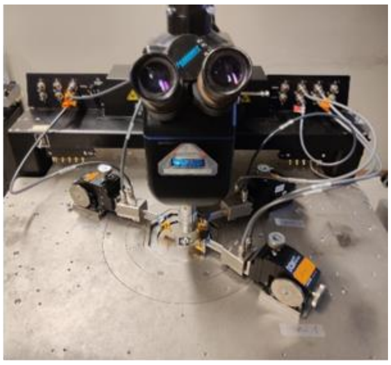

X-Ray photoelectron spectroscopy (XPS): XPS measurements were carried out on an Axis Supra (Kratos Analytical) using the monochromated Kα X-ray line of an aluminum anode. The pass energy was set to 40 eV with a step size of 0.15 eV. The samples were electrically insulated and charge neutralization - using a low energy electron flood gun was used. Adequate energy referencing and data analysis were performed using CasaXPS.

Ultraviolet photoelectron spectroscopy (UPS): UPS measurements were carried out on an Axis Supra (Kratos Analytical) using 21.22 eV photons emitted by an He I UV source. The pass energy was set to 10 eV with a step size of 0.025 eV. The samples were electrically grounded to limit charging effects.

Photoluminescence (PL) spectroscopy: PL spectra (450–700 cm−1) of the material deposited on a glass substrate were recorded on an Agilent Cary Eclipse Fluorescence Spectrometer.

X-Ray absorption spectroscopy (XAS): XAS measurements were conducted at the BM31 / Swiss Norwegian Beamlines (SNBL) at the European Synchrotron Radiation Facility (ESRF) in Grenoble, France. The polychromatic beam from the 2-pole wiggler (2PW) was monochromatized using a Si(111) LN_2-cooled monochromator, and the parallel beam was defined by the use of slits to a size of 3 mm (horizontal) × 300 μm (vertical).

Data were acquired from pressed pellets (1.3 cm^2) properly diluted with an inert substance (i.e., cellulose) and an appropriate thickness (typically of 2-3 absorption lengths) at the respective metal edge in transmission mode, using two ionization

chambers placed before and after the pellet and optimized to 20% absorption in the monitor (I_0) and 80% absorption in the transmitted chamber (I_1). XAFSmass was employed to perform the corresponding calculations and extract the optimal mass of materials.[7] An absorption step around 1 was always obtained. For the absolute energy calibration, a metal foil was measured simultaneously between the second and third ionization chambers. All 3 cm long ionization chambers were filled with inert gases and optimized to the numbers mentioned above. Typically, a scan was performed using around 0.5 eV step-size and 100 ms integration time per point resulting in a total scan time of ca. 4 min (minimum 20 repetitions for improved statistics, better S/N, etc.). Data reduction protocols, i.e., background removal, normalization and averaging (more scans were averaged for better S/N), truncation/deglitching, etc. were performed using Athena software from the Demeter package.[8] The corresponding fittings for the monomer samples were performed using Artemis software from the same package.

Computational details: All the quantum chemistry DFT calculations were performed using the ORCA quantum chemistry package 5.0.2.[9] Geometry optimizations of the metal monomers were performed at PBE0/D3BJ level of theory using the def2-TZVP basis set.[10,11] All the optimized geometries were verified as minima by the absence of imaginary frequencies. EPR and ZFS calculations were performed using the included ORCA package at the same level of theory. The EPR spectra were simulated using the EasySpin 6.0 suite.

Synthesis and characterization

Synthesis of 9,11,20,22-tetraazatetrapyrido [3,2-*a*:2‘,3‘-*c*:3‘ ‘,2‘ ‘-l:2‘ “,3‘ “-*n*]pentacene (tatppb)

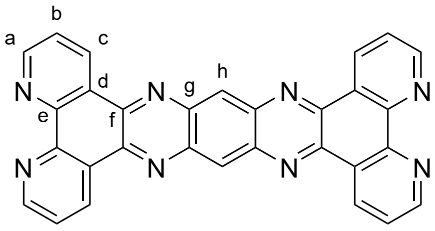

1,10-phenanthroline-5,6-dione (500 mg, 2.40 mmol, 2.00 eq.) and 1,2,4,5-benzenetetramine tetrahydrochloride (338 mg, 1.20 mmol, 1 eq) were suspended in degassed acetic acid (10 mL) and ethanol (3 mL). The mixture was stirred for 30 minutes at 100°C. Next, Et_3N (1.2 mL, 8.6 mmol, 7.2 eq.) was added and the mixture was further stirred at 115°C for 24 hours. The suspension was poured into a mixture of ice and water and the solid was filtered, washed with water, methanol, DCM and Et_2O and dried overnight under vacuum at 70°C to obtain the product as a magenta powder (432 mg, 74%). ^1H NMR (400 MHz, $CDCl_3$:CF_3COOD, 9:1, ppm) δ = 10.19 (dd, *J* = 8.2, 1.5 Hz, 4H, H_a), 9.81 (s, 2H, H_h), 9.35 (dd, *J* = 5.1, 1.6 Hz, 4H, H_b), 8.37 (dd, *J* = 8.2, 5.1 Hz, 4H, H_c). ^{13}C NMR (101

MHz, $CDCl_3$:CF_3COOD, 9:1) δ = 152.03 (C_f), 149.43 (C_c), 141.75 (C_e), 140.97 (C_g), 140.31 (C_a), 131.82 (C_h), 129.86 (C_d), 128.07 (C_b). HRMS (nanochip-ESI/LTQ-Orbitrap) m/z: $[M + H]^+$ Calcd for $C_{30}H_{15}N_8^+$ 487.1414; Found 487.1426. IR (neat, cm^{-1}): $\tilde{\nu}$ = 3070–2925 (w, $\nu_{(as)C–H}$ + $\nu_{(s)C–H}$), 1618 (w, $\nu_{(as)C=N(Phen)}$ + $\nu_{(as)C=C(Phen)}$), 1581 (m, $\nu_{(s)C=N(Phen)}$ + $\nu_{(s)C=C(Phen)}$), 1560 (w, $\nu_{(as)C=N(tetraazaacene)}$ + $\nu_{(as)C=C(tetraazaacene)}$), 1501 (m br, $\nu_{(s)C=N(tetraazaacene)}$ + $\nu_{(s)C=C(tetraazaacene)}$), 1463 (m, $\nu_{(as)C=C(Phen)}$), 1404 (s, $\nu_{(s)C=C(Phen)}$), 1348 (s, ν $\nu_{(s)C=N(tetraazaacene)}$ + $\nu_{(s)C=C(tetraazaacene)}$), 1320 (w, $\nu_{(s)C=C(tetraazaacene)}$ + $\nu_{(s)C=N(tetraazaacene)}$), 1129 (w br, $\delta_{(s)C=C}$ + $\rho_{C–H}$), 1061 (m, $\delta_{(s)C=C}$ + $\delta_{(s)C–H}$ + $\rho_{C–H}$), 1020 (s, $\nu_{C–N}$ + $\delta_{(s)C=N}$ + $\delta_{(s)C–H}$ + $\rho_{C–H}$), 878 (m, $\omega_{C–H(tetraazaacene)}$), 817 (m br, $\omega_{C–H(Phen)}$), 741 (vs, $\omega_{C–H(Phen)}$).

Synthesis of the metal monomers

Mn^{II}(1,10-Phenanthroline-5,6-dione)$_2$$Cl_2$ (**Mn-M**)

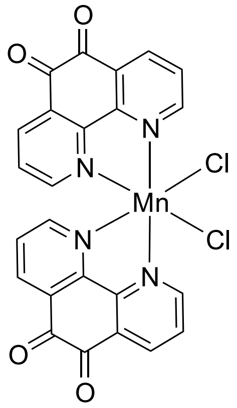

To solution of $MnCl_2 \cdot 4H_2O$ (0.69 g, 3.5 mmol, 1.0 eq.) in ethanol (250 mL), 1,10-Phenanthroline-5,6-dione (1.47 g, 7.0 mmol, 2.0 eq.) was added. The mixture was stirred for 2 hours at room temperature and the product was precipitated by addition of Et_2O. The solid was collected by filtration, washed with DCM, acetone and Et_2O and dried overnight under vacuum at 70°C to obtain the pure product as a pale-yellow powder (1.80 g, 93%). ^1H NMR (400 MHz, d_6-DMSO, ppm): δ = 9.01 (bs, 4H), 8.38 (bs, 4H), 7.67 (bs, 4H). HRMS (Nanochip-based ESI/LTQ-Orbitrap) m/z: $[M - Cl^-]^+$ Calcd for $C_{24}H_{12}ClMnN_4O_4^+$ 509.9922; Found 509.9922. IR (neat, cm^{-1}): $\tilde{\nu}$ = 1694 (s, $\nu_{C=O}$). UV-Vis (DMSO, nm): λ_{max} = 251, 292, 304, 358. Anal. Calcd for $C_{24}H_{12}Cl_2N_4O_4Mn$: C, 52.77; H, 2.21; N, 10.26. Found: C, 52.21; H, 2.33; N, 10.09. Single crystals suitable for scXRD were grown by cooling down a concentrated solution of **Mn-M** in boiling water.

$[Fe^{II}(1,10\text{-Phenanthroline-5,6-dione})_3]SO_4$ (**Fe-M**)

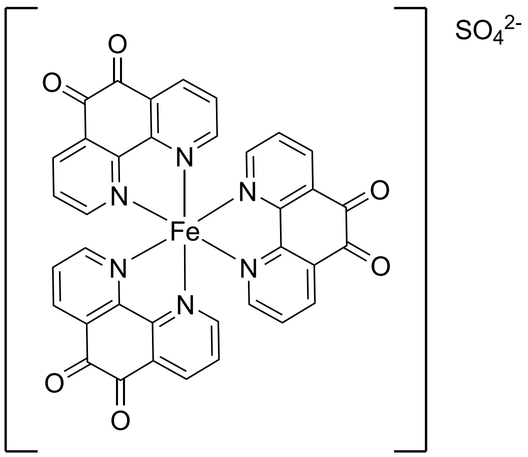

To a solution of $FeSO_4 \cdot 6H_2O$ (0.97 g, 3.5 mmol, 1.0 eq.) in ethanol (250 mL), 1,10-phenanthroline-5,6-dione (2.2 g, 10.5 mmol, 3.0 eq.) was added. The mixture was stirred for 24 hours at room temperature and the product was precipitated by addition of Et_2O. The solid was collected by filtration, washed with DCM, acetone and Et_2O and dried overnight under vacuum at 70°C to obtain the desired product as a dark red powder (2.0 g, 73%). The product can be recrystallized from acetonitrile and Et_2O. ^1H NMR (400 MHz, d_6-DMSO, ppm): δ = 8.99 (s, 2H), 8.52 (d, J = 98.8 Hz, 8H), 7.60 (d, J = 58.9 Hz, 8H). HRMS (Nanochip-based ESI/LTQ-Orbitrap) m/z: $[M - SO_4^{-}]^{+2}$ Calcd for $C_{36}H_{18}FeN_6O_6^{+2}$ 343.0313; Found 343.0312. IR (neat, cm^{-1}): $\tilde{\nu}$ = 1697 (s, $\nu_{C=O}$). UV-Vis (DMSO, nm): λ_{max} = 252, 298, 364, 421.

$[Co^{II}(1,10\text{-Phenanthroline-5,6-dione})_3]Cl_2$ (**Co-M**)[12]

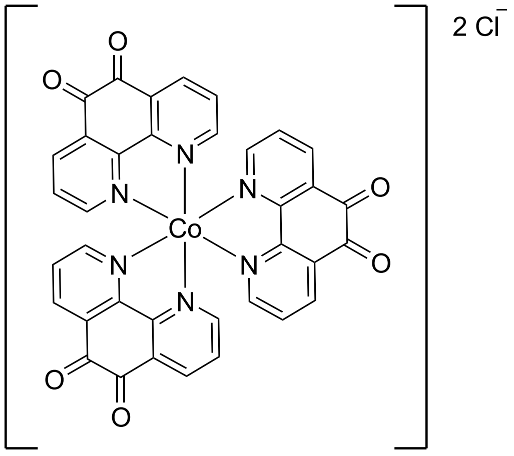

To a solution of anhydrous $CoCl_2$ (0.45 g, 3.5 mmol, 1.0 eq.) in ethanol (250 mL), 1,10-phenanthroline-5,6-dione (2.6 g, 12.25 mmol, 3.5 eq.) was added. The mixture was stirred for 4 hours at room temperature and the product was precipitated by addition of Et_2O. The solid was collected by filtration, washed with DCM, acetone and Et_2O and dried overnight under vacuum at 70°C to obtain the desired product as an orange powder (1.6 g, 60%). The product can be recrystallized from hot ethanol. ^1H NMR (400 MHz, d_6-DMSO, ppm): δ = 8.94 (d, J = 4.6 Hz, 6H), 8.33 (d, J = 7.8 Hz, 6H), 7.68 – 7.60 (m, 6H). HRMS (nanochip-ESI/LTQ-Orbitrap) m/z: $[M - 2Cl^{-}]^{+2}$ Calcd for $C_{36}H_{18}CoN_6O_6^{+2}$ 344.5304; Found 344.5293. IR (neat, cm^{-1}): $\tilde{\nu}$ = 1694 (s, $\nu_{C=O}$). UV-Vis (DMSO, nm): λ_{max} = 249, 298, 305, 363. Anal. Calcd for $C_{36}H_{18}N_6O_6Co \cdot Cl_2 \cdot 4(H_2O)$: C, 51.94; H, 3.15; N, 10.10. Found: C, 51.37; H, 2.37; N, 9.96.

[Ni^{II}(1,10-Phenanthroline-5,6-dione)$_3$](PF$_6$)$_2$ (**Ni-M**)[13]

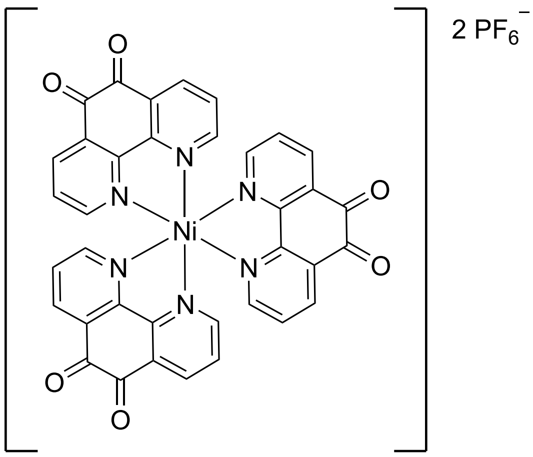

Ni(OAc)$_2$ · 4H$_2$O (1.307 g, 5.25 mmol, 1 eq.), 1,10-phenanthroline-5,6-dione (3.31 g, 15.7 mmol, 3 eq.) and NH$_4$PF$_6$ (1.71 g, 10.5 mmol, 2 eq.) were suspended in methanol (240 mL). The resulting orange-brown slurry was stirred vigorously for 7 hours at room temperature and the obtained suspension was filtered, washed with methanol (80 mL), water (2 x 250 mL) and methanol (2x60 mL) and then dried at 60 ºC overnight to afford a tan product (3.31 g, 64%). ^{19}F NMR (376 MHz, d_6-DMSO, ppm): δ = −69.97 (d, J = 712.2 Hz). ^{31}P NMR (162 MHz, d_6-DMSO, ppm): δ = −141.98 (hept, J = 710.6 Hz). HRMS (nanochip-ESI/LTQ-Orbitrap) m/z: [M – 2PF$_6^-$]$^{+2}$ Calcd for $C_{36}H_{18}N_6NiO_6^{+2}$ 344.0315; Found 344.0312. IR (neat, cm^{-1}): $\tilde{v}$ = 1697 (s, $v_{C=O}$). Anal. Calcd for $C_{36}H_{18}N_6O_6Ni$ · (PF$_6$)$_2$ · 2(H$_2$O): C, 42.59; H, 2.18; N, 8.28. Found: C, 42.90; H, 2.30; N, 8.37.

Cu^{II}(1,10-Phenanthroline-5,6-dione)$_2$Cl$_2$ (**Cu-M**)

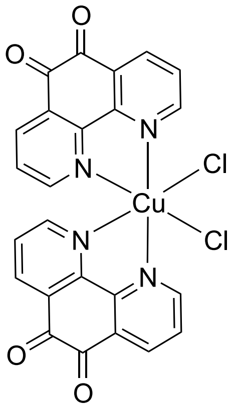

To a solution of CuCl$_2$ · 2H$_2$O (0.60 g, 3.5 mmol, 1.0 eq.) in ethanol (250 mL), 1,10-phenanthroline-5,6-dione (1.47 g, 7.0 mmol, 2.0 eq.) was added. The mixture was stirred for 1 hour at room temperature and the product was precipitated by addition of Et$_2$O. The solid was collected by filtration, washed with DCM, acetone and Et$_2$O and dried overnight under vacuum at 70°C to obtain the desired product as a green powder (1.48 g, 76%). HRMS (nanochip-ESI/LTQ-Orbitrap) m/z: [M – Cl$^-$]$^+$ Calcd for $C_{24}H_{12}ClCuN_4O_4^+$ 517.9838; Found 517.9839. IR (neat, cm^{-1}): $\tilde{v}$ = 1694 (s, $v_{C=O}$). UV-Vis (DMSO, nm): λ_{max} = 304, 363. Anal. Calcd for $C_{24}H_{12}N_4O_4CuCl_2$ · H$_2$O: C, 50.32; H, 2.46; N, 9.78. Found: C, 51.09; H, 2.17; N, 9.79 Single crystals suitable for scXRD were grown by cooling down a concentrated solution of **Cu-M** in water.

Zn^{II}(1,10-Phenanthroline-5,6-dione)$_2$Cl$_2$ (**Zn-M**)

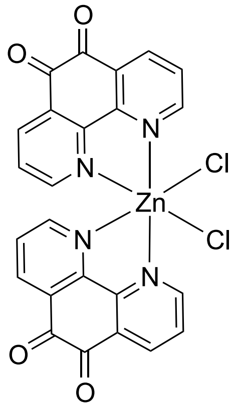

To a solution of $ZnCl_2$ (0.48 g, 3.5 mmol, 1.0 eq.) in ethanol (250 mL), 1,10-phenanthroline-5,6-dione (1.5 g, 7.0 mmol, 2.0 eq.) was added. The mixture was stirred for 24 hours at room temperature and the product was precipitated by addition of Et_2O. The solid was collected by filtration, washed with DCM, acetone and Et_2O and dried overnight under vacuum at 70°C to obtain the desired product as a light-yellow powder (1.79 g, 92%). ^{1}H NMR (400 MHz, d_6-DMSO, ppm): δ = 8.88 (s, 4H), 8.54 (d, J = 8.1 Hz, 4H), 7.82 (dd, J = 7.9, 4.9 Hz, 4H). ^{13}C NMR (101 MHz, d_6-DMSO, ppm): δ = 176.38, 152.78, 149.02, 137.28, 129.95, 126.90. HRMS (nanochip-ESI/LTQ-Orbitrap) m/z: [M – Cl$^-$]$^+$ Calcd for $C_{24}H_{12}ClN_4O_4Zn^+$ 518.9833; Found 518.9823. IR (neat, cm^{-1}): $\tilde{\nu}$ = 1694 (s, $\nu_{C=O}$). UV-Vis (DMSO, nm): λ_{max} = 252, 297, 307, 359, 448. Anal. Calcd for $C_{24}H_{12}N_4O_4ZnCl_2 \cdot H_2O$: C, 50.16; H, 2.46; N, 9.75. Found: C, 50.85; H, 2.24; N, 9.73. Single crystals suitable for scXRD were grown from a concentrated solution of **Zn-M** in water.

Ru^{II}(1,10-Phenanthroline-5,6-dione)$_2$Cl$_2$ (**Ru-M**)[14]

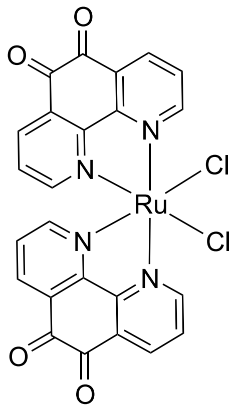

To a solution of Ru(DMSO)$_4$Cl$_2$ (1.45 g, 3.0 mmol, 1 eq.) in DMF (100 mL), 1,10-phenanthroline-5,6-dione (1.3 g, 6.0 mmol, 2 eq.) was added. The mixture was stirred for 72 hours at 120°C. After cooling down, acetone (1L) was added and the mixture was left at −20°C overnight. The solid was collected by filtration, washed with acetone, methanol, and Et_2O and dried overnight under vacuum at 70°C to obtain the desired product as a black powder (2.77 g, 78%). ^{1}H NMR (400 MHz, d_6-DMSO, ppm): δ = 10.12 (dd, J = 5.7, 1.5 Hz, 2H), 8.48 (dd, J = 7.8, 1.5 Hz, 2H), 8.10 (dd, J = 7.8, 1.3 Hz, 2H), 8.01 (dd, J = 7.8, 5.7 Hz, 2H), 7.77 (dd, J = 5.8, 1.4 Hz, 2H), 7.35 (dd, J = 7.8, 5.7 Hz, 2H). ^{13}C NMR (101 MHz, d_6-DMSO, ppm): δ = 174.87, 174.69, 159.10, 157.43, 156.73, 156.00, 132.53, 131.41, 130.68, 130.55, 126.48, 126.17. IR (neat, cm^{-1}): $\tilde{\nu}$ = 1695 (s, $\nu_{C=O}$). HRMS (Nanochip-based ESI/LTQ-Orbitrap) m/z: [M – 2Cl$^-$]$^{+2}$ Calcd for $C_{36}H_{18}N_6O_6Ru^{+2}$ 366.0160; Found 366.0157. UV-Vis (DMSO, nm): λ_{max} = 287, 334. Anal. Calcd for $C_{24}H_{12}N_4O_4Cl_2Ru \cdot H_2O$: C, 47.23; H, 2.31; N, 9.18. Found: C, 47.99; H, 2.50; N, 9.34.

[M^{III}(1,10-Phenanthroline-5,6-dione)$_2$Cl]Cl_2 (M = Rh or Ir, **Rh-P** or **Ir-P**)

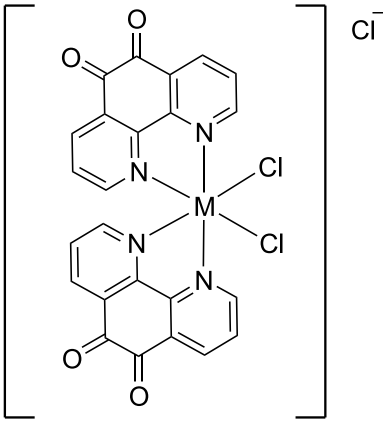

A solution of MCl_3 hydrate (1.75 mmol) and 1,10-phenanthroline-5,6-dione (0.75 g, 3.5 mmol, 2 eq.) in acetonitrile (50 mL) was refluxed at 90°C for 12 hours. The suspension was filtered and the solid washed with DCM and Et_2O and dried overnight under vacuum at 70°C C to obtain the desired product.

[Rh^{III}(1,10-Phenanthroline-5,6-dione)$_2$Cl]Cl_2 (**Rh-M**): Yellow powder (0.58 g, 45%). ^{1}H NMR (400 MHz, DMSO-d_6, ppm): δ = 9.84 (dd, *J* = 5.7, 1.1 Hz, 1H), 9.03 (dd, *J* = 4.9, 1.7 Hz, 1H), 8.58 (dd, *J* = 7.9, 1.5 Hz, 1H), 8.53 (dd, *J* = 7.9, 1.8 Hz, 1H), 8.00 (dd, *J* = 7.9, 5.7 Hz, 1H), 7.81 (dd, *J* = 7.9, 4.7 Hz, 1H). ^{13}C NMR (101 MHz, DMSO-d_6, ppm): δ = 176.68, 173.24, 156.38, 154.86, 153.14, 150.17, 136.97, 136.09, 130.83, 129.61, 127.05, 126.24. IR (neat, cm^{-1}): $\tilde{v}$ = 1702 (s, $v_{C=O}$). UV-Vis (DMSO, nm): λ_{max} = 253, 291, 317, 363.

[Ir^{III}(1,10-Phenanthroline-5,6-dione)$_2$Cl]Cl_2 (**Ir-M**): Red powder (0.71 g, 64%). ^{1}H NMR (400 MHz, d_6-DMSO, ppm): δ = 9.83 (d, *J* = 5.7 Hz, 1H), 9.05 (dd, *J* = 4.9, 1.7 Hz, 1H), 8.57 (dd, *J* = 7.9, 1.7 Hz, 1H), 8.39 (d, *J* = 7.8 Hz, 1H), 7.93 (dd, *J* = 7.9, 5.8 Hz, 1H), 7.85 (dd, *J* = 7.9, 4.8 Hz, 1H). ^{13}C NMR (101 MHz, d_6-DMSO, ppm): δ = 176.42, 173.08, 159.75, 154.29, 152.85, 149.64, 137.30, 135.27, 130.67, 129.73, 126.99, 126.50. HRMS (Nanochip-based ESI/LTQ-Orbitrap) m/z: [M]$^+$ Calcd for $C_{24}H_{12}Cl_2IrN_4O_4^+$ 682.9859; Found 682.9846. IR (neat, cm^{-1}): $\tilde{v}$ = 1701 (s, $v_{C=O}$). UV-Vis (DMSO, nm): λ_{max} = 250, 290, 325, 360. Anal. Calcd for $C_{24}H_{24}Cl_3IrN_4O_{10}$: C, 34.85; H, 2.92; N, 6.77. Found: C, 34.80; H, 1.90; N, 6.65.

[Pd^{II}(1,10-Phenanthroline-5,6-dione)$_2$]$(PF_6)_2$ (**Pd-M**)

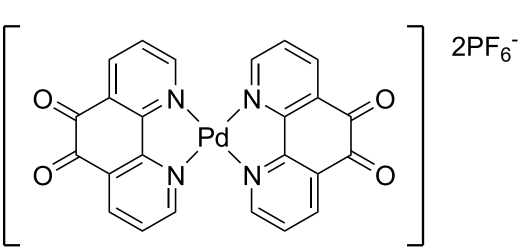

To a solution of $Pd(NO_3)_2$ (0.40 g, 1.75 mmol, 1 eq.) in acetone (250 mL), 1,10-phenanthroline-5,6-dione (0.74 g, 3.5 mmol, 2 eq.). The mixture was stirred for 72 hours at room temperature was added. The yellow solid was collected by filtration and redissolved in methanol (500 mL). NH_4PF_6 (1.43 g, 8.75 mmol, 5 eq.) was added and the mixture was stirred for 48 hours at room temperature. After the addition of Et_2O, solid was collected by filtration, washed with acetone, DDM, and Et_2O and dried overnight under vacuum at 70°C to obtain the desired product as a light brown powder (1.04 g, 76%). The product can be recrystallized from hot acetonitrile. ^{1}H NMR (400 MHz, d_6-DMSO,

ppm): δ = 9.05 (dd, J = 5.7, 1.4 Hz, 1H), 8.90 (dd, J = 8.0, 1.3 Hz, 1H), 8.11 (dd, J = 8.0, 5.7 Hz, 1H). ^{13}C NMR (101 MHz, d_6-DMSO, ppm): δ = 172.68, 154.49, 153.92, 139.16, 131.16, 129.16. ^{19}F NMR (376 MHz, d_6-DMSO, ppm): δ = 70.08 (d, J = 711.8 Hz). ^{31}P NMR (162 MHz, d_6-DMSO, ppm): δ = 144.20 (hept, J = 711.3 Hz). HRMS (Nanochip-based ESI/LTQ-Orbitrap) m/z: [M – $2PF_6^-$]$^{+2}$ Calcd for $C_{24}H_{12}N_4O_4Pd^{+2}$ 262.9941; Found 262.9944. IR (neat, cm^{-1}): $\tilde{v}$ = 1703 (s, $\nu_{C=O}$). UV-Vis (DMSO, nm): λ_{max} = 252, 284, 318, 363. Anal. Calcd for $C_{24}H_{12}N_4O_4Pd \cdot (PF_6)_2$: C, 35.29; H, 1.48; N, 6.86. Found: C, 35.74; H, 1.70; N, 6.78.

[Pt^{II}(1,10-Phenanthroline-5,6-dione)$_2$]$(PF_6)_2$ (**Pt-M**)

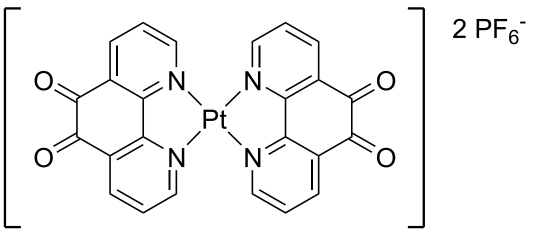

To a solution of $Pt(DMSO)_2Cl_2$ (0.76 g. 1.8 mmol 1 eq.) in methanol (100 mL), 1,10-phenanthroline-5,6-dione (0.38 g. 1.8 mmol 1 eq.) was added. The mixture was refluxed for 3 hours and, after cooling down, the yellow precipitate, Pt(1,10-phenanthroline-5,6-dione)Cl_2, was filtered, washed with methanol, DCM and Et_2O and dried (0.81 g, 94 %). Pt(1,10-phenanthroline-5,6-dione)Cl_2 (0.74 g, 1.5 mmol, 1 eq.) and 1,10-phenanthroline (0.64 g, 3.0 mmol, 2 eq.) were loaded into a 20 mL microwave glass vessel. Degassed ethylene glycol (4 mL) and water (8 mL) were added and the mixture was heated at 180°C for 6 hours in a microwave reactor. Afterwards, NH_4PF_6 (1.22 g , 7.5 mmol, 5 eq.) was added and the mixture was stirred for 1 hour at room temperature. The suspension was filtered and the solid was washed with methanol and DCM. The solid was extracted with hot acetonitrile and the filtrate was concentrated under reduced pressure. The obtained solid was dried overnight under vacuum at 70°C to obtain the desired product as a dark brown powder (0.73 g, 52%). ^{1}H NMR (400 MHz, d_6-DMSO, ppm): δ = 8.99 (dd, J = 4.7, 1.8 Hz, 4H), 8.42 (dd, J = 7.8, 1.8 Hz, 4H), 7.70 (dd, J = 7.8, 4.7 Hz, 4H). ^{13}C NMR (101 MHz, d_6-DMSO, ppm): δ = 177.48, 154.03, 151.76, 135.99, 129.22, 125.47. ^{19}F NMR (376 MHz, d_6-DMSO, ppm): δ = −70.13 (d, J = 711.0 Hz). ^{31}P NMR (162 MHz, d_6-DMSO, ppm): δ = −144.19 (hept, J = 711.9 Hz). MALDI (LDI/TOF) m/z: [M – $2PF_6^-$]$^+$ Calcd for $C_{24}H_{12}N_4O_4Pt^+$ 615; Found 615. IR (neat, cm^{-1}): $\tilde{v}$ = 1704 (s, $\nu_{C=O}$). UV-Vis (DMSO, nm): λ_{max} = 242, 294. Anal. Calcd for $C_{24}H_{12}F_{12}N_4O_4P_2Pt$: C, 31.84; H, 1.34; N, 6.19. Found: C, 28.92; H, 1.41; N, 5.49. Anal. Calcd for $C_{24}H_{12}N_4O_4Pd \cdot (PF_6)_2 \cdot (H_2O)_4$: C, 29.49; H, 2.06; N, 5.73. Found: C, 28.92; H, 1.41; N, 5.49.

Automated synthesis of Mn-M, Co-M, Cu-M and Zn-M

The automated monomer synthesis was performed in a SWING XL SP reactor (Chemspeed technologies AG). Reactions were performed in triplicates. Stock solutions of the different metal salts (10 mM in EtOH) and of 1,10-phenanthroline-5,6-dione (20 mM in DCM) were loaded into MTP plate (24 x 4ml vials). 790.98 mg of $MnCl_2 \cdot 4H_2O$, 790.3 mg of $CoCl_2$, 790.7 mg of $CuCl_2 \cdot 2H_2O$, or 790.3 mg of $ZnCl_2$ of the respective stock solution were gravimetrically dispensed into the reaction vials using the Gravimetric Dispense Unit for volume fine dosing (GDU-V). Subsequently, stock solution of 1,10-phenanthroline-5,6-dione were added gravimetrically to the reaction vials (1189.81 mg for **Co-M**, and 793.20 mg for **Mn-M**, **Cu-M**, **Zn-M**). The final volume was 2.5 mL (for **Co-M**) or 2 mL (for **Mn-M**, **Cu-M**, **Zn-M**). The mixtures were then shaken at 250 rpm at room temperature for 3 hours. Following reaction, the suspension was allowed to settle for 1 hour and 2.5 mL of solution were aspirated using the 4-Needle Head tool. Then, the solid was washed with 0.7 mL of DCM, shook briefly, settled and then aspirated. This cycle was repeated five times. After the washing steps, the residual solvent was removed by heating to 45°C for 30 minutes. The final products were collected and analyzed.

Mn-M. 90 ± 4% (4.9 ± 0.3 mg). IR (neat, cm^{-1}): $\tilde{v}$ = 1694 (s, $v_{C=O}$). HRMS (Nanochip-based ESI/LTQ-Orbitrap) m/z: $[M - 2Cl^{-}]^{+2}$ Calcd for $C_{24}H_{12}MnN_4O_4^{+2}$ 237.5114; Found 237.5118.

Co-M. 49 ± 1% (3.7 ± 0.1 mg). IR (neat, cm^{-1}): $\tilde{v}$ = 1694 (s, $v_{C=O}$). HRMS (nanochip-ESI/LTQ-Orbitrap) m/z: $[M - 2Cl^{-}]^{+2}$ Calcd for $C_{36}H_{18}CoN_6O_6^{+2}$ 344.5304; Found 344.5311.

Cu-M. 22 ± 2% (1.2 ± 0.1 mg). IR (neat, cm^{-1}): $\tilde{v}$ = 1690 (s, $v_{C=O}$). HRMS (nanochip-ESI/LTQ-Orbitrap) m/z: $[M - 2Cl^{-}]^{+2}$ Calcd for $C_{24}H_{12}CuN_4O_4^{+}$ 483.0149; Found 483.0138.

Zn-M. 94 ± 5% (4.6 ± 0.3 mg). ^{1}H NMR (400 MHz, d_6-DMSO, ppm): δ = 8.88 (s, 4H), 8.55 (d, J = 7.8 Hz, 4H), 7.82 (dd, J = 7.9, 4.9 Hz, 4H). ^{13}C NMR (101 MHz, d_6-DMSO, ppm): δ = 176.42, 152.89, 137.04, 129.81, 126.66. IR (neat, cm^{-1}): $\tilde{v}$ = 1694 (s, $v_{C=O}$). HRMS (nanochip-ESI/LTQ-Orbitrap) m/z: $[M - Cl^{-}]^{+}$ Calcd for $C_{24}H_{12}ClN_4O_4Zn^{+}$ 518.9833; Found 518.9844.

HD-SAC polymer synthesis

The HD-SACs were prepared using 10 mL high precision glass microwave reaction vials from Biotage (part number. 351521) and closed using the corresponding aluminum cap with a septum. The reaction was placed in a custom-made aluminum

heating block and heated using an IKA RCT standard heating plate equipped with a thermocouple. No differences were observed when using an oil bath. The reaction was stirred at 1300 rpm using a 10 mm cross-shaped PTFE magnetic stirring bar.

Mn-P. 81.9 mg of **Mn-M** to obtain 62.1 mg of **Mn-P**. IR (neat, cm^{-1}): $\tilde{\nu}$ = 1573, 1510, 1380, 1350, 1299, 1131, 816, 736, 421. UV-Vis (DMSO, nm): λ_{max} = 250, 293. Volume electrical conductivity (S cm^{-1}): 7.9 ± 2.1 $\times 10^{-11}$. BET surface (N_2 sorption isotherm at 77 K, m^2 g^{-1}): 52.

Fe-P. 78.2 mg of **Fe-M** to obtain 72.8 mg of **Fe-P**. IR (neat, cm^{-1}): $\tilde{\nu}$ = 1576, 1516, 1372, 1319, 1122, 1072, 833, 718, 424. UV-Vis (DMSO, nm): λ_{max} = 252, 271, 295, 305, 363. Volume electrical conductivity (S cm^{-1}): 7.8 ± 1.7 $\times 10^{-11}$. BET surface (N_2 sorption isotherm at 77 K, $m^2 g^{-1}$): 63.

Co-P. 76.4 mg of **Co-M** to obtain 81.4 mg of **Co-P**. IR (neat, cm^{-1}): $\tilde{\nu}$ = 1575, 1534, 1383, 1308, 1127, 1067, 817, 734, 428, 417. UV-Vis (DMSO, nm): λ_{max} = 249, 300, 307, 363. Volume electrical conductivity (S cm^{-1}): 4.0 ± 1.0 $\times 10^{-10}$. BET surface (N_2 sorption isotherm at 77 K, m^2 g^{-1}): 34.

Ni-P. 97.9 mg of **Ni-M** to obtain 83.8 mg of **Ni-P**. IR (neat, cm^{-1}): $\tilde{\nu}$ = 1576, 1530, 1381, 1302, 1103, 818, 735, 425. UV-Vis (DMSO, nm): λ_{max} = 248, 298, 309, 363. BET surface (N_2 sorption isotherm at 77 K, m^2 g^{-1}): 23.

Cu-P. 83.2 mg of **Cu-M** to obtain 72.3 mg of **Cu-P**. IR (neat, cm^{-1}): $\tilde{\nu}$ = 1592, 1508, 1382, 1123, 1075, 815, 730, 428. UV-Vis (DMSO, nm): λ_{max} = 249, 299, 306, 362. BET surface (N_2 sorption isotherm at 77 K, m^2 g^{-1}): 22.

Zn-P. 83.5 mg of **Zn-M** to obtain 82.0 mg of **Zn-P**. IR (neat, cm^{-1}): $\tilde{\nu}$ = 1588, 1508, 1380, 1305, 1092, 817, 735, 422. UV-Vis (DMSO, nm): λ_{max} = 248, 298, 309, 363. BET surface (N_2 sorption isotherm at 77 K, m^2 g^{-1}): 35.

Ru-P. 88.9 mg of **Ru-M** to obtain 82.9 mg of **Ru-P**. IR (neat, cm^{-1}): $\tilde{\nu}$ = 1585, 1492, 1369, 1300, 1121, 1105, 1065, 812, 722, 464, 435. UV-Vis (DMSO, nm): λ_{max} = 273, 306, 312. Volume electrical conductivity (S cm^{-1}): 3.6 ± 0.9 $\times 10^{-10}$. BET surface (N_2 sorption isotherm at 77 K, m^2 g^{-1}): 55.

Rh-P. 94.4 mg of **Rh-M** to obtain 87.5 mg of **Rh-P**. IR (neat, cm^{-1}): $\tilde{\nu}$ = 1393, 1319, 1120, 1091, 1068, 812, 721, 440, 424. UV-Vis (DMSO, nm): λ_{max} = 222, 252, 260, 318, 411. Volume electrical conductivity (S cm^{-1}): 8.2 ± 1.9 $\times 10^{-11}$. BET surface (N_2 sorption isotherm at 77 K, m^2 g^{-1}): 13.

Pd-P. 122.5 mg of **Pd-M** to obtain 98.7 mg of **Pd-P**. IR (neat, cm^{-1}): $\tilde{\nu}$ = 1579, 1531, 1392, 1493, 1125, 1086, 809, 721, 436, 404. UV-Vis (DMSO, nm): λ_{max} = 249, 343, 351, 496, 579, 605. BET surface (N_2 sorption isotherm at 77 K, m^2 g^{-1}): 48.

Ir-P. 107.8 mg of **Ir-M** to obtain 95.4 mg of **Ir-P**. IR (neat, cm^{-1}): $\tilde{\nu}$ =1571, 1492, 1310, 1119, 1069, 1060, 810, 720, 441, 425. UV-Vis (DMSO, nm): λ_{max} = 250, 291, 325, 361. Volume electrical conductivity (S cm^{-1}): 8.2 ± 1.9 $\times 10^{-11}$. BET surface (N_2 sorption isotherm at 77 K, m^2 g^{-1}): 49.

Pt-P. 135.8 mg of **Pt-M** to obtain 101.3 mg of **Pt-P**. IR (neat, cm^{-1}): $\tilde{\nu}$ =1527, 1366, 1069, 732, 435. UV-Vis (DMSO, nm): λ_{max} = 241, 291, 316, 428, 453. Volume electrical conductivity (S cm^{-1}): 8.0 ± 1.8 $\times 10^{-10}$. BET surface (N_2 sorption isotherm at 77 K, m^2 g^{-1}): 19.

CoCu-P. 38.2 mg of **Co-M** and 41.6 mg of **Cu-M** to obtain 74.5 mg of **CoCu-P**.

CoRu-P. 38.2 mg of **Co-M** and 44.5 mg of **Ru-M** to obtain 76.5 mg of **CoRu-P**.

Automated polymer synthesis of Co-P

The automated polycondensation reaction was performed in a SWING XL Autoplant system (Chemspeed technologies AG) equipped with a 240 mL Process Development (PD) reactor fitted with a Teflon sleeve. **Co-M** and 1,2,4,5-benzenetetramine tetrahydrochloride were loaded to the PD reactor. Triethylamine was introduced into a 50 mL feed vessel connected to the PD reactor via a high-pressure pump, enabling the on-line addition of the reagent.

Co-M (0.76 g, 1 mmol, 0.67 eq.) and 1,2,4,5-benzenetetramine tetrahydrochloride (0.42 g, 1.5 mmol, 1 eq.) were gravimetrically dispensed into the PD reactor using a Gravimetric Dispense Unit for powder fine dosing (GDU-Pfd). Ethanol (9 mL) was then introduced volumetrically, followed by the addition of acetic acid (30 mL) using the 4-NeedleHead liquid dispenser, equipped with 2 x 1 mL syringes and 2 x 10 mL syringes. Methanol was used to rinse the syringes between each volumetric transfer of reagents, to avoid cross-contamination. The mixture was then heated to 100°C (temperature inside the PD reactor measured over time with a Pt100 thermocouple) and stirred at 800 rpm for 30 minutes. Triethylamine (1.5 mL, 10.8 mmol, 7.2 eq.) was injected from the feed vessel into the PD reactor. The solution was further heated to 115°C and stirred at 800 rpm for 72 hours. Upon completion of the reaction, the PD reactor was cooled to 30 °C and the stirring was stopped. The suspension was allowed to settle over 1 hour and 35 mL of the liquid phase was aspirated by the 4-NeedleHead and discarded.

To isolate **Co-P**, 120 mL of H_2O were added to the PD reactor via the 4-NeedleHead, and the mixture was stirred for 1 minute at 800 rpm, followed by a 1 hour resting time to allow the polymer to settle at the bottom of the reactor. Then, 70 mL of the solution in the PD reactor was aspirated and discarded volumetrically to the liquid

trash station. Subsequently, the polymer was rinsed with 70 mL of H_2O added to the PD reactor. The mixture was then stirred for 1 minute at 800 rpm, followed by a 1 hour resting time before the aspiration of 70 mL of the solution in the PD reactor and discarded to the liquid trash station. The rinsing cycle was repeated five times, followed by four rinsing cycles using 70 mL of methanol. 70 mL of the solution was aspired and discarded to the liquid trash station. A final filtration step with 200 ml MeOH was performed manually and the resulting solid was dried at 70 °C overnight to obtain **Co-P**.

Catalytic studies

Electrode preparation for CO_2 reduction reaction (CO2RR): Cathode catalyst ink was prepared by adding 15 mg of catalyst into a solution of 3 mL of EtOH and 50 µL of Nafion perfluorinated resin solution 1100W (Sigma-Aldrich). The mixture then underwent 1 hour of ultrasonication to obtain a homogeneously dispersed solution. The as-prepared ink was air-brushed onto vertically placed 3 cm x 3 cm AvCarb GDS3250 carbon paper on a heating plate at 70 °C. Finally, the electrode was cut into four pieces to give a 1.5 cm x 1.5 cm GDE.

Electrode preparation for hydrogen evolution reaction (HER): The cathode catalyst ink was prepared by adding 5 mg of catalyst into a solution of 900 µL IPA, 100 µL DI water, and 20 µL Nafion ionomer. The solution was ultrasonicated for 30 min in an ice bath to obtain a homogeneously dispersed solution. 20 µL of the as-prepared ink was drop-casted on a 0.07 cm^2 area GC electrode and left to dry in the air before each measurement.

Electrocatalytic CO2RR: A Gamry Reference 3000 electrochemical instrument was used to measure the electrochemical activity at ambient temperature and pressure. Potential was first measured with Ag/AgCl reference electrode (saturated KCl), which was then converted into potential versus reversible hydrogen electrode (RHE) with an automatic *i*R compensation of 85% using Equation 1. All potentials were reported against RHE unless indicated otherwise.

$$\mathrm{E_{RHE}} = \mathrm{E_{Ag/AgCl}} + 0.1976\ \mathrm{V} + 0.592 \times\ \mathrm{pH} + i\mathrm{R}$$

The measurement was carried out in a three-electrode custom-made gas diffusion electrode (GDE) flow cell with 1 cm x 1 cm windows. For sealing between each GDE and electrolyte chamber, silicon gasket with 1.5 cm x 1.5 cm window was used. Its cathodic and anodic chambers were filled with each 15 mL of aqueous 1 M potassium hydroxide (KOH). The counter electrode was NiFe/Ni foam synthesized according to a previous report.[15] FAA-3-PK-75 (Fumasep) anion exchange membrane was used

to separate the two chambers. Chronopotentiometry measurements were completed at −20, −50, −100 and −200 mA cm^{-2} for 1000 seconds for −20 mA cm^{-2} and 600 seconds for the rest. Three independent electrochemical measurements were performed to give an average and a standard deviation for an error bar. The gas inlet on the cathode side was connected to CO_2 flow. To quantify gas products, the outlet was connected to a gas chromatography (GC) (SRI 8610C) equipped with a thermal conductivity detector (TCD) and a flame ionization detector (FID). The product gas was injected into GC after 450 seconds of CO_2RR. The Fraradaic efficiency (FE) of the gaseous products was obtained from Equation 2 where A is the area in the chromatogram, *f* the flow rate, N_{Av} Avogadro-s constant, n_{e-} the number of electrons, *e* the elementary charge, *k* the calibration constant for each product, I_{tot} the total current and V_{mol} the molar volume.

$$FE = k \times A_{\text{product}} \frac{f\, N_{Av}\, n_{e^-}\, e}{I_{tot}\, V_{mol}}$$

The liquid products were quantified by ^{1}H NMR spectroscopy with 5 mM dimethyl sulfoxide (DMSO) solution as a standard. After the end of each measurement, 400 µL of electrolyte was mixed with 50 µL deuterium oxide and 40 µL standard solution. The FE was calculated using Equation 3 where I is the number of mol corresponding to a unit area, *C* the total charge, *F* the Faraday constant, n_H the number of hydrogens corresponding to a given NMR peak, V_{tot} the total volume of electrolyte during electrolysis and V_{elec} the volume of electrolyte used for the NMR spectra.

$$FE = k \times A_{\text{product}} \frac{V_{tot}\, n_{e^-}}{n_H\, V_{elec}\, F\, C}$$

For stability testing in a flow cell, the electrolyte was replaced with 0.5 M $KHCO_3$ (pH 8.4) to mitigate salt precipitation at the back of the cathode. Chronopotentiometry was performed at a current density of −200 mA cm^{-2} with a CO_2 flow rate of 30 sccm. Gaseous products were sampled every 15 min and quantified using GC, while liquid products were not analyzed.

Electrocatalytic HER: Before each measurement, a glassy carbon electrode was polished using diamond paste with particle sizes of 0.25 µm and 0.05 µm, followed by ultrasonication in isopropanol for 10 minutes. Electrochemical characterization of each catalyst was conducted in a three-electrode setup using linear sweep voltammetry (LSV) at a scan rate of 1 mV s^{-1} within the relevant potential windows. For HER, the potential range was 0 to -0.8 V vs. RHE. Catalyst activity was assessed in a nitrogen-saturated 1 M KOH solution, employing an Ag/AgCl (3 M KCl) reference electrode and a graphite counter electrode. The Ag/AgCl electrode was calibrated against the reversible hydrogen electrode (RHE) in a hydrogen-saturated 1 M KOH standard solution (pH = 14) at 23 °C, following the procedure[16].

$$E_{RHE} = \frac{E_{Ag}}{E_{AgCl}} + 1.016$$

IR drop correction (90%) was automatically applied by the Gamry Reference 3000 potentiostat. For HER stability testing in the three-electrode electrochemical cell configuration, a constant current density of −10 mA/cm² was maintained. No ink dissolution was observed in the electrolyte at the end of the measurement, confirming strong adhesion of the catalyst to the glassy carbon electrode. Further stability test at −200 mA/cm² was performed using the same experimental conditions.

Photocatalytic Hydrogen Evolution Reaction (HER): The photocatalytic HER was carried out in a 100 mL photocatalytic reactor. 5 mg of the catalyst was uniformly dispersed in 50 mL of deionized water without any external sacrificial agent. Argon was passed through the solution for 30 minutes to remove any dissolved gases from the solution. Prior to light exposure, the solution was ultrasonicated for 15 minutes to obtain a uniformly dispersed solution. The photocatalytic reactor was then irradiated with PR160L Kessil LED lamp (λ = 390 nm; 10 W) with continuous magnetic stirring for 2 hours The gaseous products were then analyzed using a headspace GC (Shimadzu Nexis, GC-2030, BID detector, Helium as carrier gas). For carrying out the long-term experiments, 5 mg of the catalyst was uniformly dispersed in 50 mL of deionized water without any external sacrificial agent. Argon f was passed through the solution for 30 minutes to remove any dissolved gases from the solution. Prior to light exposure, the solution was ultrasonicated for 15 minutes to ensure uniform suspension of the catalyst. The photocatalytic reactor was then irradiated with PR160L Kessil LED lamp (λ = 390 nm; 10 W) with continuous magnetic stirring for 3 hours. After an interval of every 1 hour, the gaseous products were analyzed using a headspace GC. Between subsequent cycles, the reaction was bubbled with Argon for 20 mins to remove all the dissolved gases before initiating the subsequent cycle under identical conditions. The catalyst exhibited high stability over five consecutive cycles, with no significant decline in activity observed.

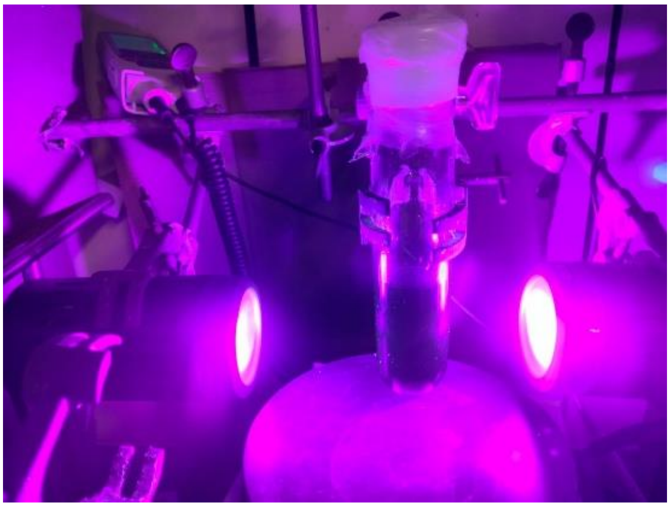

Supporting figures

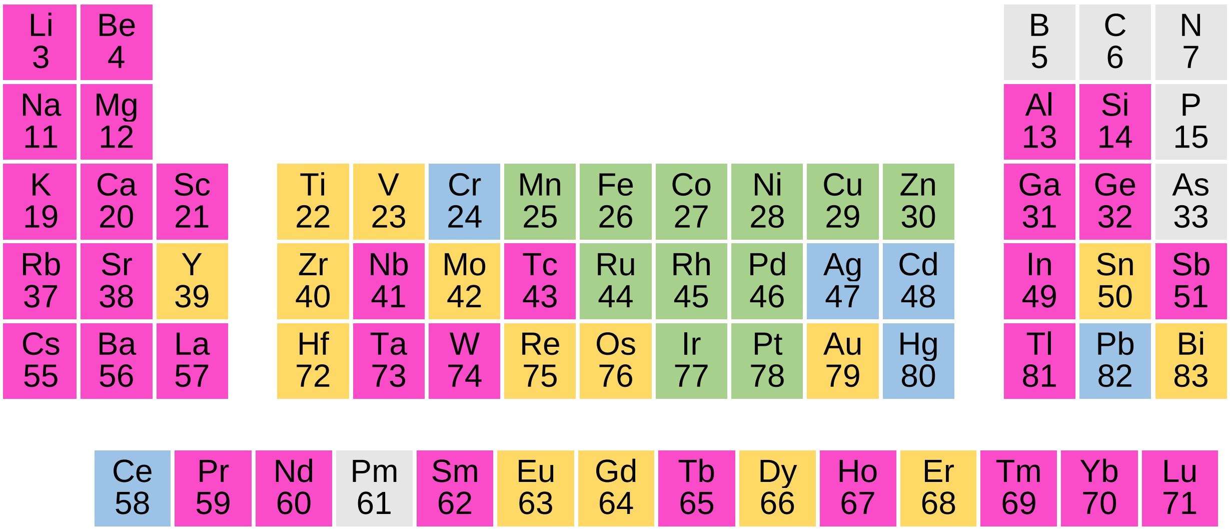

Figure S1 | Periodic table showing the potential scope of the developed HD-SAC synthesis. Metals used to produce HD-SAC polymers in this work (*green*) Metals with reported structures with two or more N-coordinated 1,10-phenanthroline-5,6-dione ligands (*blue*): Cr[17], Ag[18], Cd[19], Hg[20], Pb[21], Ce[22]. Potentially accessible metals with reported N-coordinated 1,10-phenanthroline-5,6-dione complexes and complexes with two or more 1,10-phenanthroline ligands (*yellow*): Ti[23], V[24], Y[25], Zr[23], Mo[26], Sn[27], Hf[23], Re[28], Os[29], Au[30], Bi[31], Eu[32], Gd[25], Dy[33], Er[32]. Potential metals for which compounds with two or more 1,10-phenanthroline ligands are reported (*pink*).

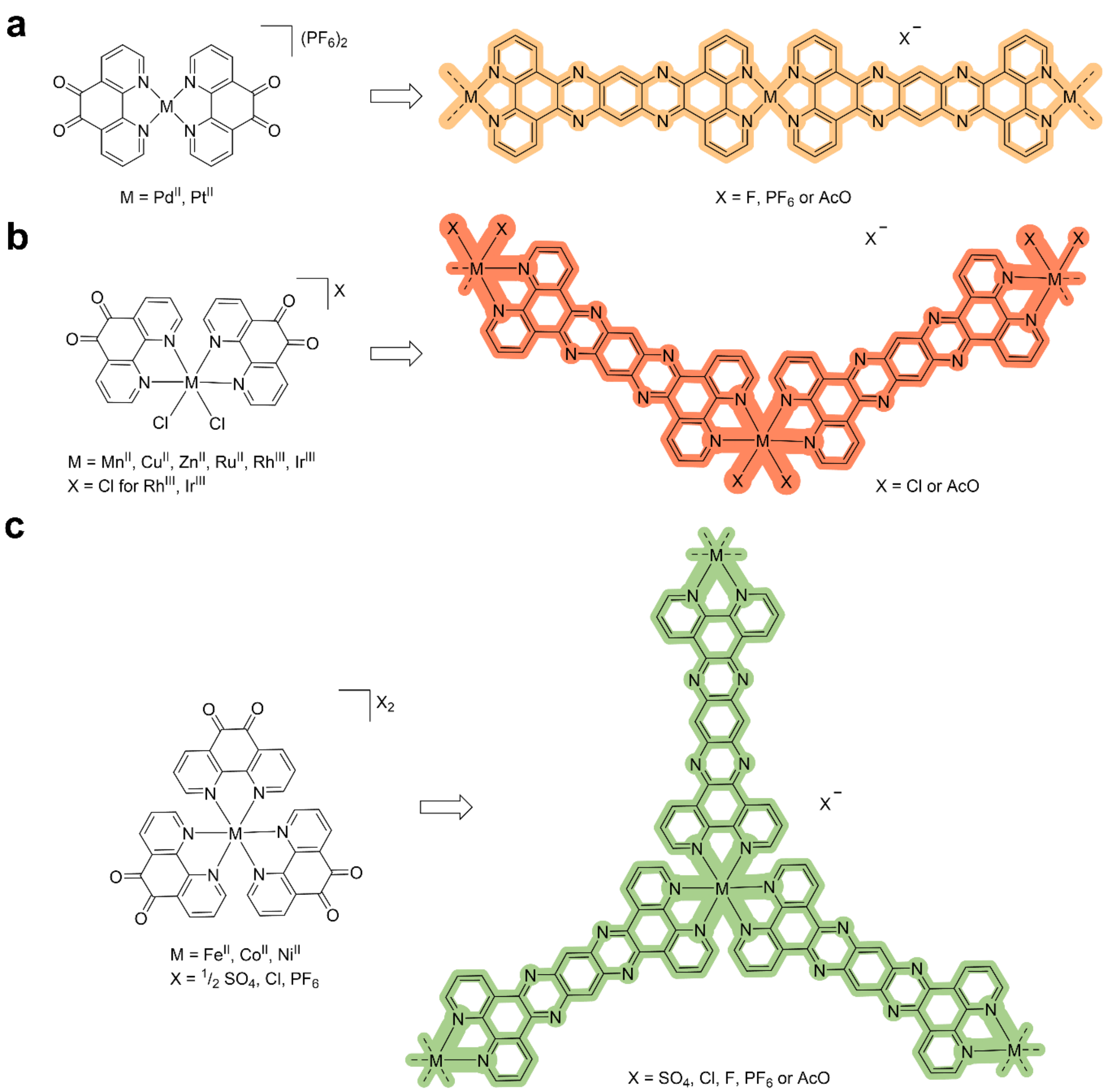

Figure S2 | HD-SAC architectures obtained depending on the coordination environment of the metal monomers. Quasi-planar linear HD-SACs from four-coordinate square planar metal monomers with two 1,10-phenanthroline-5,6-dione ligands (**a**), bent linear HD-SACs from six-coordinate octahedral complexes with two 1,10-phenanthroline-5,6-dione ligands and two chloride ligands (**b**), and HD-SACs expanding in three directions from six-coordinate octahedral complexes with three 1,10-phenanthroline-5,6-dione ligands (**c**). The bound chlorides and counterions are partially exchanged for acetates during the polymerization.

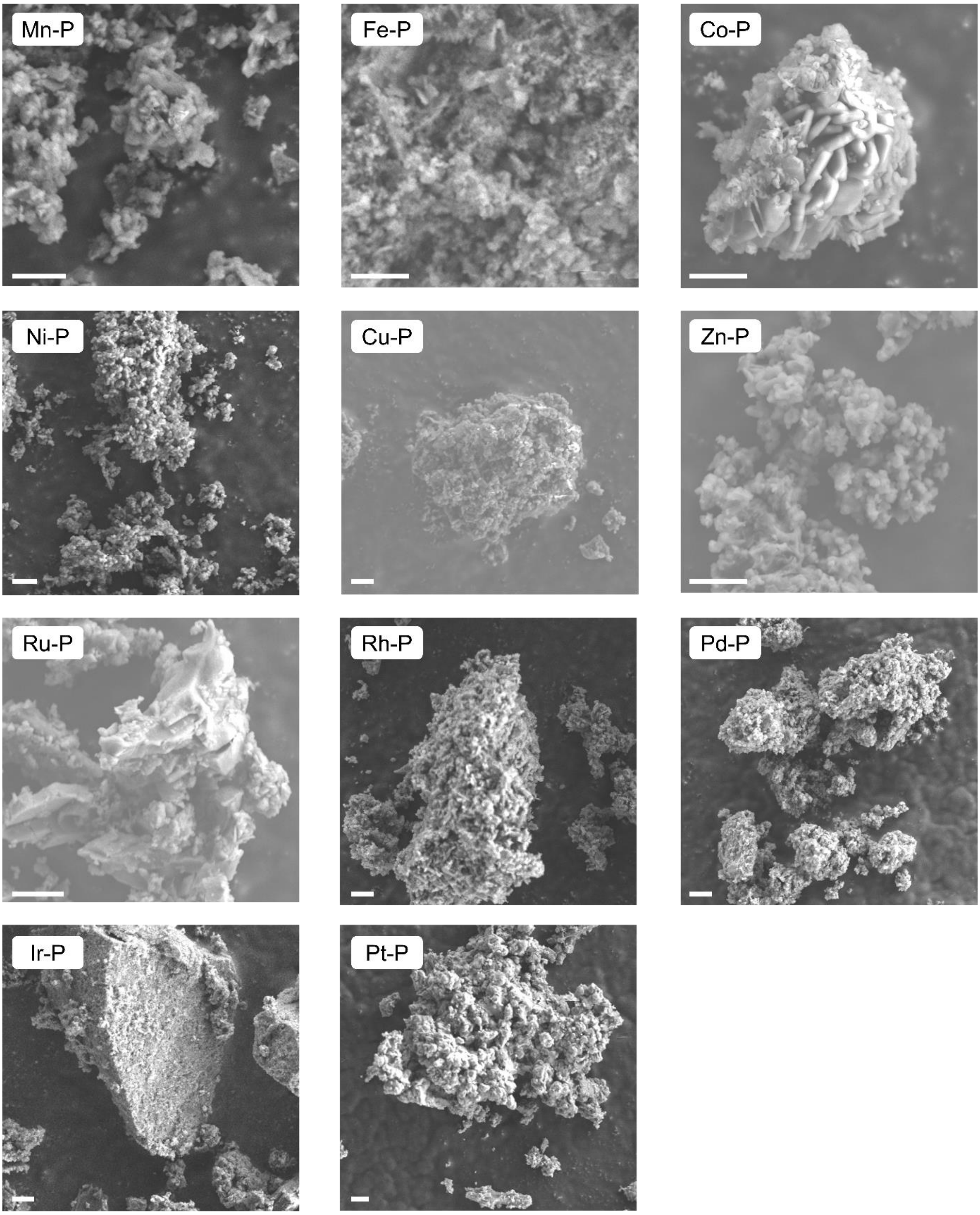

Figure S3 | SEM images of the HD-SACs. Scale bar 1 μm.

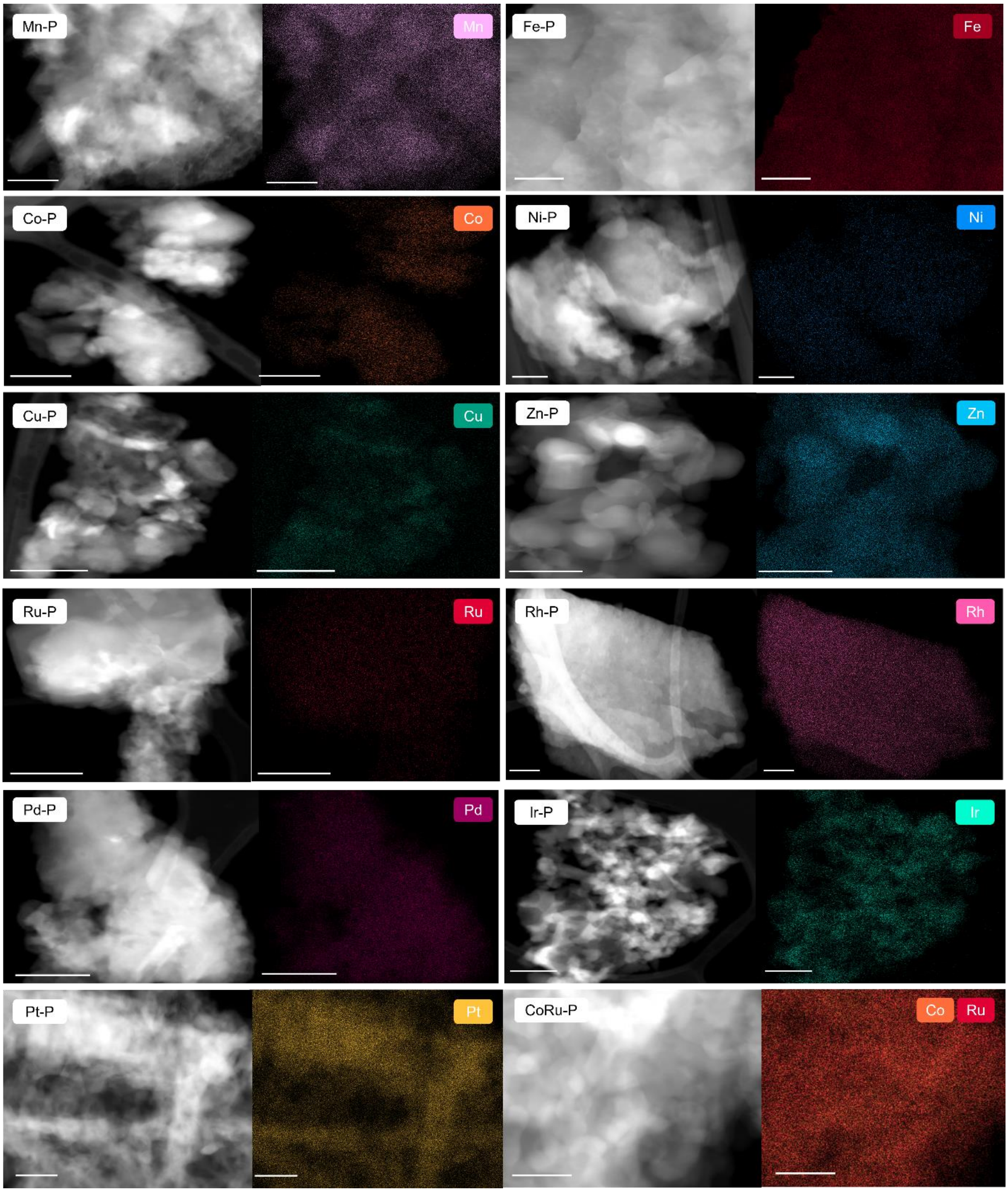

Figure S4 | Elemental mapping of monometallic HD-SACs. HDAAF-STEM image and corresponding EDX maps of various metal polymers, evidencing the uniform dispersion of metals over the whole structure. Scale bars 50 nm (**Fe-P**, **Ni-P**), 100 nm (**Co-P**, **Cu-P**, **Rh-P**, **Pd-P**, **Ir-P**, **Pt-P**, **CoRu-P**) and 200 nm (**Mn-P**, **Zn-P**, **Ru-P**).

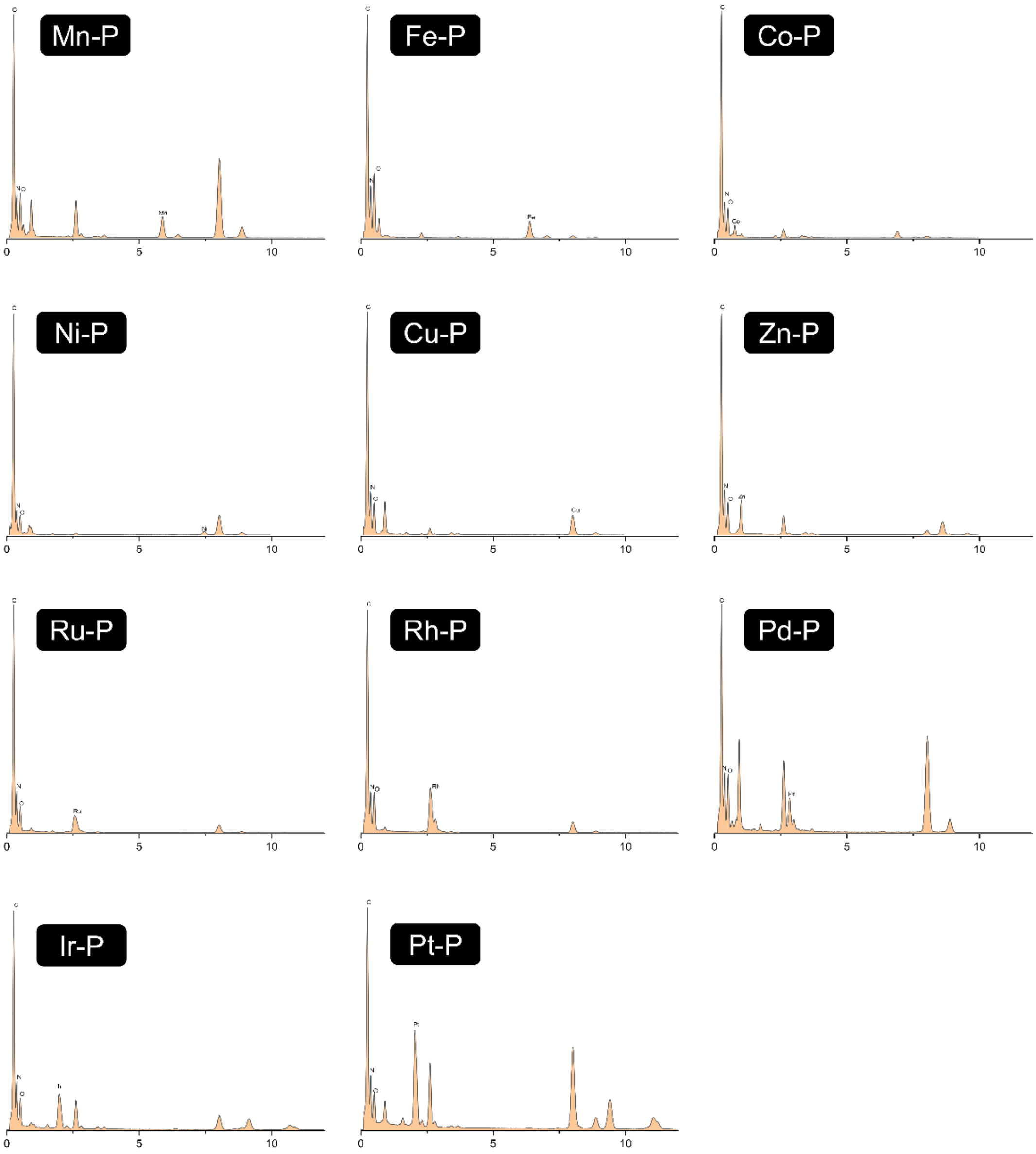

Figure S5 | EDX spectra of the monometallic HD-SACs corresponding to the elemental maps in **Figure 1**.

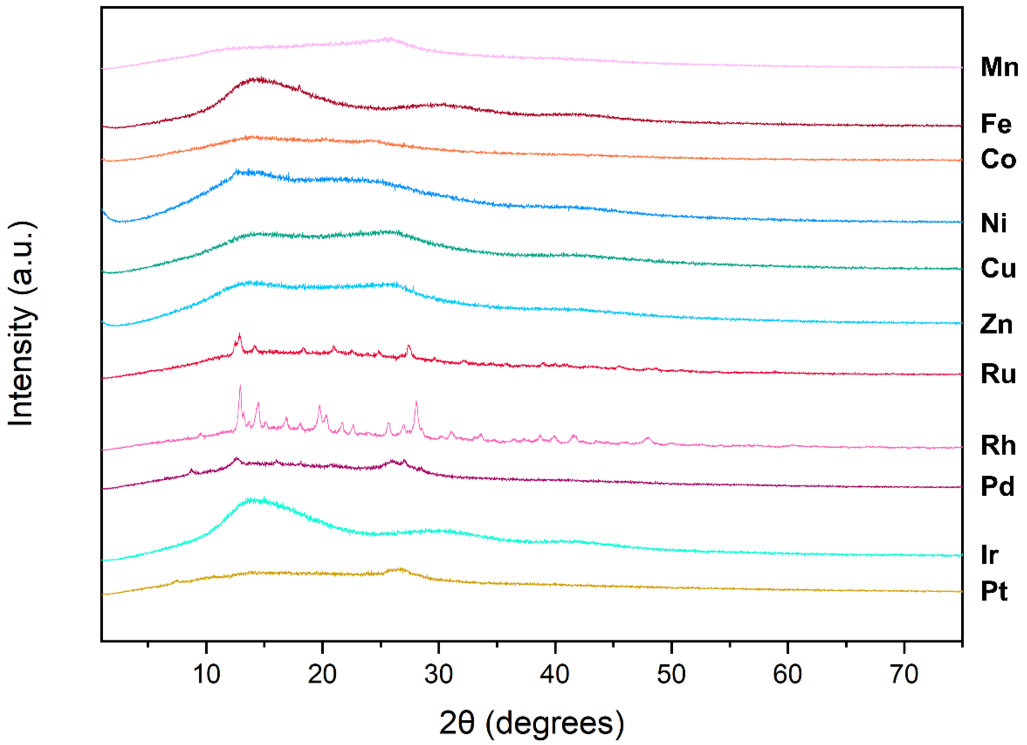

Figure S6 | XRD spectra of the respective HD-SACs. Distinct peaks are observed for **Rh-P** and **Ru-P** and are not identical to **Rh-M** and **Ru-P**, indicating the formation of an ordered crystalline polymer structure. The crystalline material could not be extracted or isolated. Moreover, the crystalline phase was not found in the ICDD database and micro-electron diffraction analysis did not yield any suitable structures.

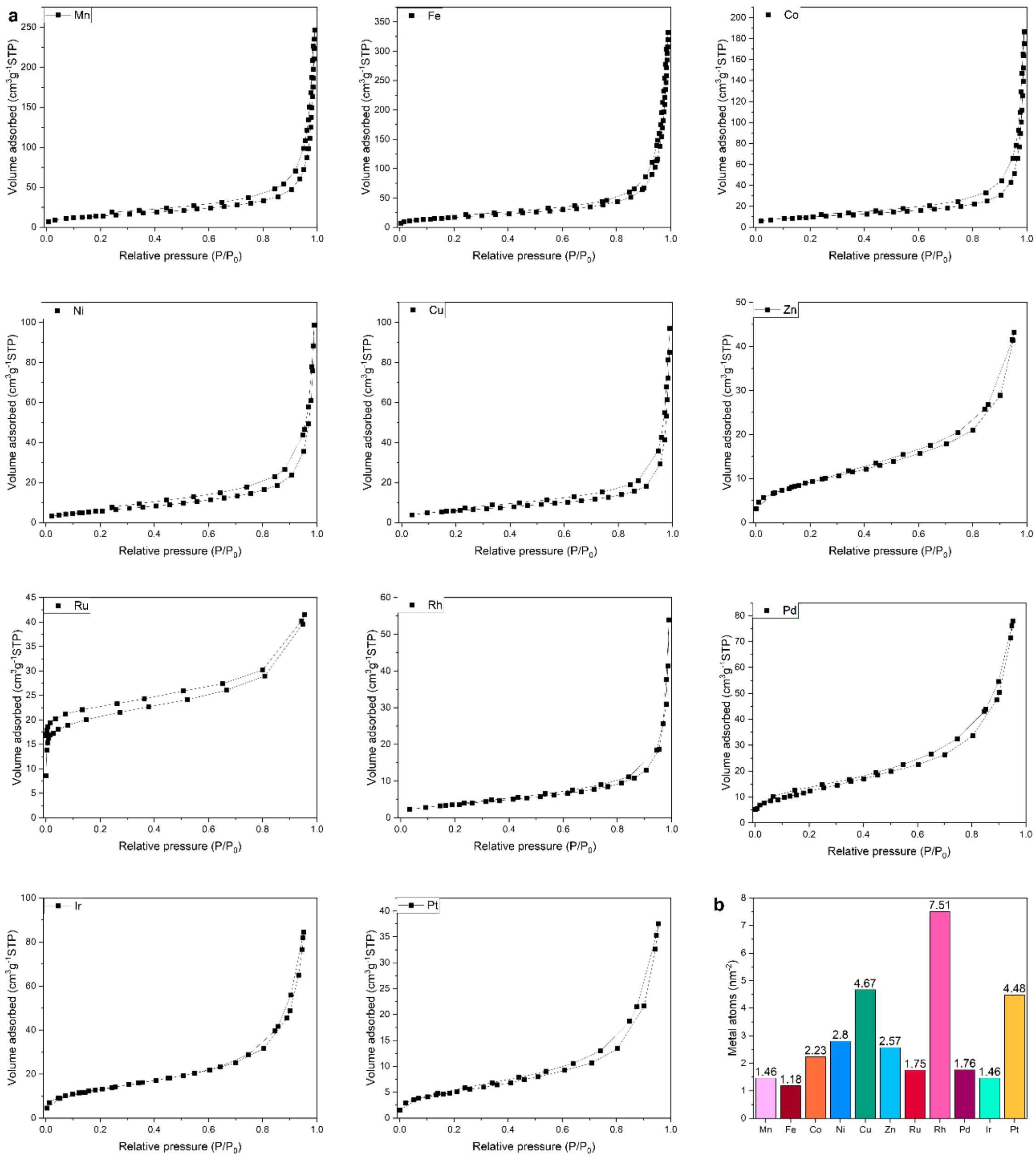

Figure S7 | BET N_2 adsorption-desorption isotherms of the HD-SACs recorded at 77 K (**a**) and areal density of metal atoms in the HD-SACs (**b**).

The areal density of metal atoms was calculated using the BET surface area and the bulk metal loading. In the context of coordination polymers, this value represents the total number of metal atoms per nm² of surface area accessible to gas-phase adsorbates (as measured by BET), and not necessarily the number of catalytically exposed metal sites. Therefore, a direct comparison with literature values for surface area atoms is not possible.

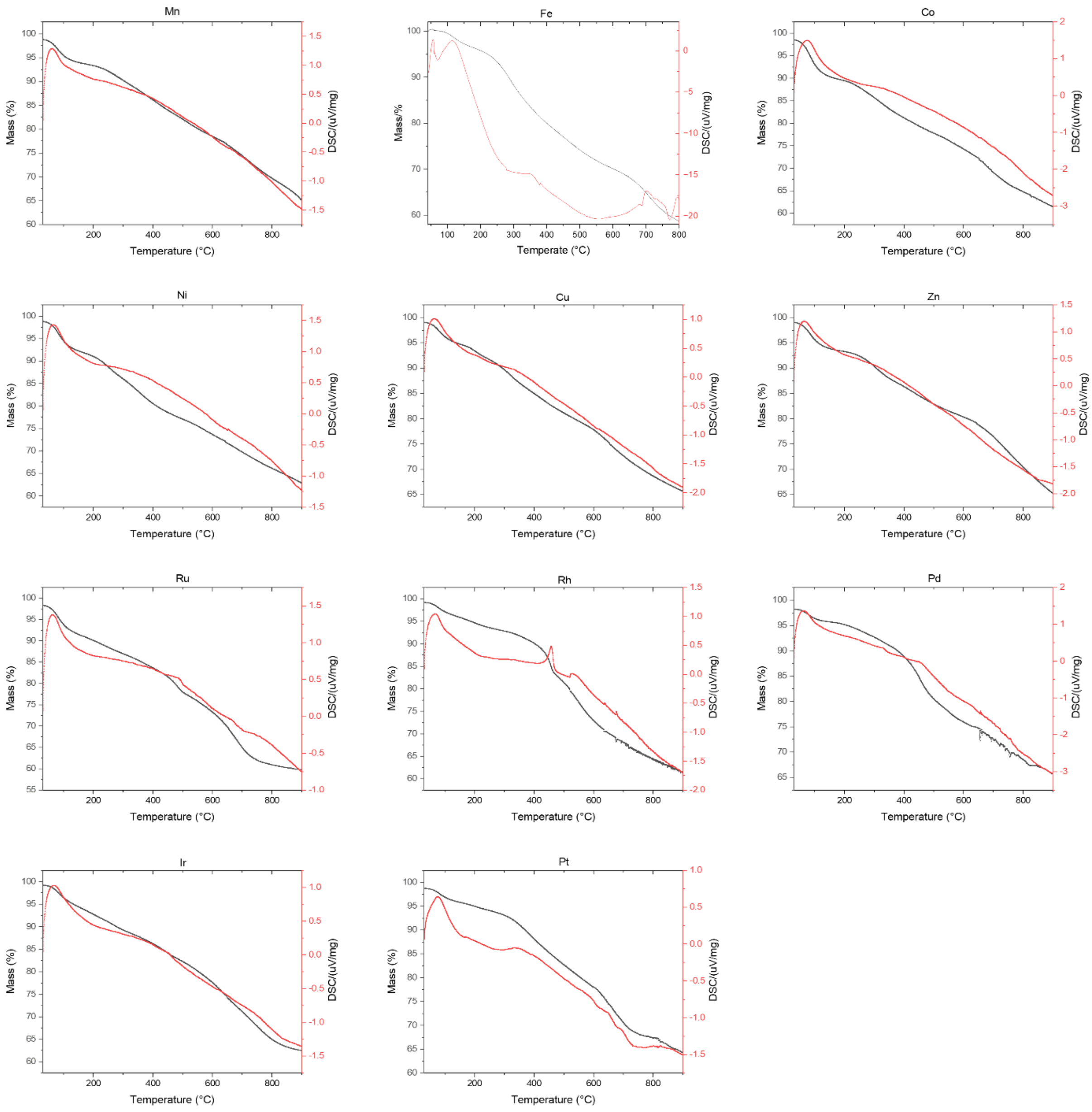

Figure S8 | TGA and DSC analysis of the HD-SACs.

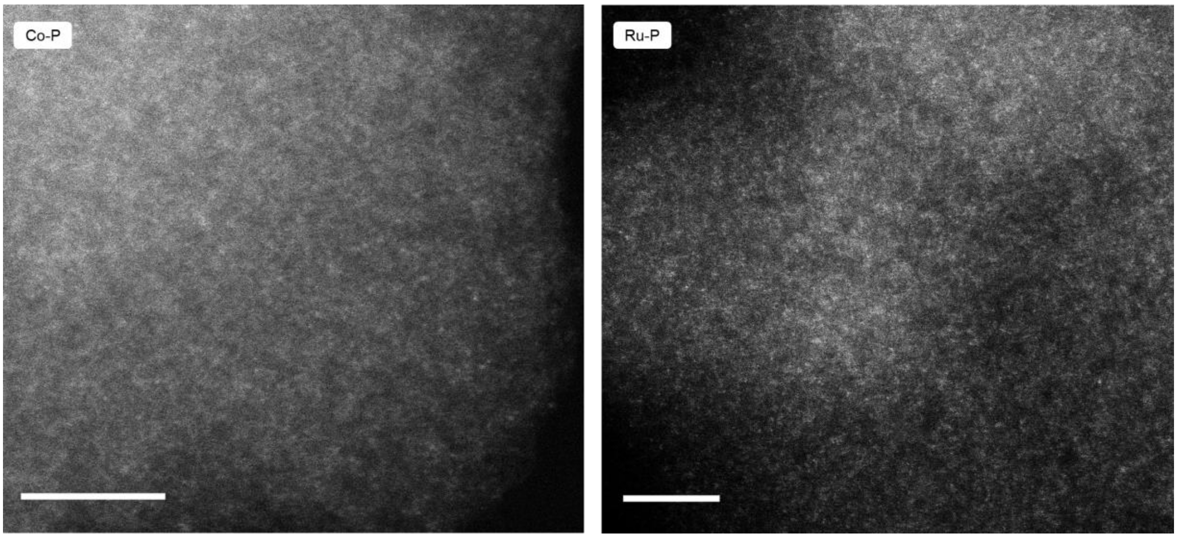

Figure S9 | HR-STEM images of **Co-P** and **Ru-P** annealed in an inert atmosphere at 500 and 300 ºC respectively. Scale bar 5 nm.

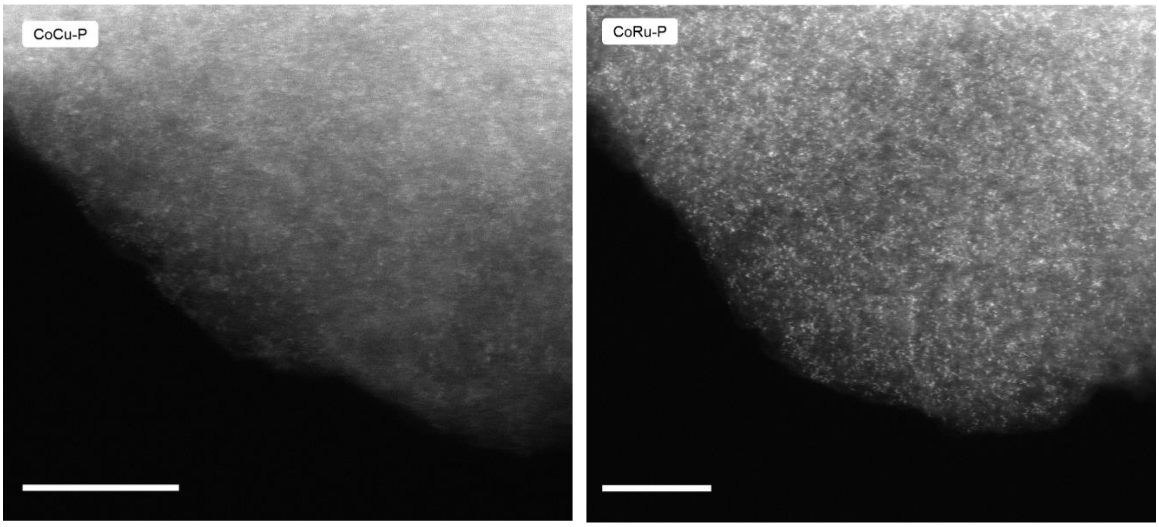

Figure S10 | HR-STEM images of **CoCu-P** and **CoRu-P**. The brighter dots in **CoRu-P** are attributed to Ru atoms. Scale bar 5 nm.

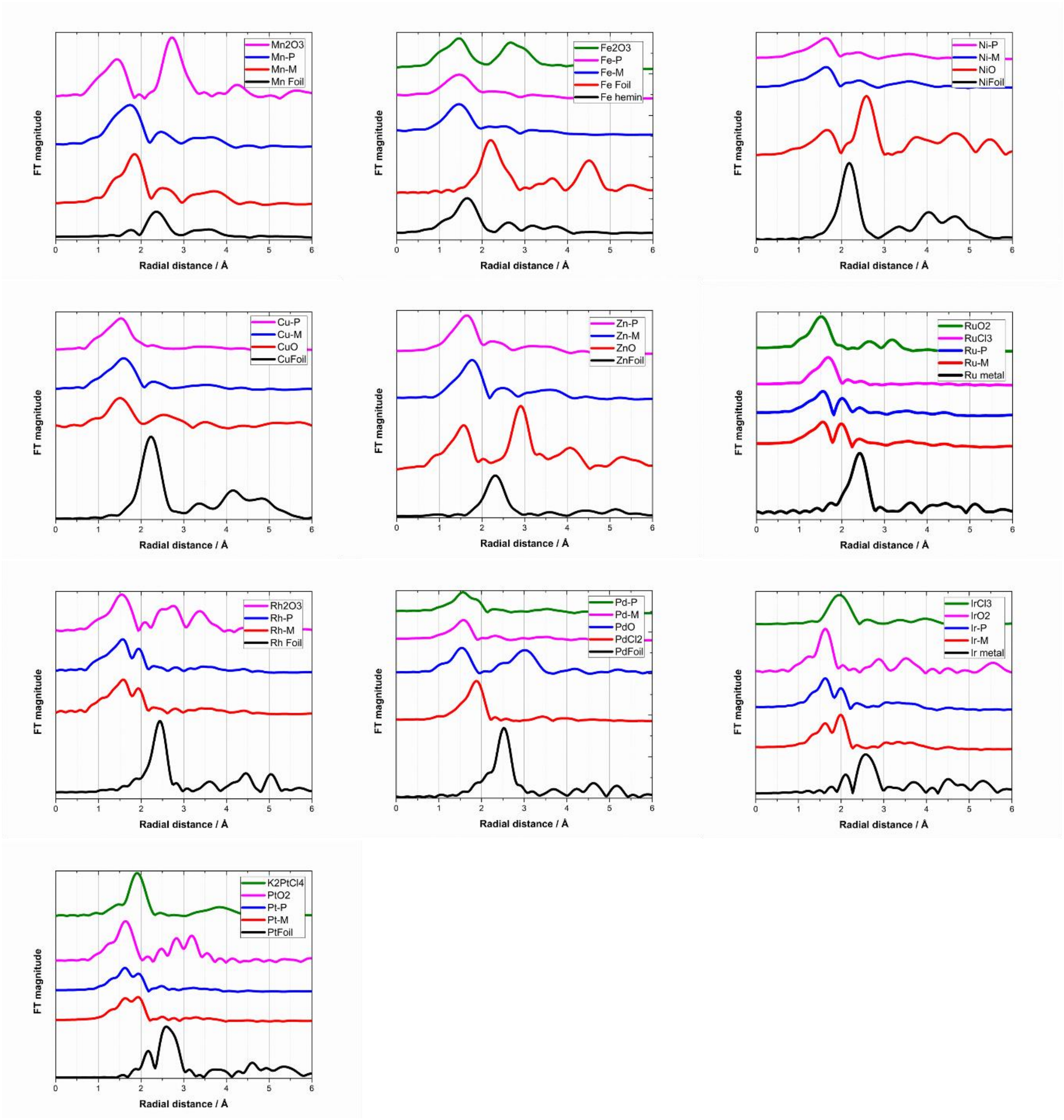

Figure S11 | Fourier transformation (not corrected for phase shift) of the EXAFS spectra of the metal monomer precursors, HD-SAC polymers and respective references.

Table S1 | EXAFS spectra fitting of the monomers. Coordination number (C.N.), interatomic distances ($R_{final} = R_0 + \Delta R$), energy shift ($\Delta E_0$), Debye Waller-factors (σ^2), and R-factor (the goodness of fit parameters).

Monomer	Shell	C.N.	S_0^2	ΔE_0/eV	R_0 / Å	R / Å	σ^2 / Å^2	R-factor
**Mn**	Mn-N	2+2 (fixed)	0.87	-5.2 +/- 4.2	2.274/2.324	2.141/2.190	0.009	0.020
	Mn-Cl	2 (fixed)			2.462	2.432	0.002	
**Fe**	Fe-N	6 (fixed)	0.75	3.65 +/- 2.1	1.981	2.012	0.006	0.025
**Co**	Co-N	6 (fixed)	0.74	7.9 +/- 2.1	2.120	2.199	0.003	0.025
**Ni**	Ni-N	6 (fixed)	0.80	-0.62 +/- 1.16	2.087	2.086	0.005	0.007
**Cu**	Cu-N	2+2 (fixed)	0.85	-1.25 +/- 1.9	2.183/2.239	1.952/2.008	0.006	0.015
	Cu-Cl	2 (fixed)			2.400	2.257	0.007	
**Zn**	Zn-N	2+2 (fixed)	0.74	5.1 +/- 1.7	2.191/2.251	2.148/2.208	0.002	0.018
	Zn-Cl	2 (fixed)			2.399	2.435	0.006	
**Ru**	Ru-N	2+2 (fixed)	0.77	2.7 +/- 0.6	2.028/2.058	2.025/2.055	0.002	0.007
	Ru-Cl	2 (fixed)			2.44	2.42	0.003	
**Rh**	Rh-N	4 (fixed)	0.88	-0.3 +/- 1.2	2.040	2.039	0.0020	0.016
	Rh-Cl	2 (fixed)			2.324	2.332	0.0024	
**Pd**	Pd-N	4 (fixed)	0.86	3.55 +/- 1.9	2.049	2.035	0.0020	0.019
**Ir**	Ir-N	4.035 +/- 0.75	0.7	4.65+/-0.75	2.049	2.035	0.0028	0.004
	Ir-Cl	3.50 +/- 0.5			2.343	2.360	0.0025	
**Pt**	Pt-N	2.7 +/- 0.84	0.85	6.75+/-2.05	2.096	2.009	0.0027	0.010
	Pt-Cl	1.83 +/- 0.6			2.360	2.283	0.0023	

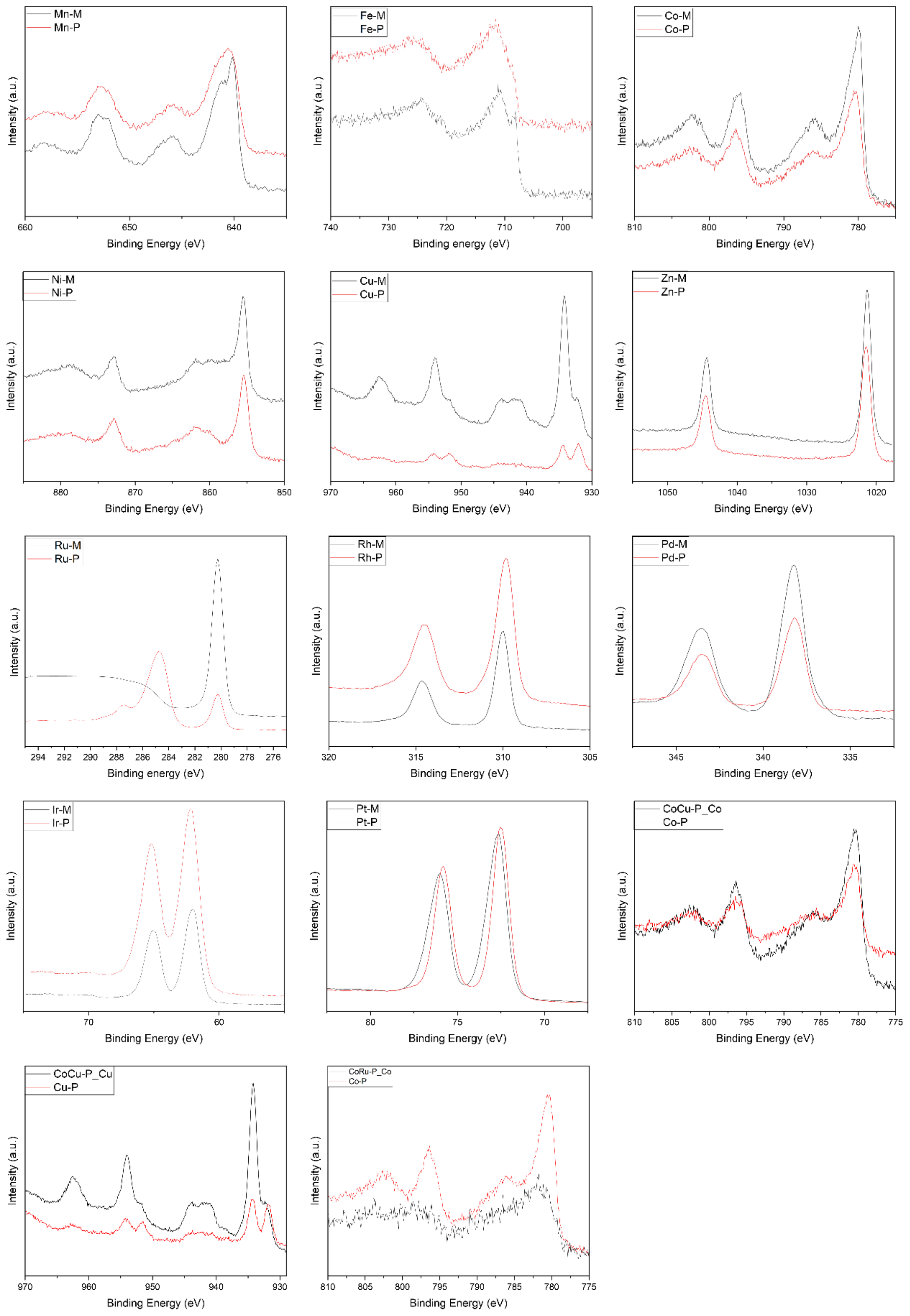

Figure S12 | XPS metal edge spectra of the respective metal monomer and HD-SACs.

Table S2 | Oxidation state of the metal monomer derived from the XANES spectra.

Metal	Edge	Monomer valence state	Polymer valence state
**Mn**	K 6.5390 keV	~2+	No substantial change
**Fe**	K 7.1120 keV	~3+	Minor differences
**Co**	K 7.7089 keV	~2+	Minor oxidation
**Ni**	K 8.3328 keV	~2+	No substantial change
**Cu**	K 8.9789 keV	~2+	Mixture 1+ and 2+
**Zn**	K 9.6586 keV	~2+	No substantial change
**Ru**	K 22.1172 keV	~2.5+	No substantial change
**Rh**	K 23.2199 keV	~3+	No substantial change
**Pd**	K 24.3503 keV	~2+	Minor reduction
**Ir**	L3 11.2152 keV	~3+ - 4+	Minor oxidation
**Pt**	L3 11.5637 keV	~2+	Minor differences

Note: Minor changes are observed for **Co-P**, that becomes slightly more oxidized. **Cu-P** is present as a mixture of +1/+2.

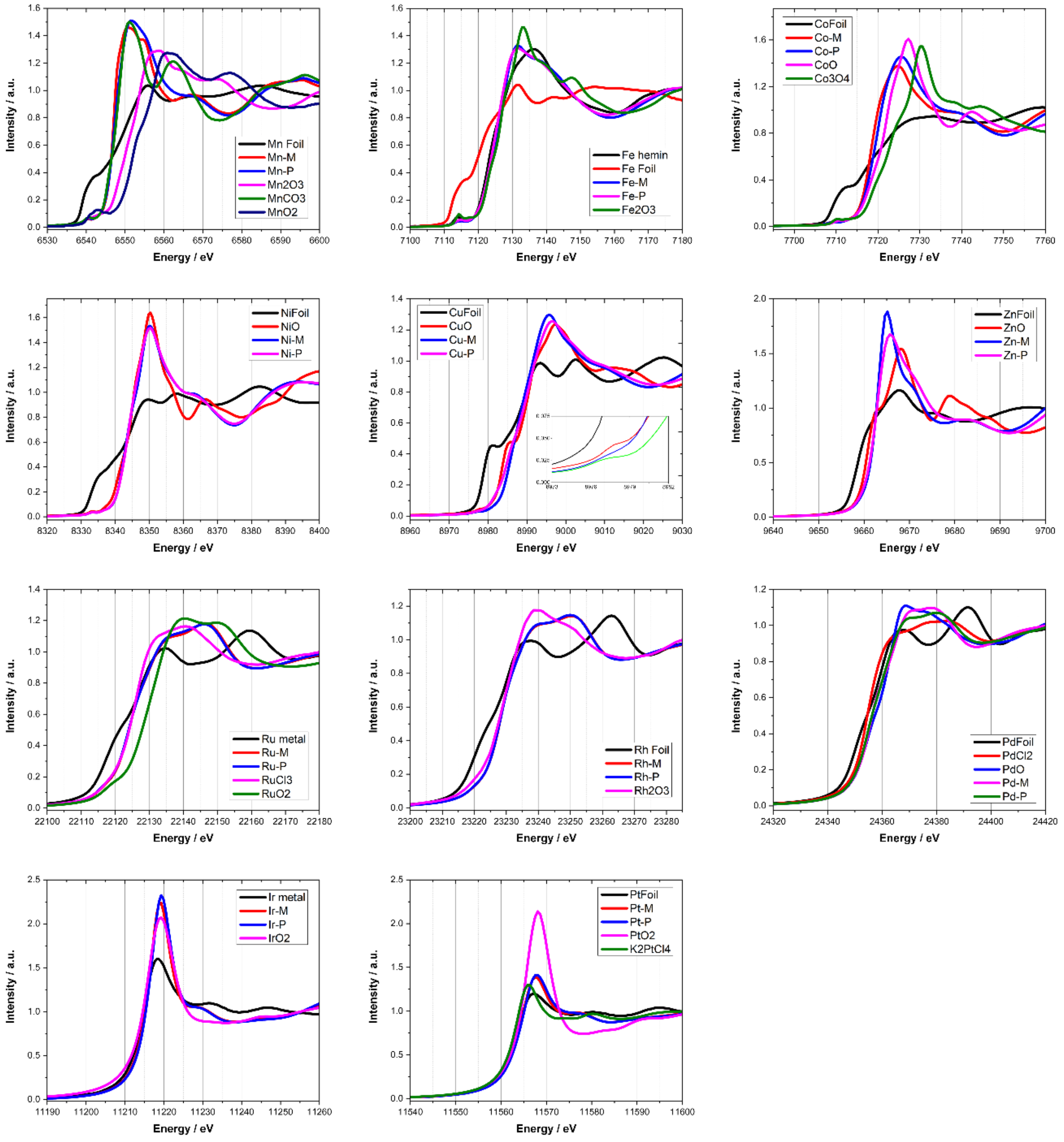

Figure S13 | XANES spectra of the metal monomer precursors, HD-SAC polymers and respective references.

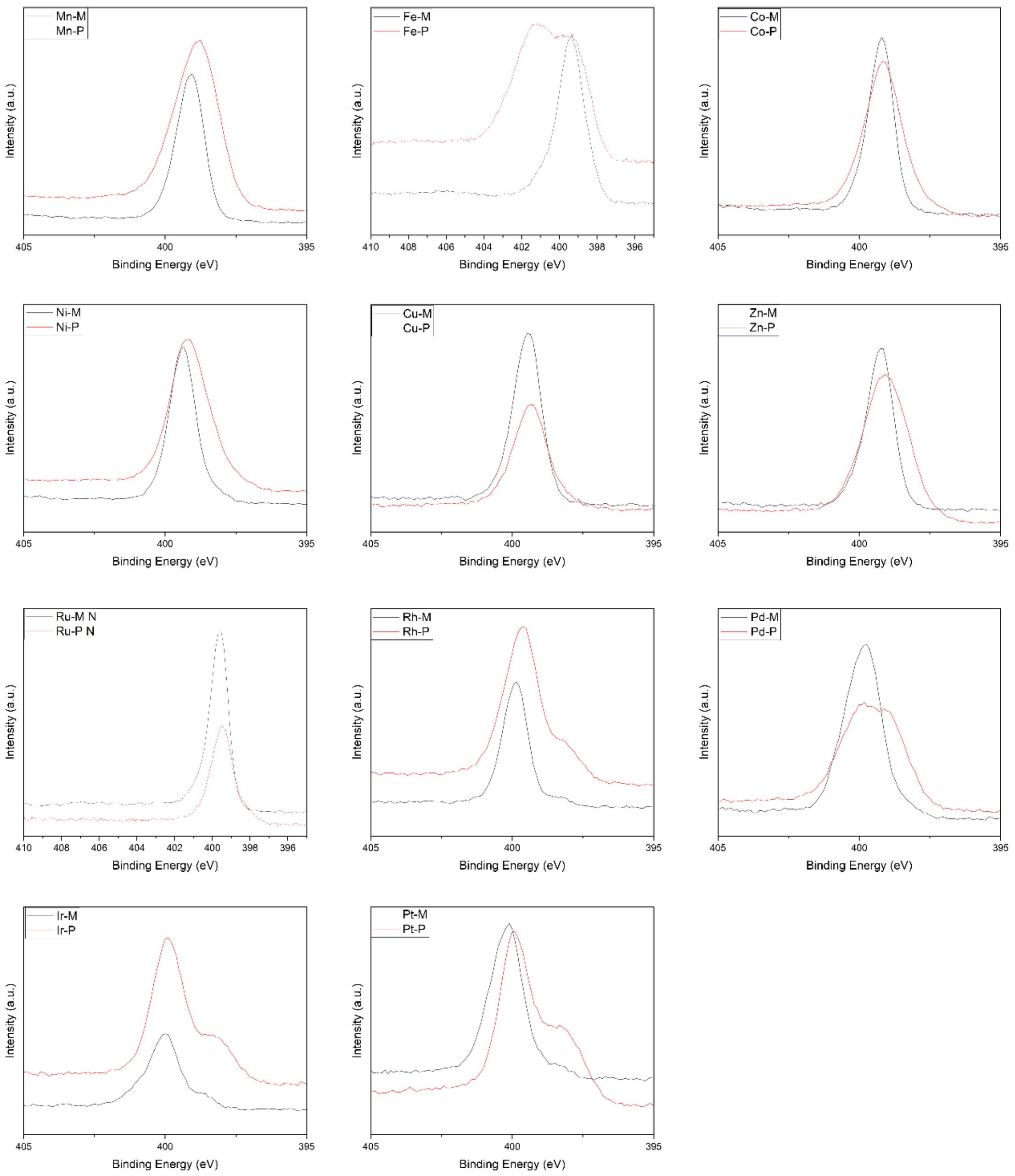

Figure S14 | XPS N 1s edge spectra of the respective metal monomer and HD-SACs. The intensity of the N 1s XPS signal increases in the HD-SACs, arising from the new pyrazine nitrogen atoms in the structure, accompanied by a slight shift to lower binding energies, which could be a consequence of the higher conjugation in the **tatppb** structure. Broadening or the appearance of a second contribution is observed in the HD-SACs.

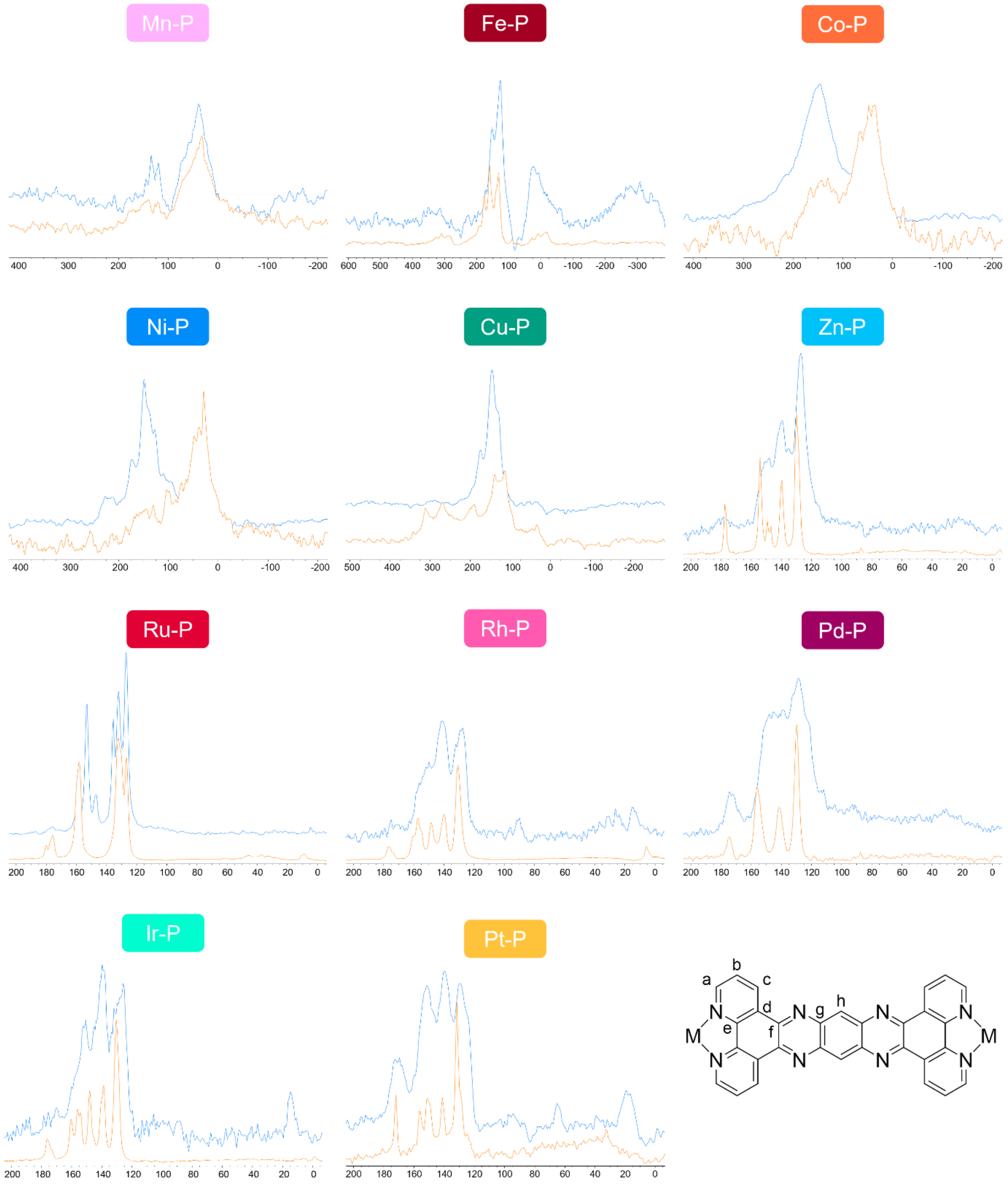

Figure S15 | ^{13}C solid state NMR spectra of the metal monomers (orange) and the HD-SACs (blue). The paramagnetic samples are characterized by broad peaks across a wide chemical shift range (−400 ppm to +600 ppm). Dipolar coupling to unpaired electrons and quadrupolar nuclei complicates the analysis. Overall, the spectra of these HD-SACs appears to be broader than the monomers, likely a consequence of the polycondensation and the more complex chemical environments. The spectra of the diamagnetic samples are characterized by a disappearance or significant decrease

of the intensity of the carbonyl carbon peak (~175 ppm), an upfield shift of the carbon peaks alpha to the carbonyl (C_d) and meta to the nitrogen (C_b) in the 1,10-phenanthroline fragment (150–160 ppm) and appearance of new peaks corresponding to the pyrazine fragment (C_h ~135 ppm, C_g ~140 ppm, and C_f ~155).

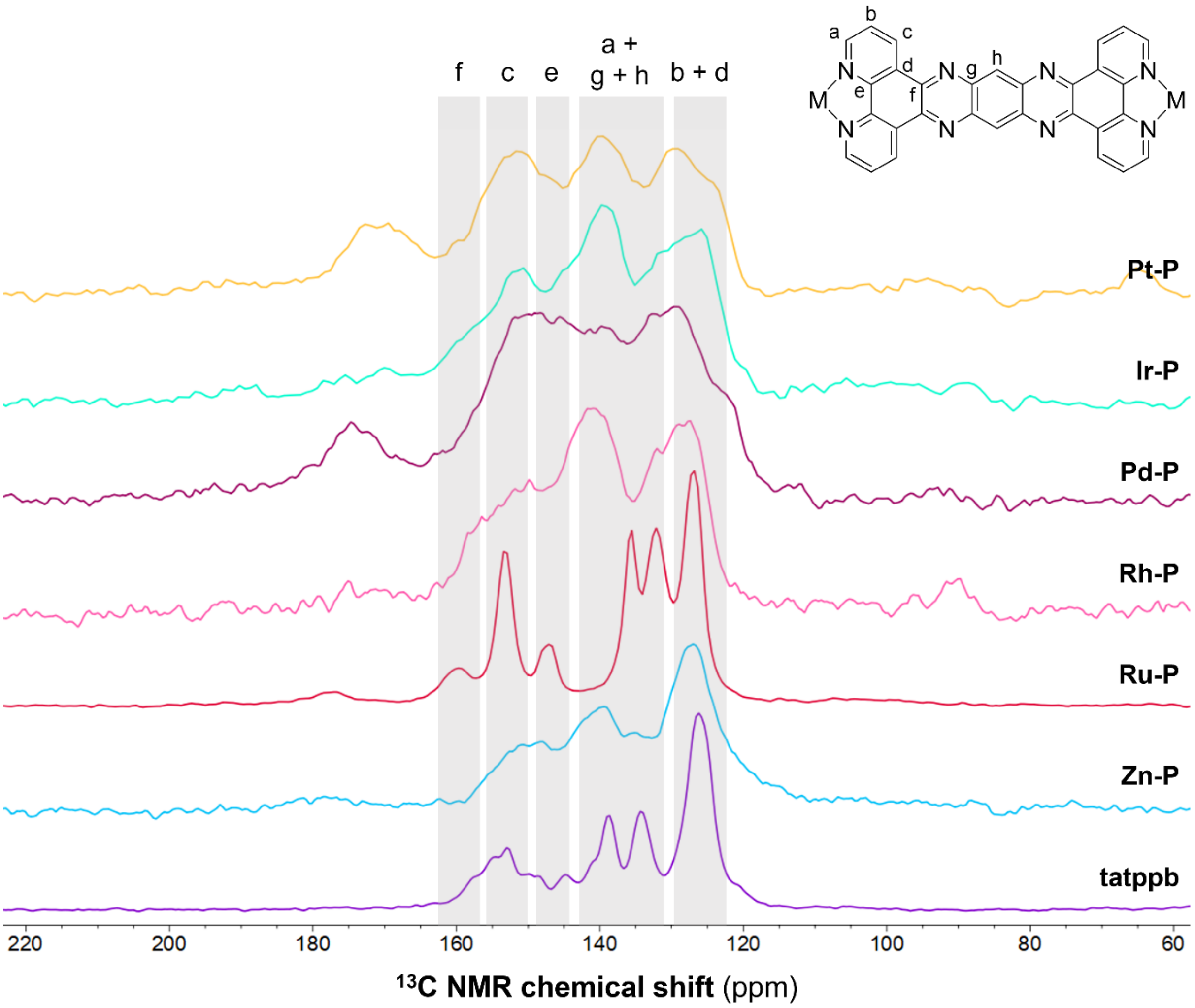

Figure S16 | ^{13}C solid state NMR spectra of the HD-SACs with diamagnetic metals and **tatppb**. Comparison of the spectra reveals three major areas that have better or worse peak separation and resolution depending on the species. A first area between 120 ppm and 130 ppm, containing the peaks for the *meta*-C–H carbon and the *meta*-quaternary carbon in the 1,10-phenanthroline fragment; a second one between 130 ppm and 143 ppm, containing the pyrazine C–H and C=N carbons and the *orto*-C–H carbon in the 1,10-phenanthroline fragment; and finally, a last area between 145 ppm and 165 ppm grouping the peaks for the *para*-C–H, the *orto*-quaternary carbon, and the C=N carbon in the 1,10-phenanthroline fragment.

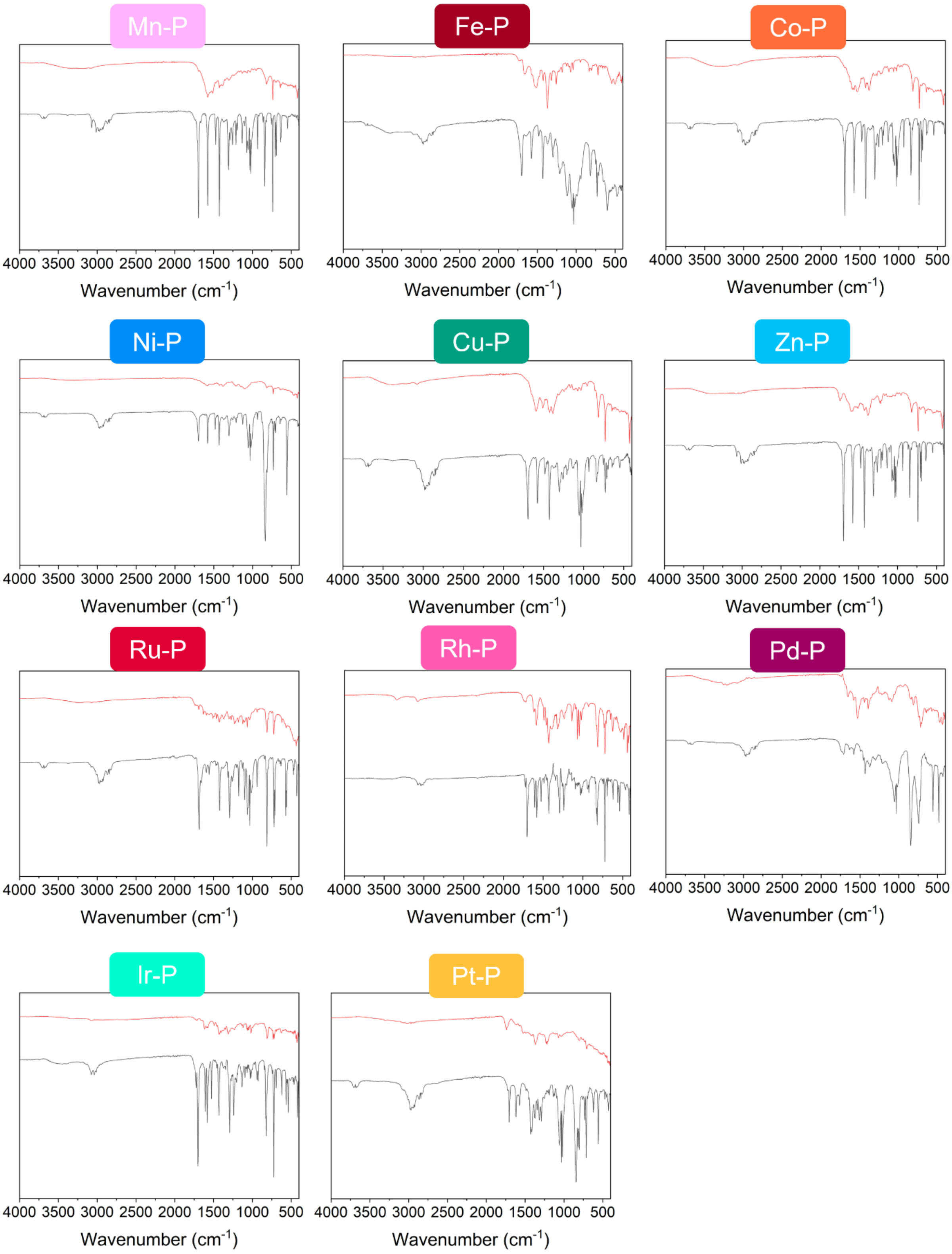

Figure S17 | FTIR spectra of the metal monomers (red) and HD-SACs (black). The strong C=O bond stretch (~1700 cm^{-1}) visible in the monomers, disappears in the HD-SACs samples, indicating the successful polycondensation reaction. The most important peaks are reported in the experimental section.

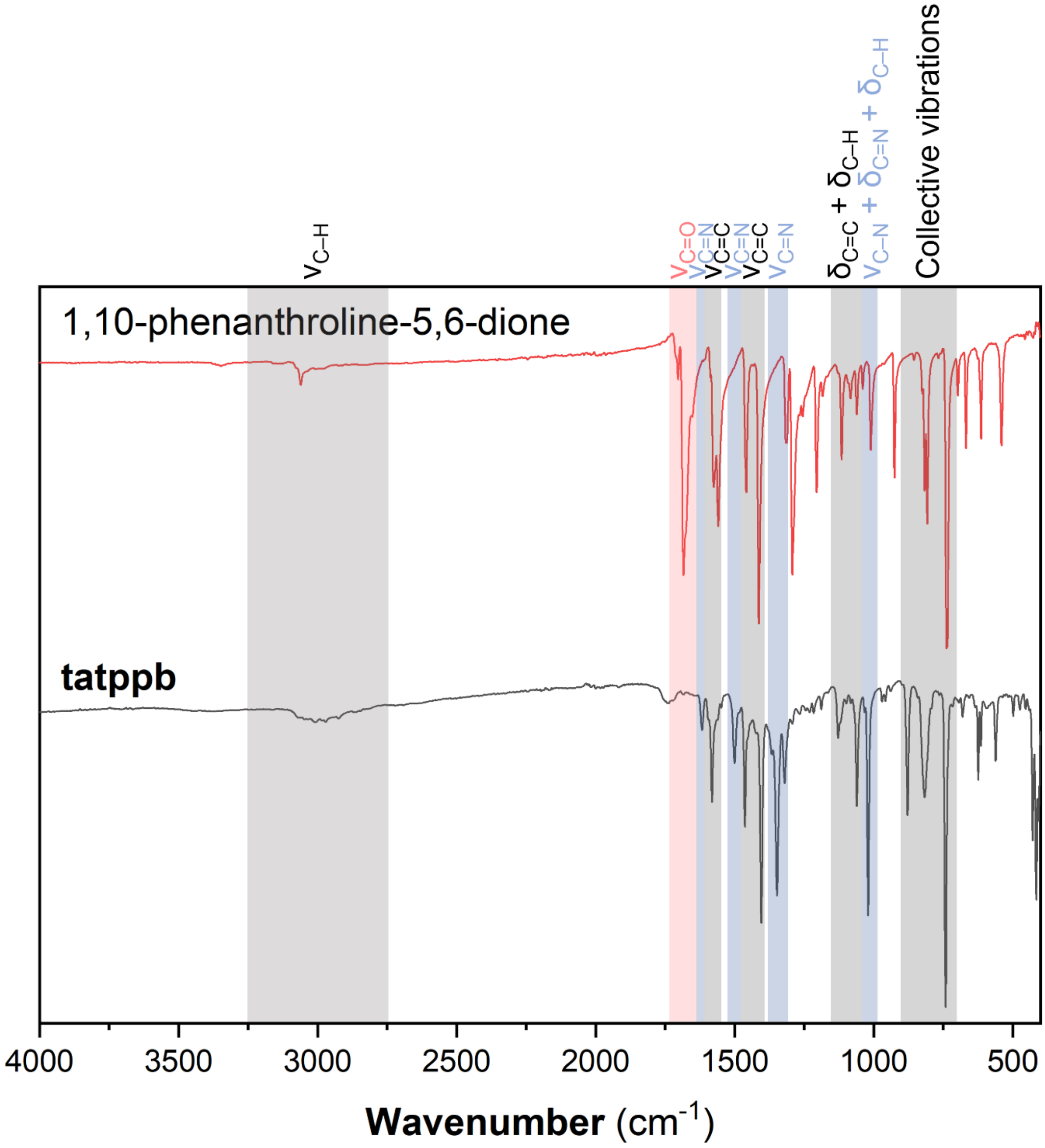

Figure S18 | FTIR spectra of 1,10-phenanthroline-5,6-dione and **tatppb**. Significant vibrational modes have been highlighted. In red, the C=O bond stretch (1684 cm^{-1}) of 1,10-phenanthroline-5,6-dione disappears upon reaction. In blue, C–N and C=N bond vibrations arising from the phenanthroline and the pyrazine moieties increase in intensity or appear due to the imine condensation. In gray, C–H and C–C bond vibrations and collective vibrations of the molecule including breathing modes. Further description of the peaks is included in the experimental section.

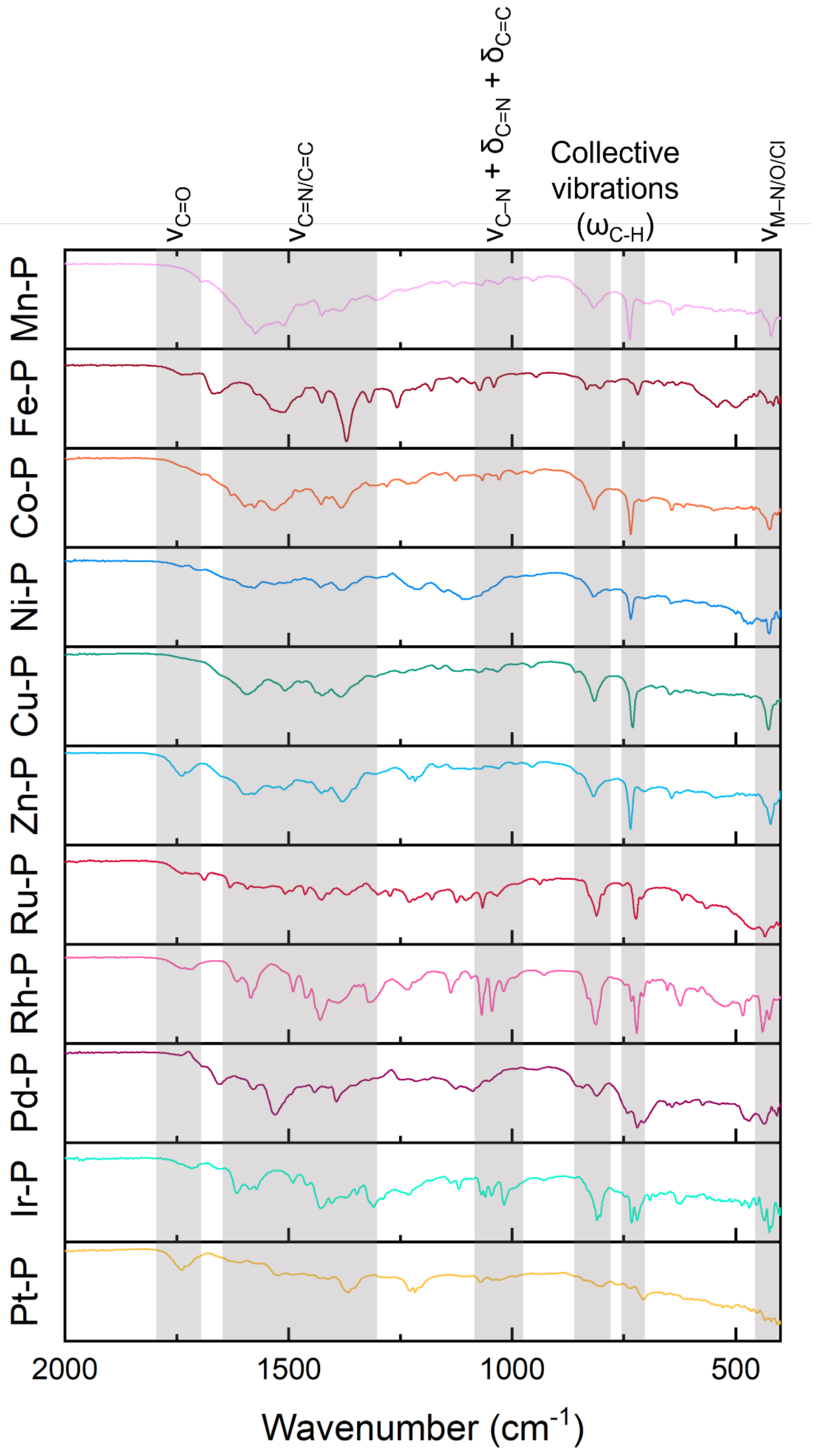

Figure S19 | FTIR spectra (400–2000 cm^{-1}) of the HD-SACs. Representative vibrations in the HD-SACs that are consistently present across the samples are highlighted. The most important peaks are reported in the experimental section.

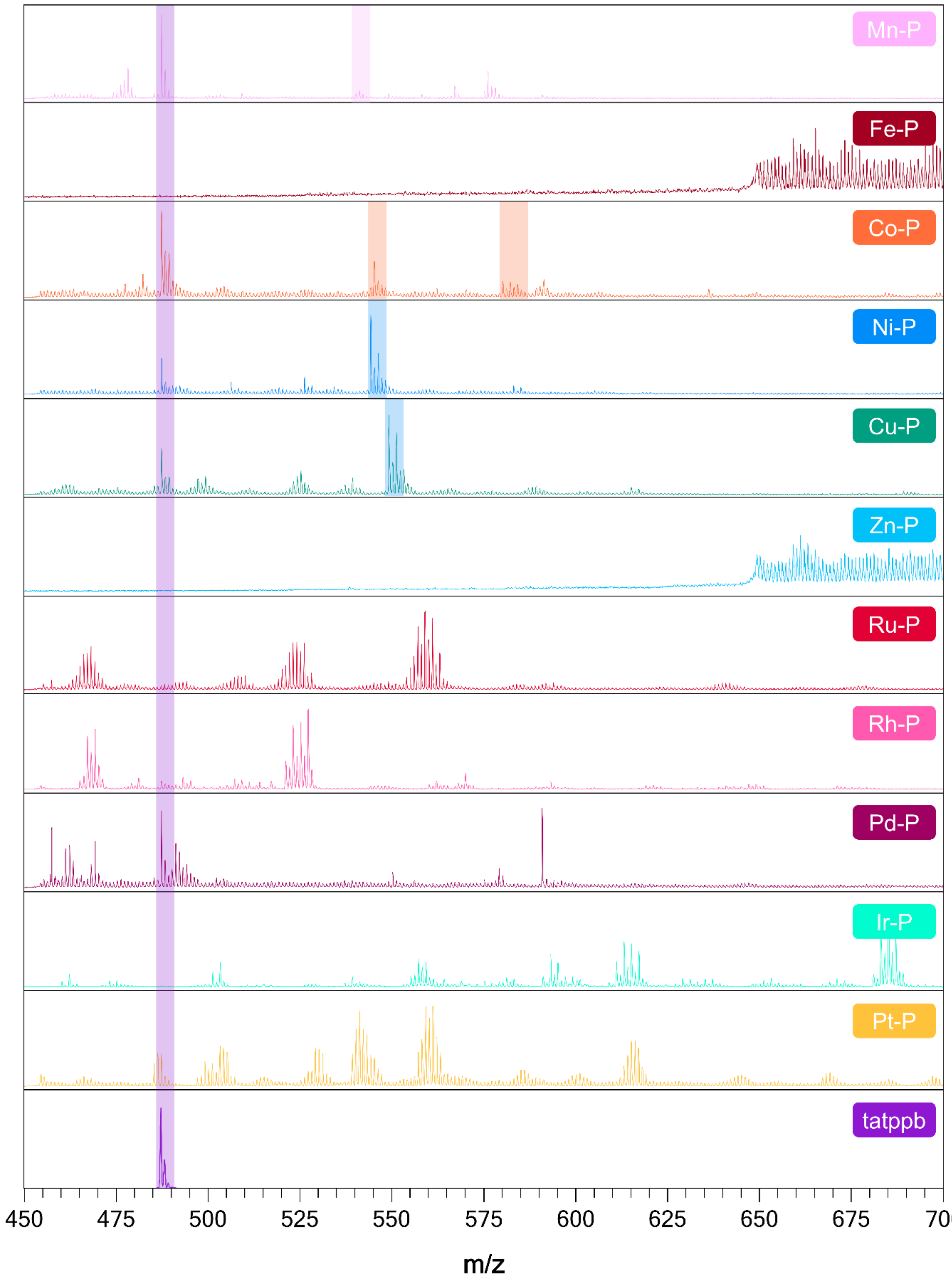

Figure S20 | MALDI spectra of the HD-SACs. When isolated and unequivocally identifiable, ions arising from **tatppb** and of **tatppb** with the corresponding metal (and sometimes additional ligands) are highlighted.

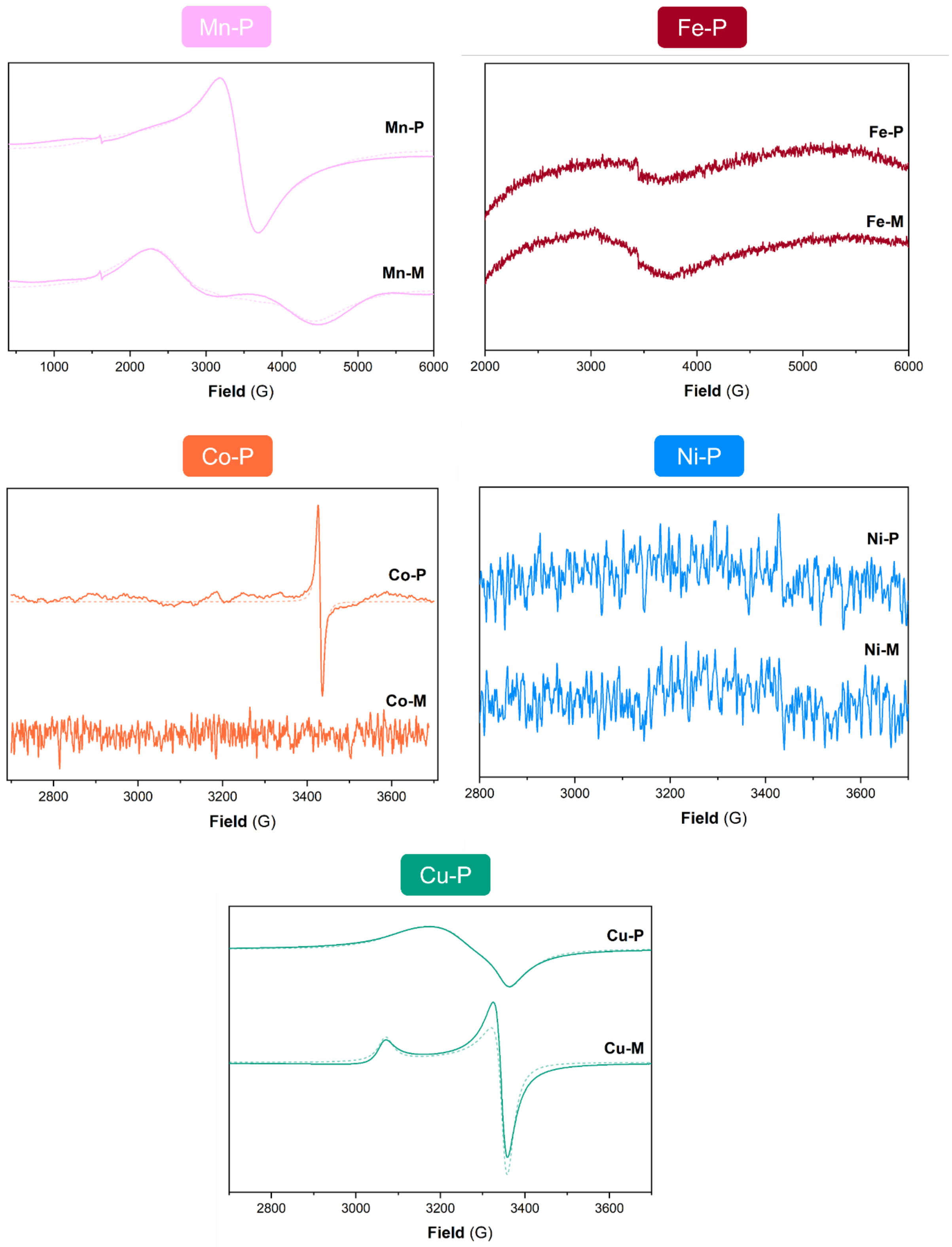

Figure S21 | X-band EPR spectra of the paramagnetic metal monomers precursors and corresponding HD-SACs (solid lines) and, when possible, fitted spectra (dashed lines). EPR spectra of **Fe-M**, **Fe-P**, **Ni-M** and **Ni-P** did not contain any peaks. Integer spin transition metals (Ni^{II}: d^8, S =1; and high spin Fe^{II}: d^6, S = 2) are usually "EPR-silent" due to the large magnitude of the zero-field splitting (ZFS) in these

complexes.[34] The axial ZFS parameter, $|D|$, of **Fe-M** and **Ni-M** were calculated, resulting in values larger than the employed microwave frequency (3.8 and 0.8 cm^{-1}).[35] Additionally, the EPR spectrum of **Co-M** did not present any absorbance, likely arising from a high spin (Co^{II}: d^7, S = 3/2) state ($|D|$ = 2.5 cm^{-1}).[35,36] However, upon polymerization, the spectrum of **Co-P** has a relatively sharp signal (ΔH_{pp} = 1 MHz) with no anisotropy, indicating a highly symmetric environment in the low spin state (S = 1/2) consistent with the expected structure. The spectrum of **Mn-M** contains a complex signal that was successfully fitted to a high spin nucleus (Mn^{II}: d^5, S = 5/2) with ZFS. The *g* value is largely anisotropic (g_x = 1.474, g_y = 2.181, and g_z = 3.260) with broad linewidth (ΔH_{pp} = 37 MHz), and $|D|$ and $|E|$ (the equatorial ZFS parameter) are 0.024 cm^{-1} and 0.0028 cm^{-1}, respectively. The ZFS values are consistent with those reported for other octahedral Mn^{II} complexes.[37] The spectrum of the **Mn-P** is distinct with the appearance of a more symmetric signal which was modelled as a combination of a high spin nucleus (12%) and a second contribution from a low spin (S = 1/2) nucleus with an anisotropic *g* (g_x = 1.872, g_y = 1.993, and g_z = 2.101 and ΔH_{pp} = 27 MHz, 88%). The spectrum of **Cu-M** displays the typical shape for Cu^{II} with axially anisotropic $g_{\perp}$ = 2.056 and $g_{||}$ = 2.241, arising from the Jahn-Teller distortion of the structure. After polymerization, the $g_{\perp}$ component of the **Cu-P** spectrum is broader, indicating the presence of a distribution of sites with slightly different geometries, probably a consequence of the strain induced by the formation of the rigid polymer. The spectrum was modelled using five independent electron systems with axial symmetry ($g_{\perp}$ = 2.06 ± 0.03 and $g_{||}$ = 2.21 ± 0.17).

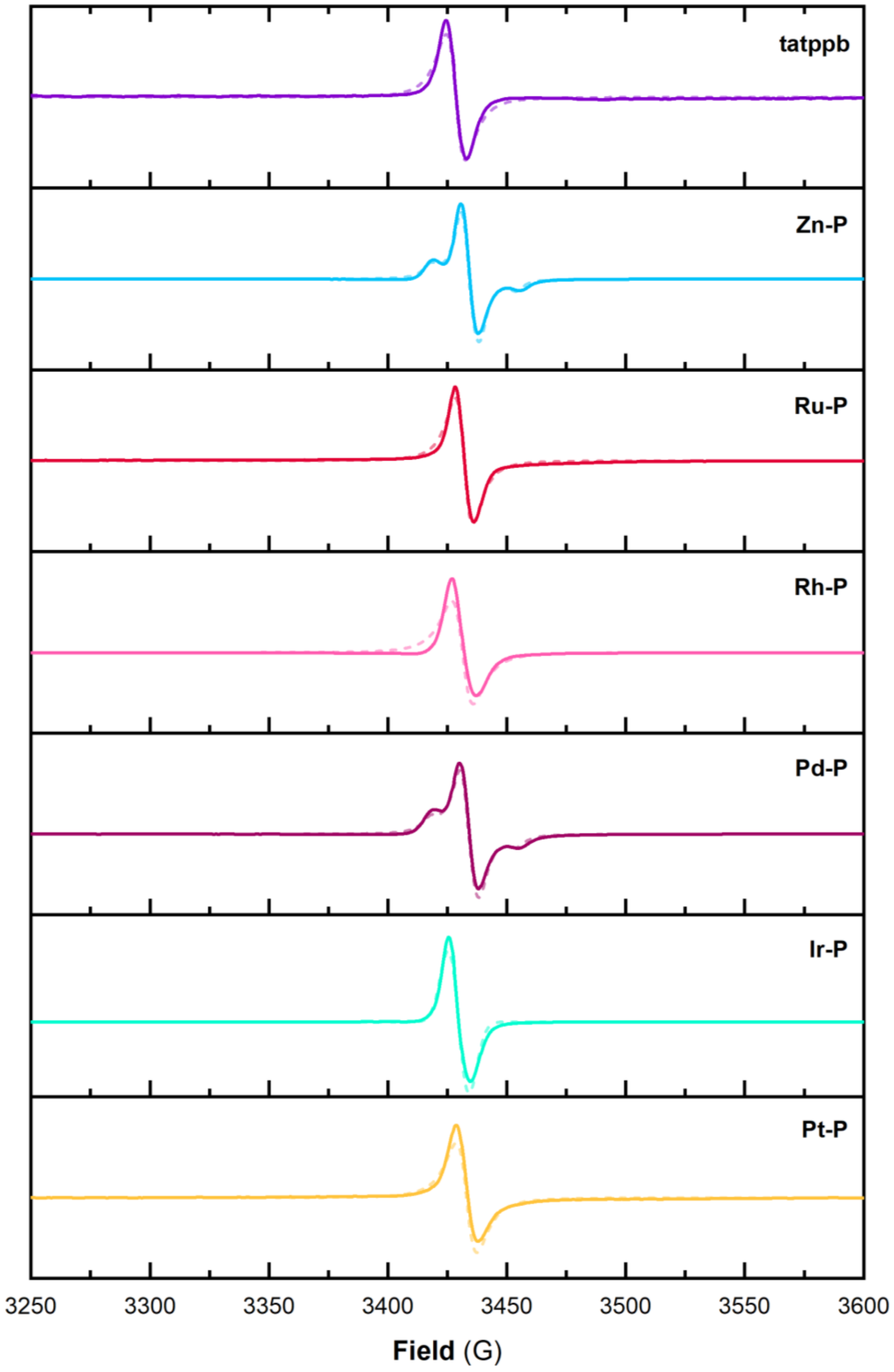

Figure S22 | X-band EPR spectra (solid line) and fitted spectra (dashed line) of **tatppb**, **Zn-P**, **Ru-P**, **Rh-P**, **Pd-P**, **Ir-P** and **Pt-P**. The EPR spectra of **Ru-P**, **Rh-P**, **Ir-P** and **Pt-P** were fitted to a singly-reduced species (S = 1/2) and are remarkably similar to the EPR spectrum of **tatppb**. The EPR spectra of **Zn-P** and **Pd-P** are more complex and were assigned to a doubly-reduced interconnecting ligand (S = 1).

Table S3 | EPR parameters from the fitted EPR spectra.

Sample	Spin	*g*-tensor	ΔH_{pp} (MHz)	$\lvert D \rvert$ (cm^{-1})	$\lvert E \rvert$ (cm^{-1})	$\lvert D \rvert / \lvert E \rvert$
**Mn-M**	S = 5/2	g_x = 1.4742<br>g_y = 2.1814<br>g_z = 3.2595	37	0.024	0.0028	0.12
**Mn-P**	S = 5/2 (12%)	g_x = 1.5999<br>g_y = 1.9712<br>g_z = 3.2302	33	0.022	0.0027	0.12
	S = 1/2 (88%)	g_x = 1.8723<br>g_y =1.9929<br>g_z = 2.1007	27	-	-	-
**Co-P**	S =1/2	g_{iso} = 2.0048	1.00	-	-	-
**Cu-M**	S = 1/2	$g_{\perp}$ = 2.0561<br>$g_{\parallel}$ = 2.2410	2.48	-	-	-
**Cu-P**	5 x S = 1/2	$g_{\perp}$ = 2.06 ± 0.03<br>$g_{\parallel}$ = 2.21 ± 0.17	5.8 ± 1.1	-	-	-
**tatppb**	S = 1/2	g_{iso} = 2.0045	0.979	-	-	-
**Zn-P**	S = 1	g_x = 1.9997<br>g_y = 2.0052<br>g_z = 2.0080	0.411	0.0011	0.0004	0.35
**Ru-P**	S = 1/2	g_{iso} = 2.0061	0.731	-	-	-
**Rh-P**	S = 1/2	g_{iso} = 2.0031	0.897	-	-	-
**Pd-P**	S = 1	g_x = 1.9997<br>g_y = 2.0053<br>g_z = 2.0080	0.498	0.0011	0.0004	0.35
**Ir-P**	S = 1/2	g_{iso} = 2.0068	0.876			
**Pt-P**	S = 1/2	g_{iso} = 2.0036	0.865	-	-	-

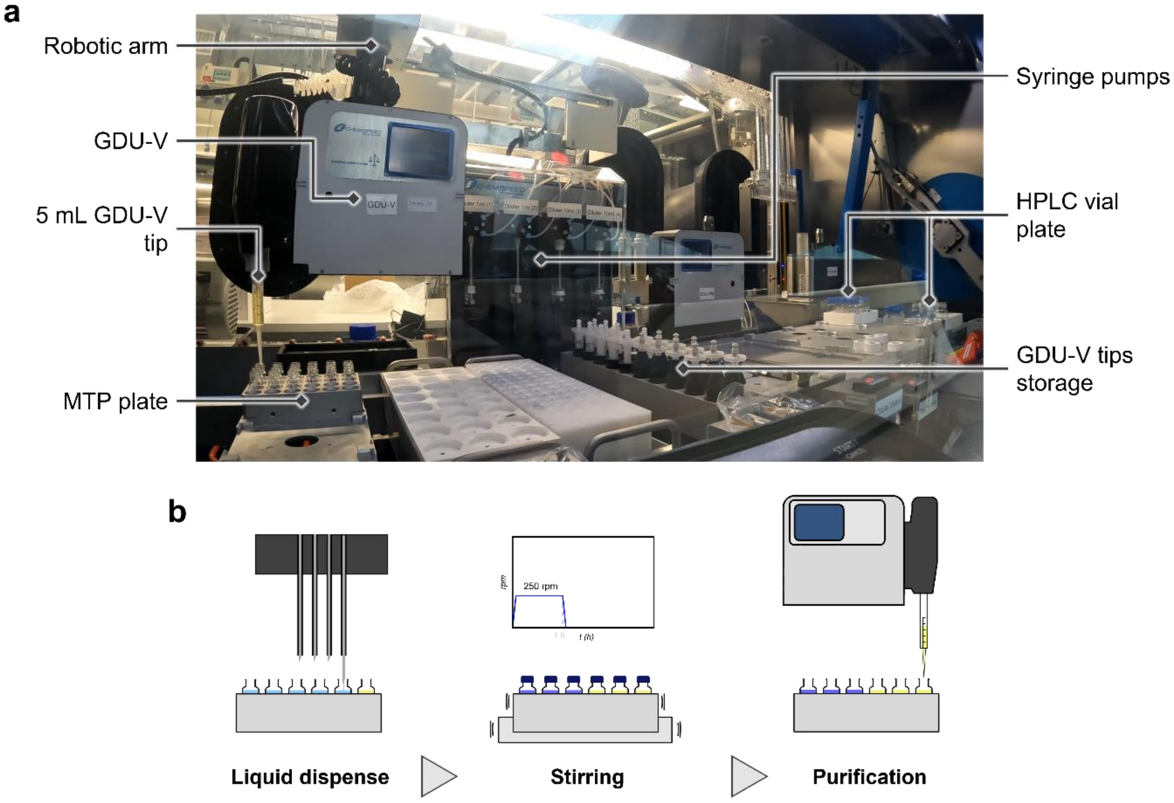

Figure S23 | Automated monomer synthesis procedure. Robotic synthesis platform and tools operated (**a**), and flowchart of the synthetic protocol (**b**).

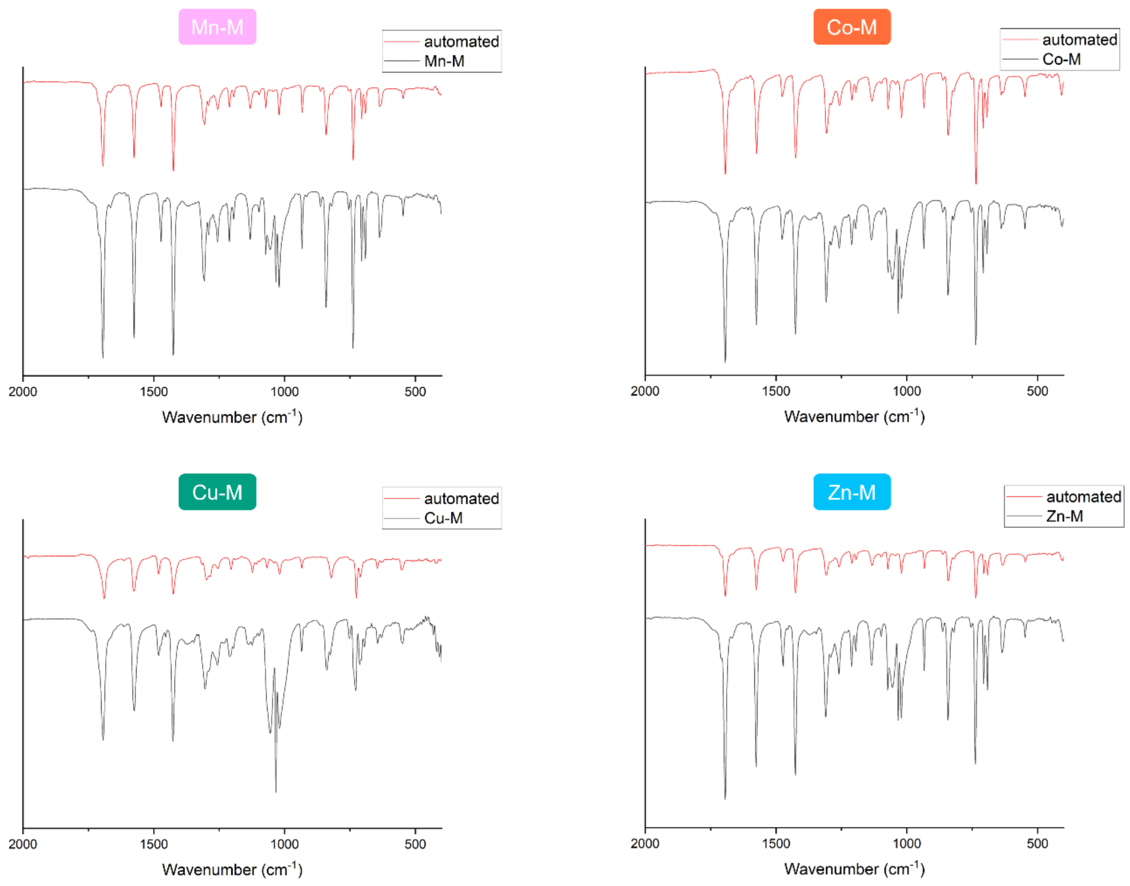

Figure S24 | FTIR spectra (400–2000 cm^{-1}) of the metal monomers synthesized through the conventional method (red) and the metal monomers prepared by automated synthesis (black).

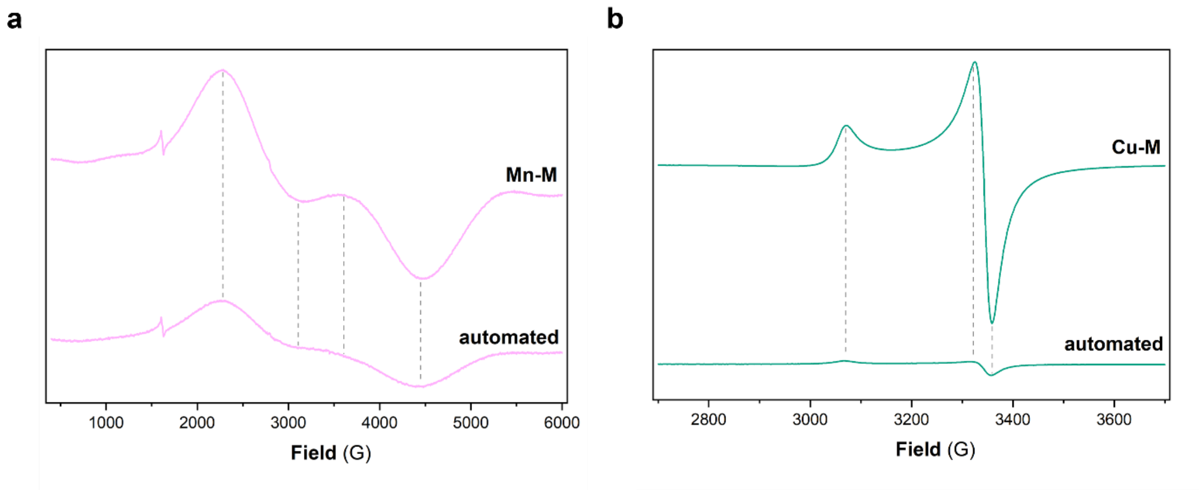

Figure S25 | X-band EPR spectra of **Mn-M** (**a**) and **Cu-M** (**b**) synthesized through the conventional method and of **Mn-M** and **Cu-M** prepared by automated synthesis.

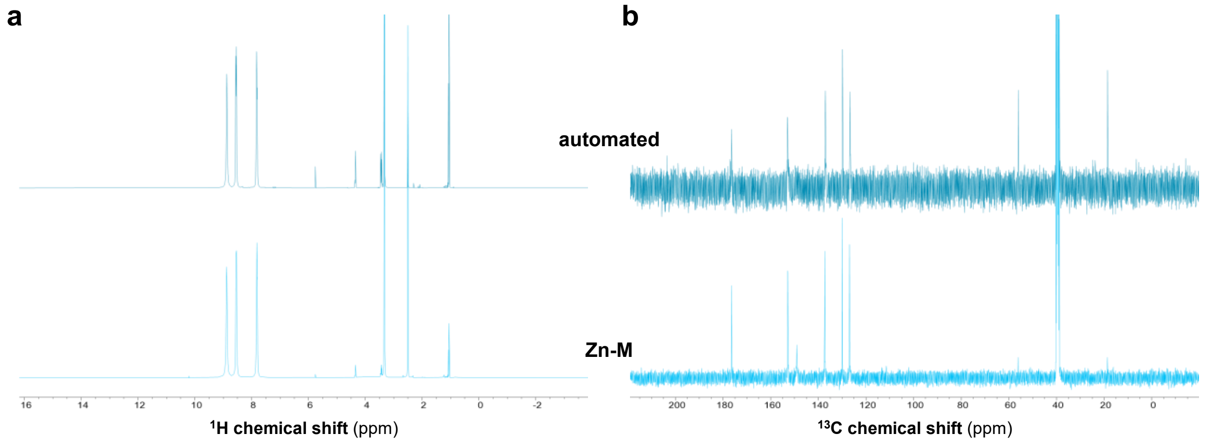

Figure S26 | ^{1}H (**a**) and ^{13}C (**b**) NMR spectra of **Zn-M** synthesized through the conventional method and **Zn-M** prepared by automated synthesis.

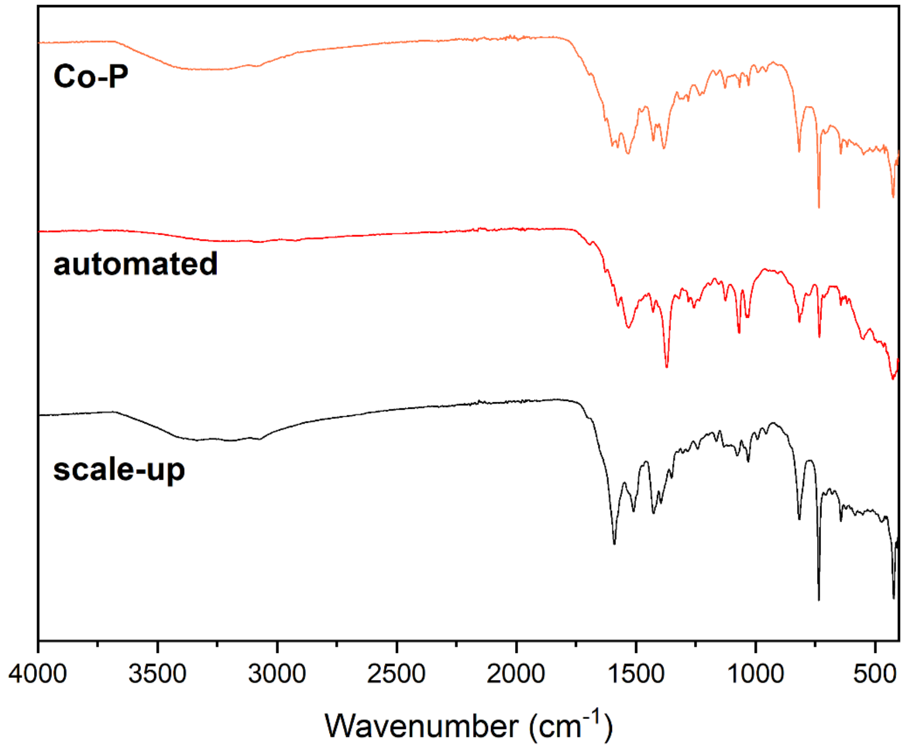

Figure S27 | FTIR spectra of **Co-P** synthesized through the conventional method (orange), automation (red), and scale-up respectively (black).

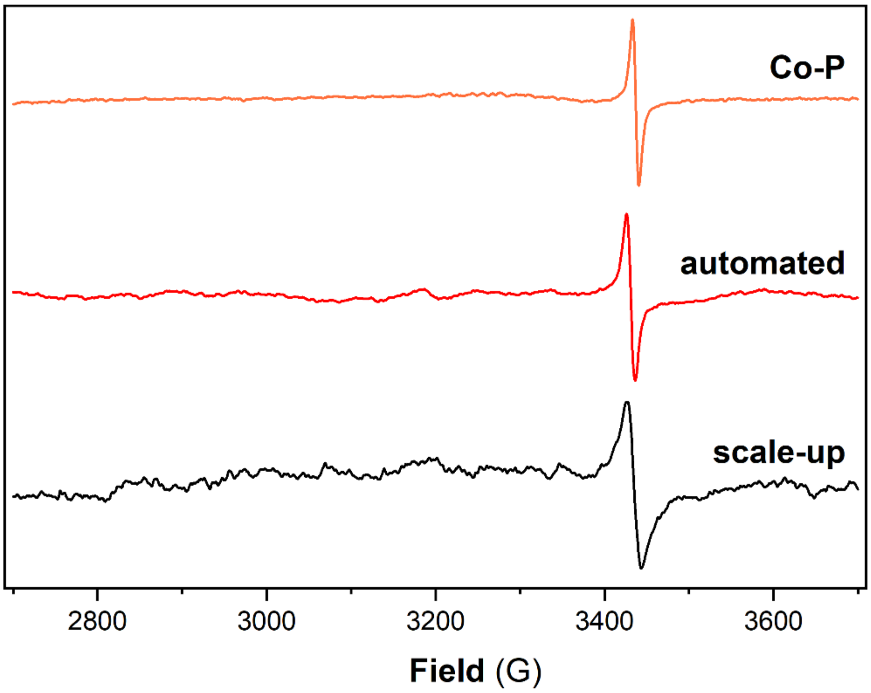

Figure S28 | X-band EPR spectra of **Co-P** synthesized through the conventional method (orange), automation (red), and scale-up respectively (black).

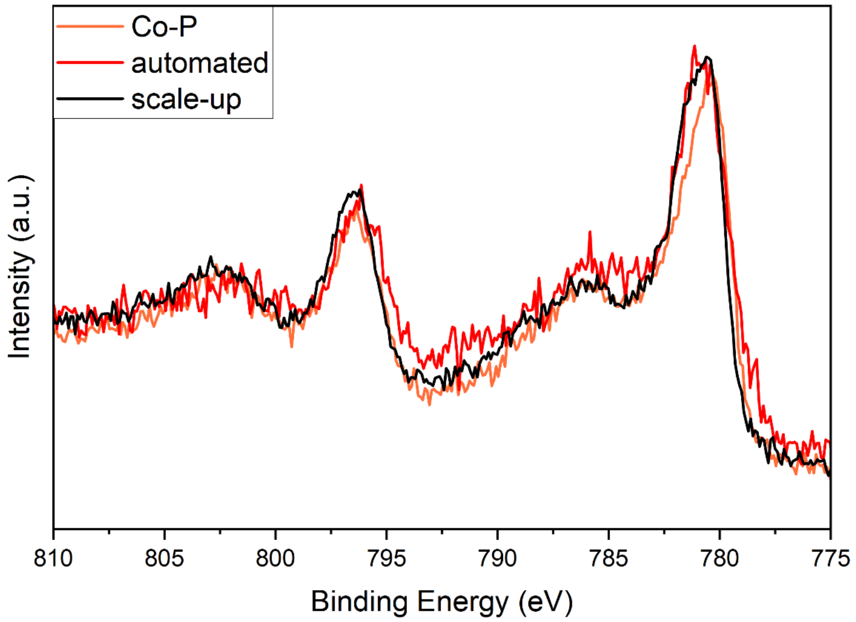

Figure S29 | XPS of **Co-P** synthesized through the conventional method (orange), automation (red), and scale-up (black).

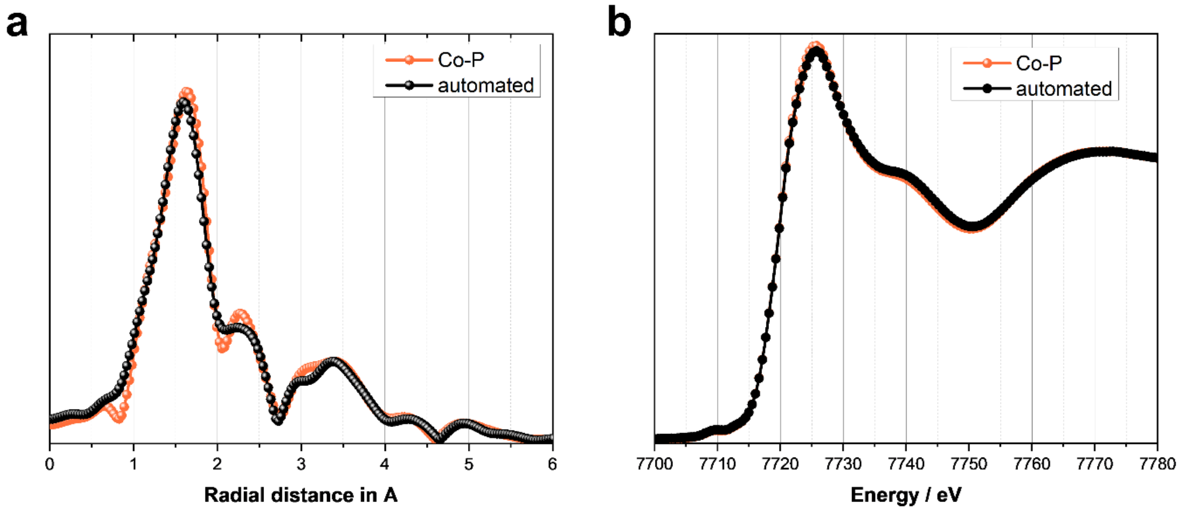

Figure S30 | Fourier transformation (not corrected for phase shift) of the Co-L edge EXAFS (**a**) and XANES (**b**) spectra of **Co-P** prepared through the conventional method and by automated synthesis.

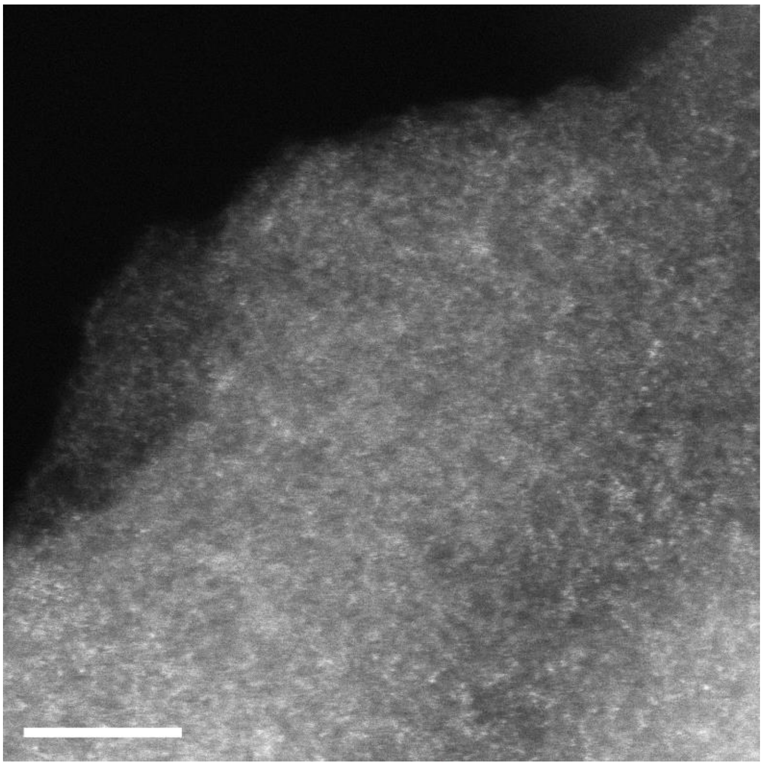

Figure S31 | HAADF-STEM image of **Co-P** by scaled-up synthesis. Scale bar 5 nm.

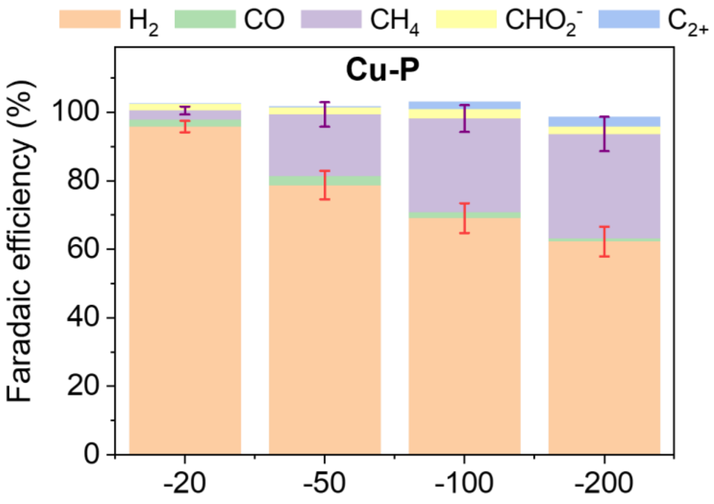

Figure S32 | Faradaic efficiency of different CO_2RR products with **Cu-P** under different current densities in 1 M KOH. Error bars are shown for the FE of H_2 and CH_4.

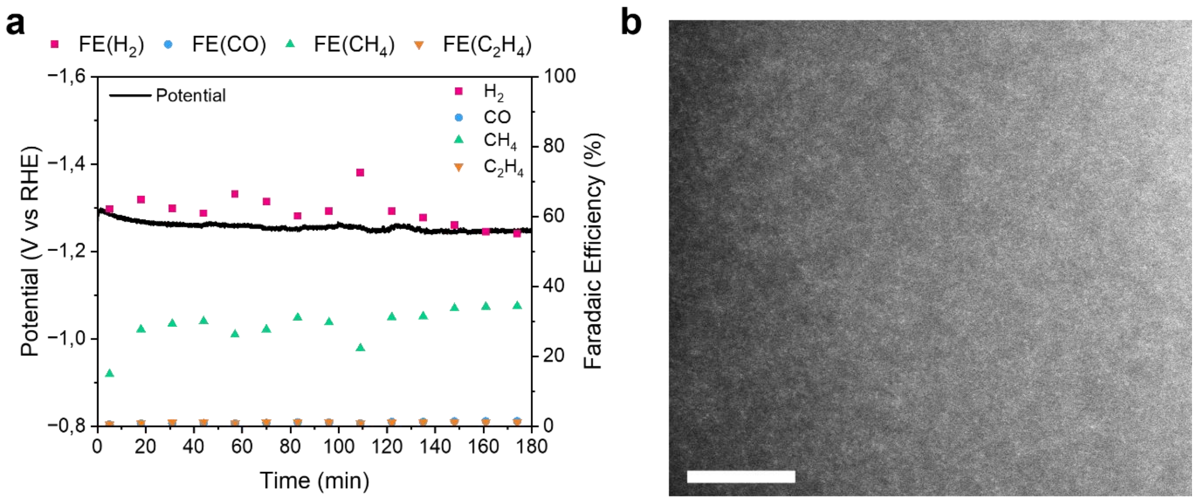

Figure S33 | Stability test of the **Cu-P** catalyst for CO_2RR at **–**200 mA cm^{-2} in 0.5 M $KHCO_3$ over 3 hours. HR-STEM image of **Cu-P** after the reaction (**b**). Scale bar 5 nm.

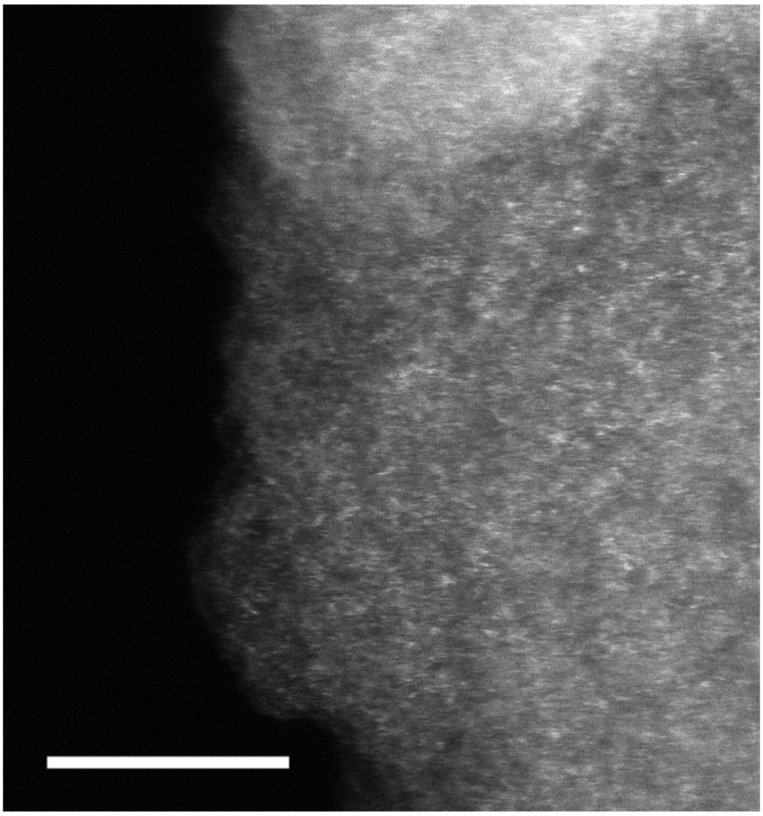

Figure S34 | HR-STEM image of **Co-P** after 100 hours continuous operation for the HER. Scale bar 5 nm.

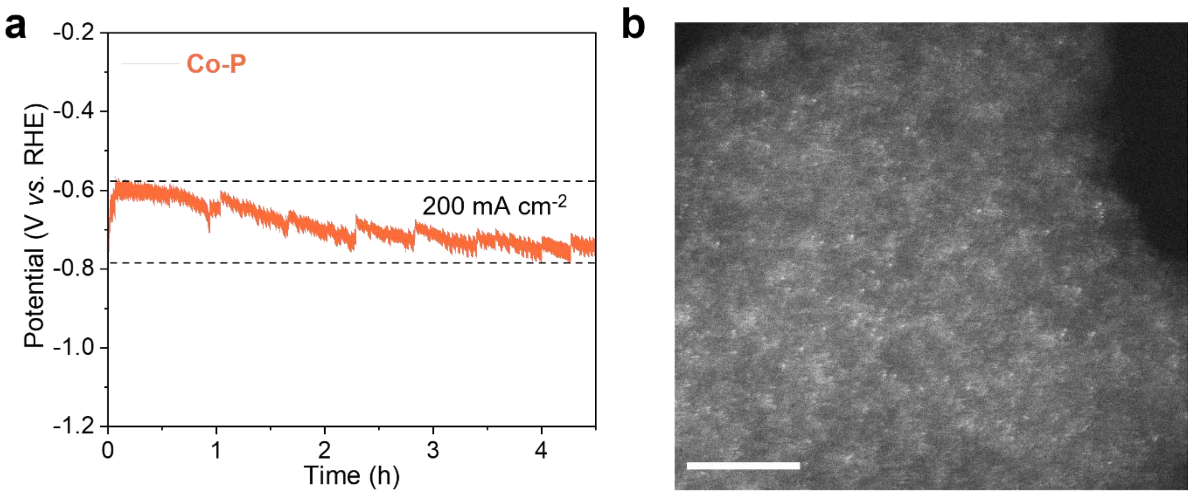

Figure S35 | Stability test of the **Co-P** catalyst for HER at −200 mA cm^{-2} over 5 hours (**a**). Potential shown has been iR corrected due to changes in resistance during measurement. At high current density of −200 mA cm^{-2}, the catalyst partially detaches from the glassy carbon electrode due to gas bubble formation, resulting in a gradual decline in performance (potential) over prolonged operation. HR-STEM image of **Co-P** after the reaction (**b**). Scale bar 5 nm.

Table S4 | Comparison data of Pd catalysts used in photocatalytic water splitting HER and of different SACs used in photocatalytic water splitting HER without the use of a sacrificial agent.*

Catalyst (Material/Metal)	Light source	Metal loading (wt %)	Activity (HER) (μmol g^{-1} h^{-1})	Solution/sacrificial agent used
**Pd-P (this work)**	**390 nm (10 W)**	**14.9**	**256**	**Pure water**
Pd_1-gCN[38]	300 W Xe lamp ($\lambda > 400$ nm)	0.1	728	20 vol% triethanolamine
Pd-TiO_2[39]	BLB near-UV lamp	0.25	936	2.0 v/v% ethanol solution, pH= 4 (H_2SO_4)
Pd/IrO_2-P10 Polymer[40]	300 W Xe lamp ($\lambda > 420$ nm)	0.45	5.6	Pure water
Co_1-phosphide/PCN[41]	300 W Xe lamp ($\lambda > 300$ nm)	0.4	410.3	Pure water
Pt_1–Au_1/C_3N_4[42]	300 W Xe lamp ($\lambda > 420$ nm)	0.4	285	Pure water
Ni_1-PCN[43]	300 W Xe lamp ($\lambda > 420$ nm)	1	26.6	Pure water

***Note:** Performing the reaction in the absence of light, water (toluene used as solvent) or catalyst yields no hydrogen production.

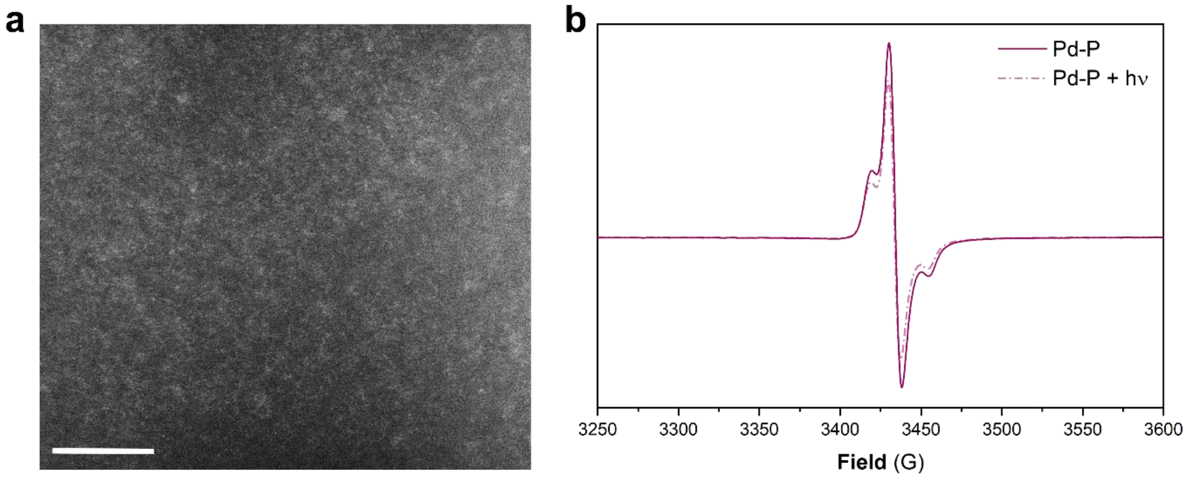

Figure S36 | HR-STEM image (**a**) and EPR spectra (**b**) of **Pd-P** after continuous operation for the OWS. Scale bar 5 nm.

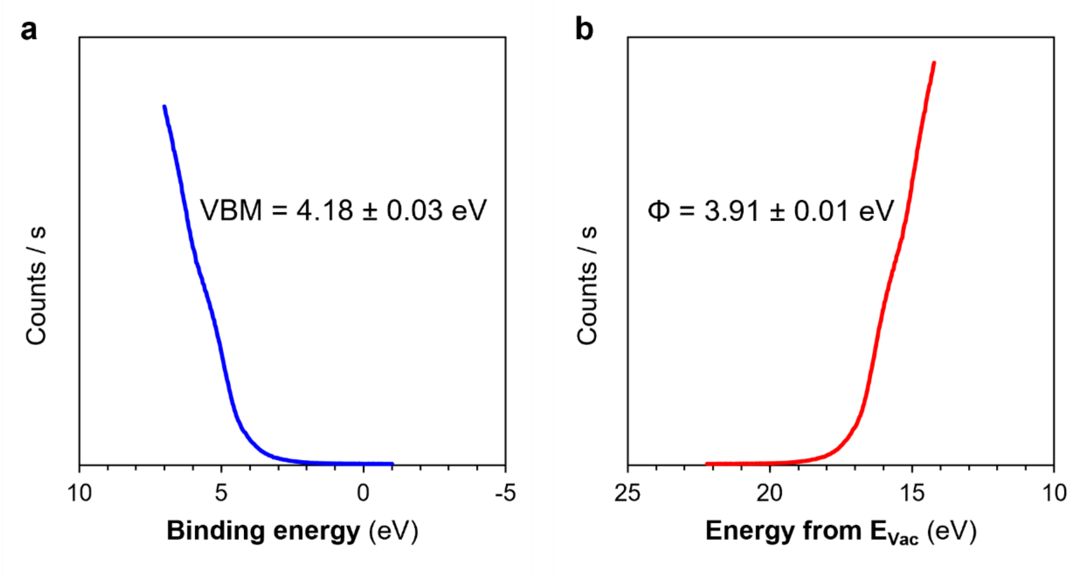

Figure S37 | UPS spectra of **Pd-P**. Valence band maxima (VBM) (**a**), and work function (Φ) (**b**).

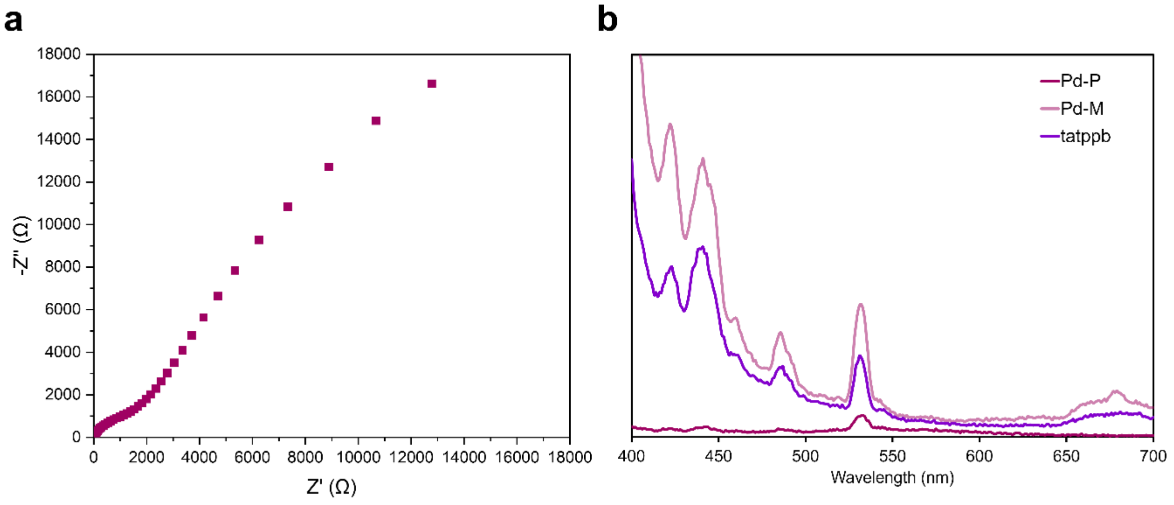

Figure S38 | EIS measurement of **Pd-P** (**a**), and PL spectra of **Pd-P**, **Pd-M** and **tatppb** films (**b**).

Supporting spectra

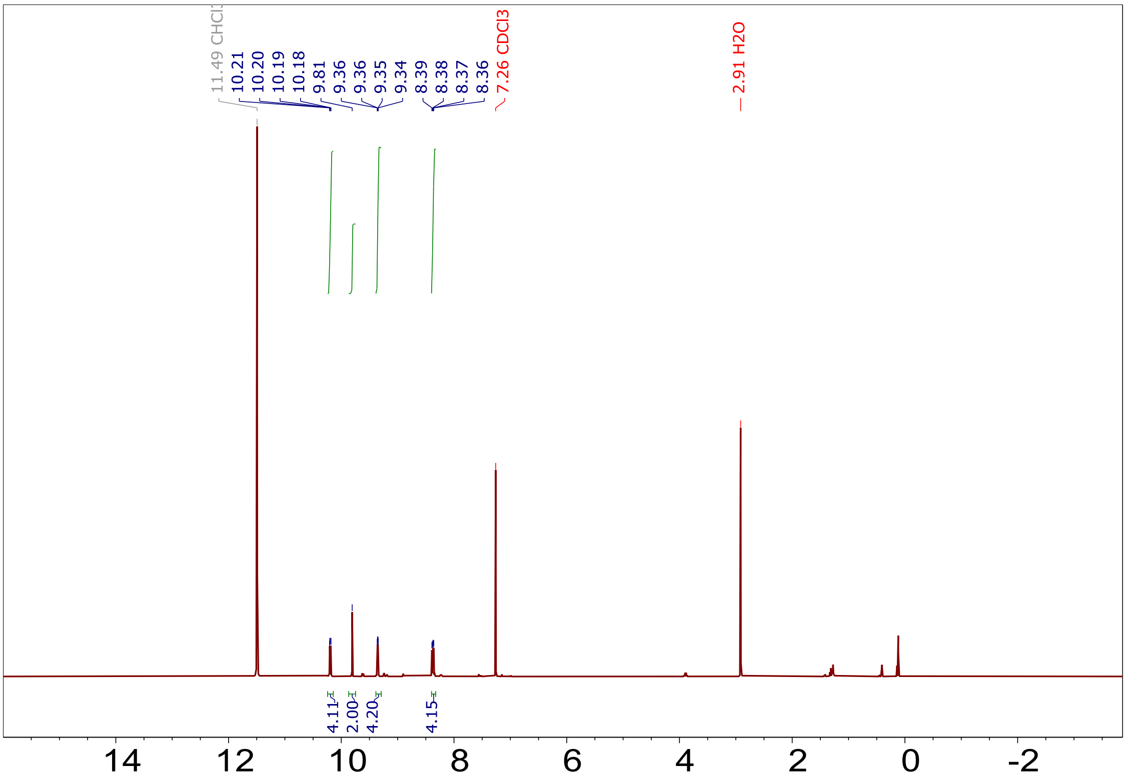

Figure S39 | ^{1}H NMR spectrum of **tatppb**. Residual peaks of *d*-TFA are labeled grey in the spectrum.

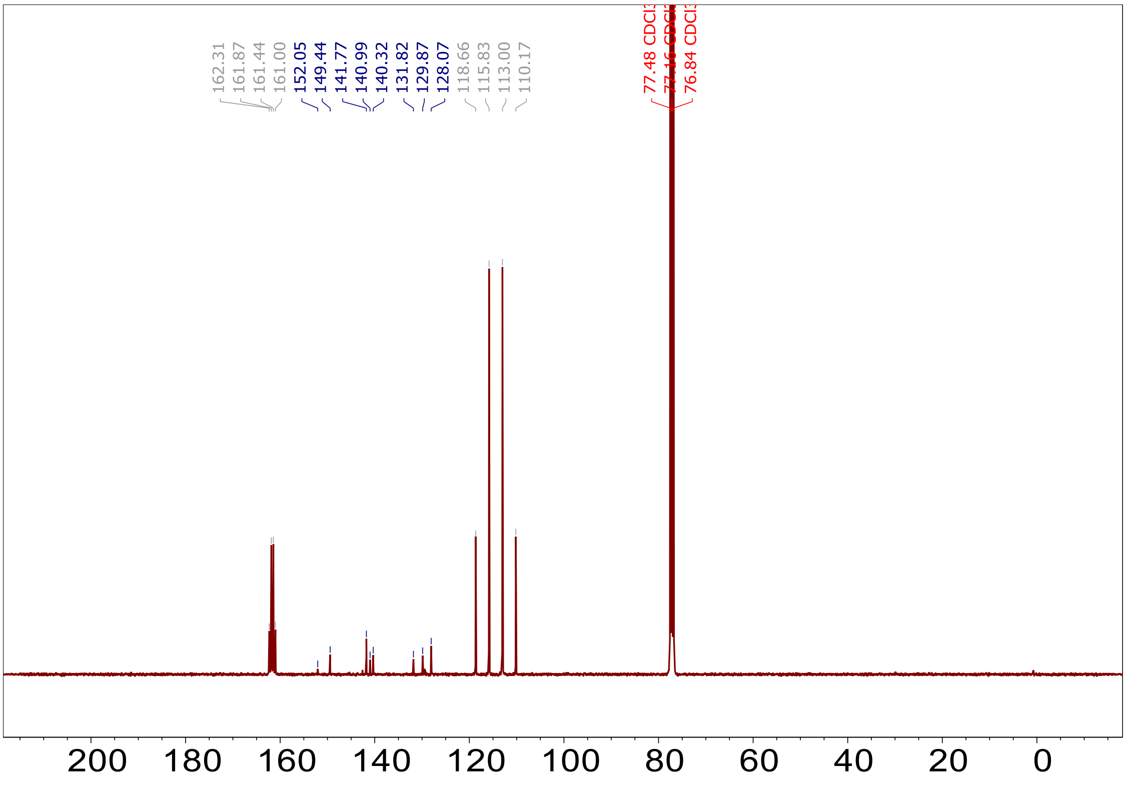

Figure S40 | ^{13}C NMR spectrum of **tatppb**. Residual peaks of *d*-TFA are labeled grey in the spectrum.

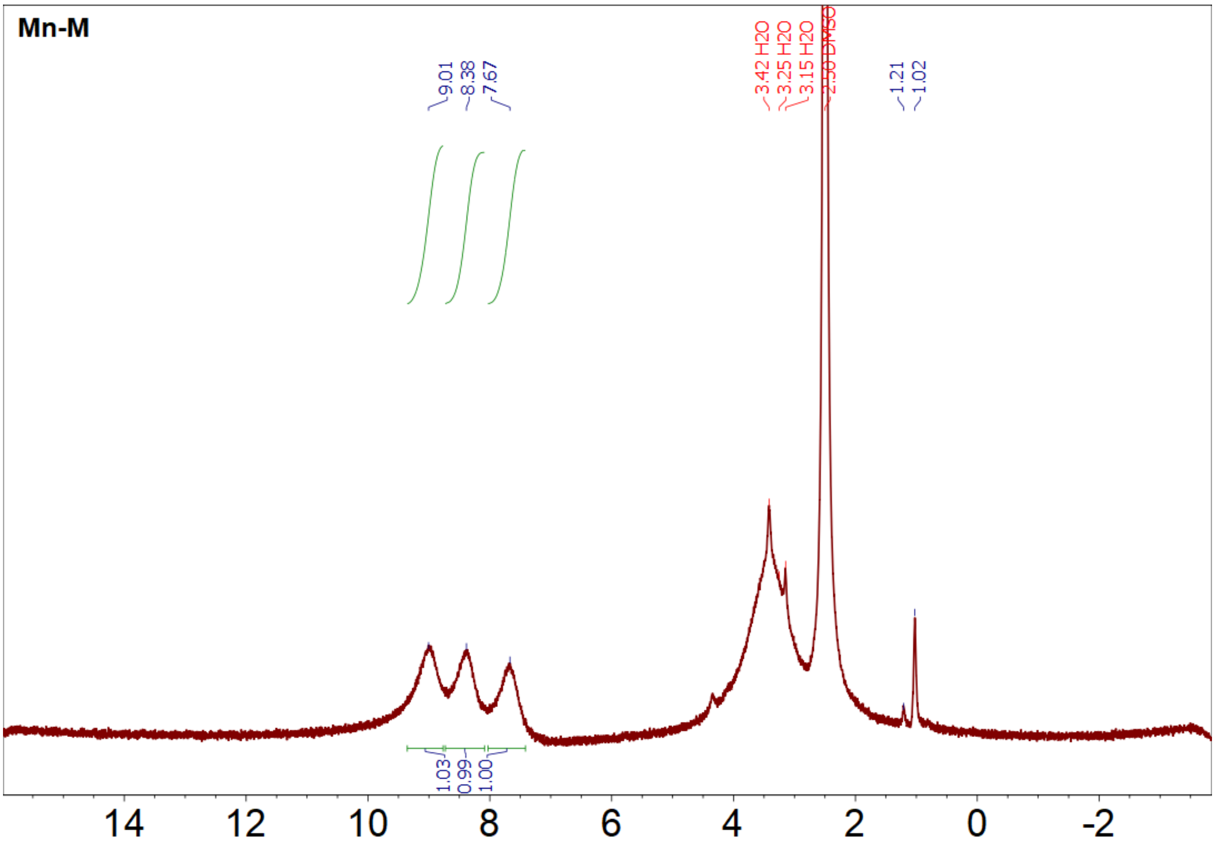

Figure S41 | ^{1}H NMR spectrum (narrow spectral width) of paramagnetic **Mn-M**.

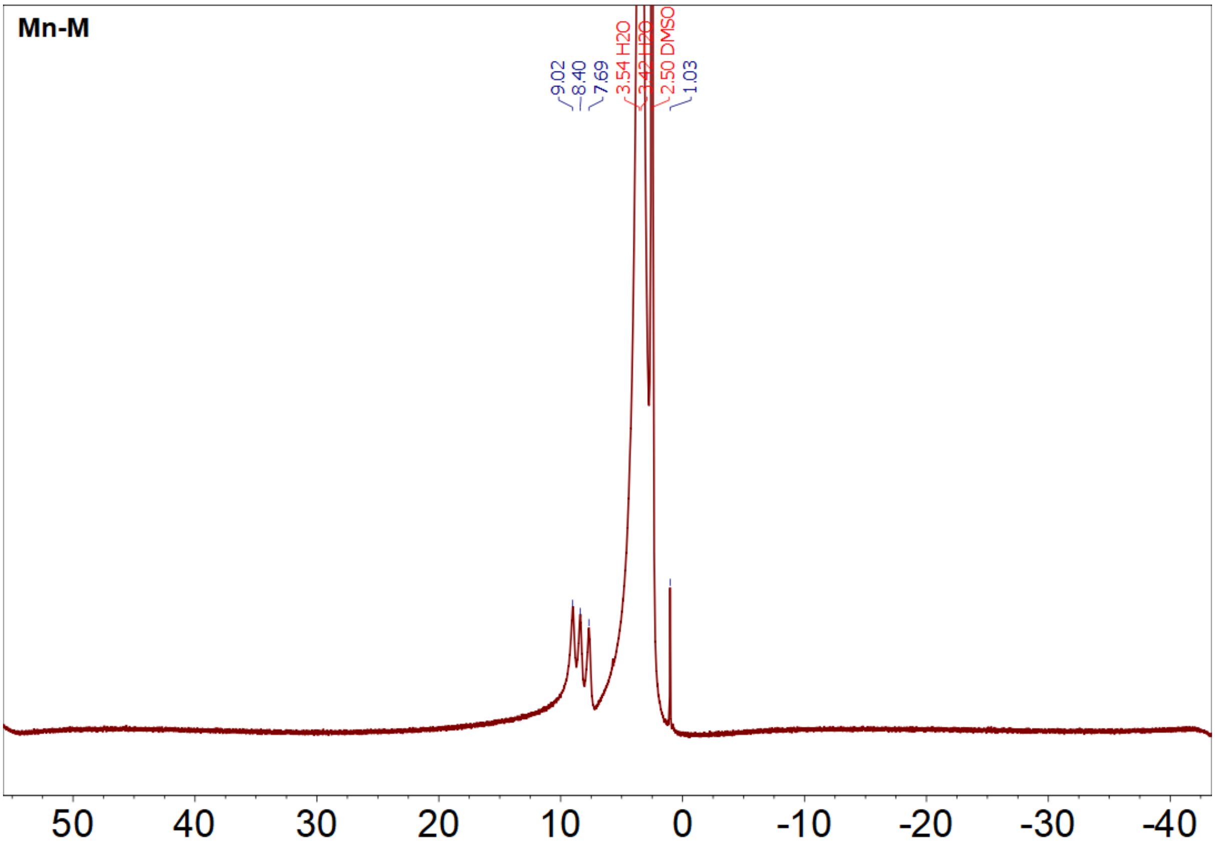

Figure S42 | ^{1}H NMR spectrum (wide spectral width) of paramagnetic **Mn-M**.

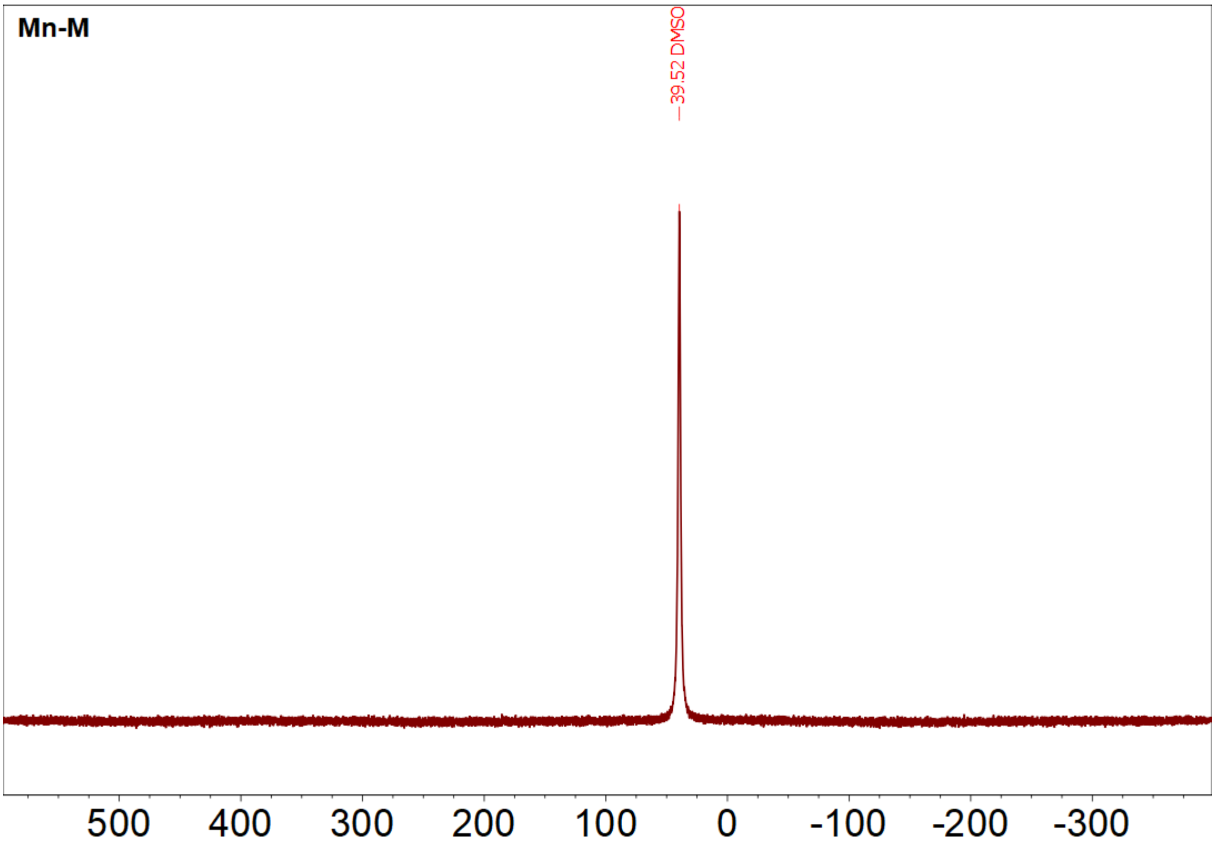

Figure S43 | ^{13}C NMR spectrum of paramagnetic **Mn-M**.

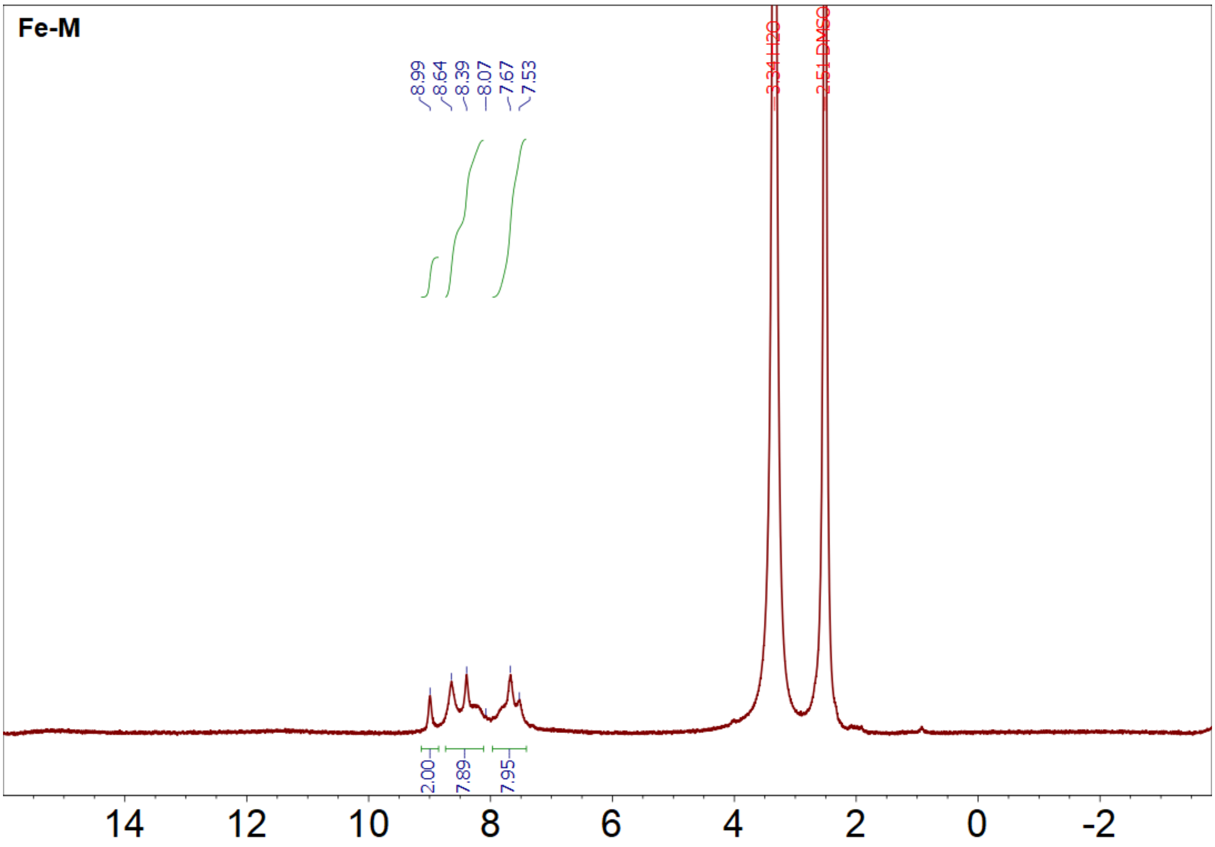

Figure S44 | ^{1}H NMR spectrum (narrow spectral width) of paramagnetic **Fe-M**.

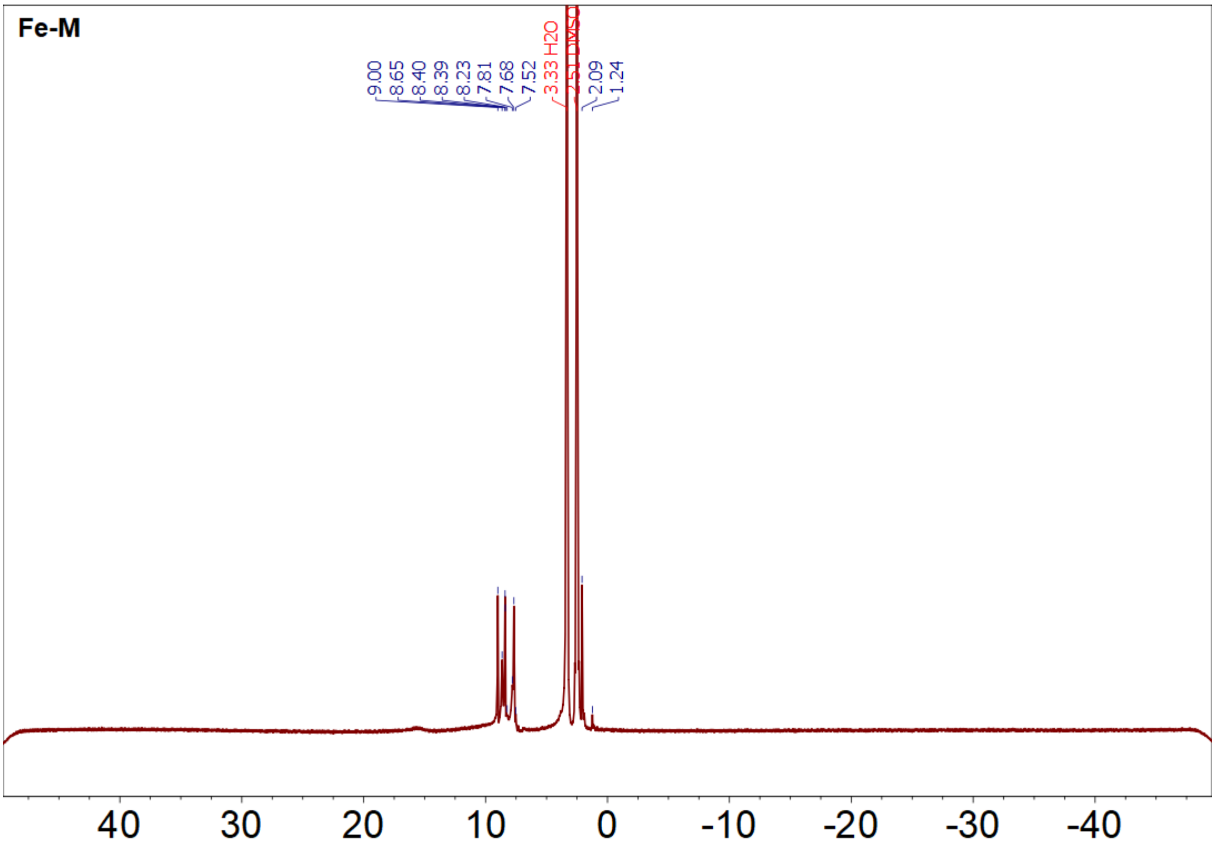

Figure S45 | ^{1}H NMR spectrum (wide spectral width) of paramagnetic **Fe-M**

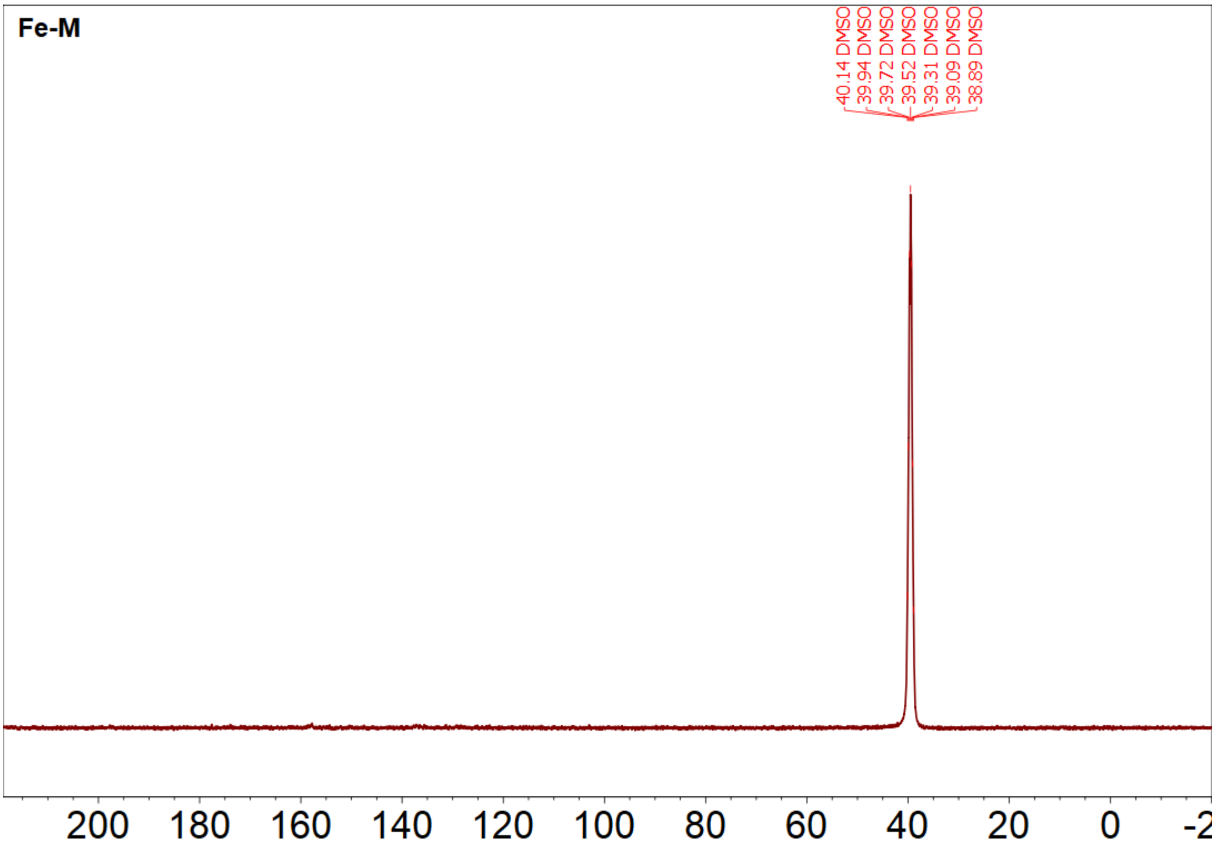

Figure S46 | ^{13}C NMR spectrum of paramagnetic **Fe-M**.

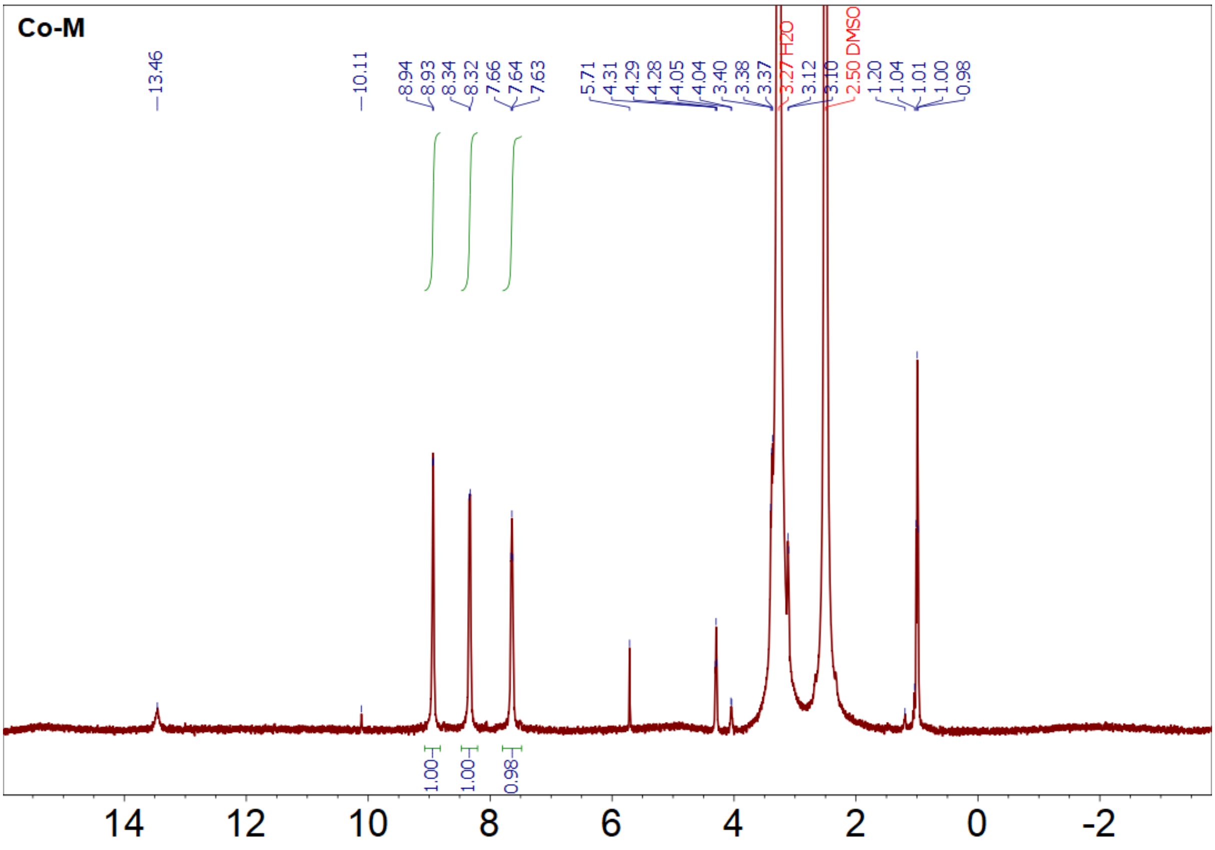

Figure S47 | ^{1}H NMR spectrum (narrow spectral width) of paramagnetic **Co-M**.

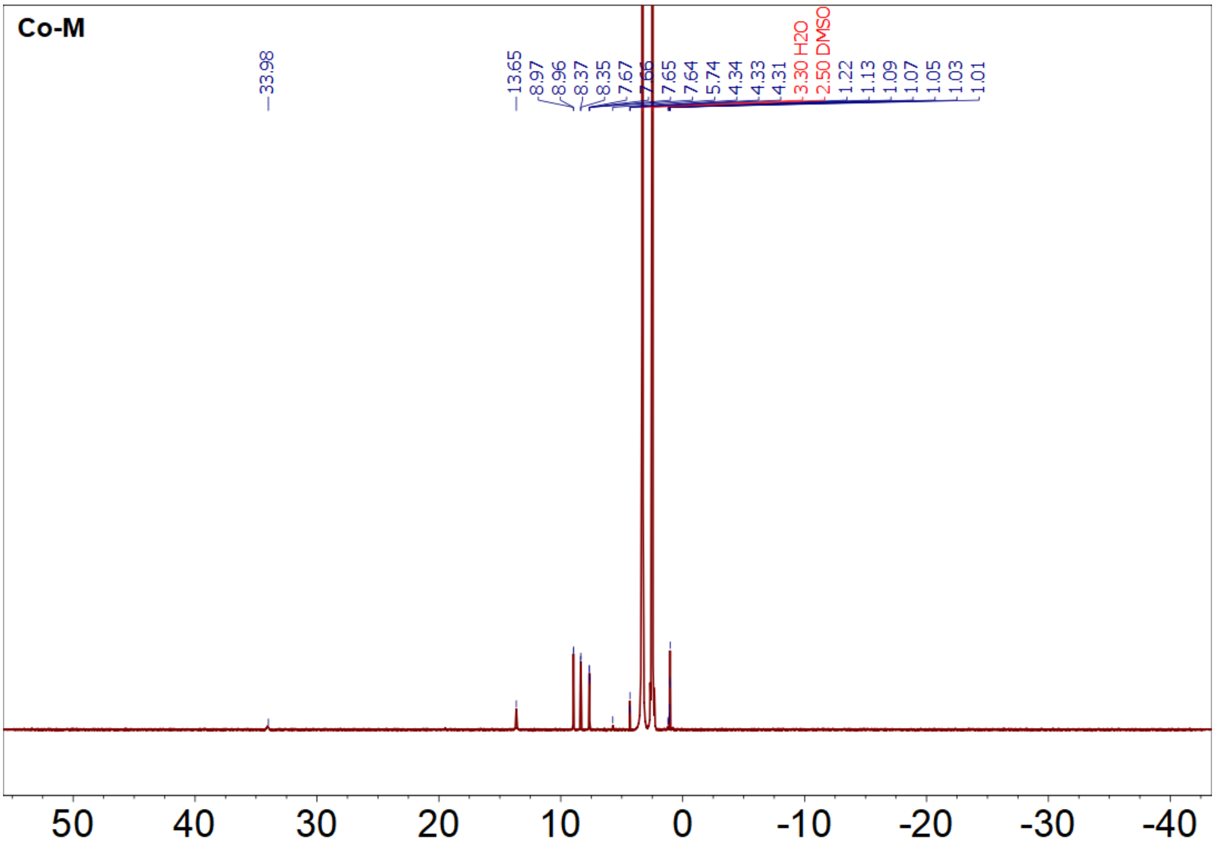

Figure S48 | ^{1}H NMR spectrum (wide spectral width) of paramagnetic **Co-M**.

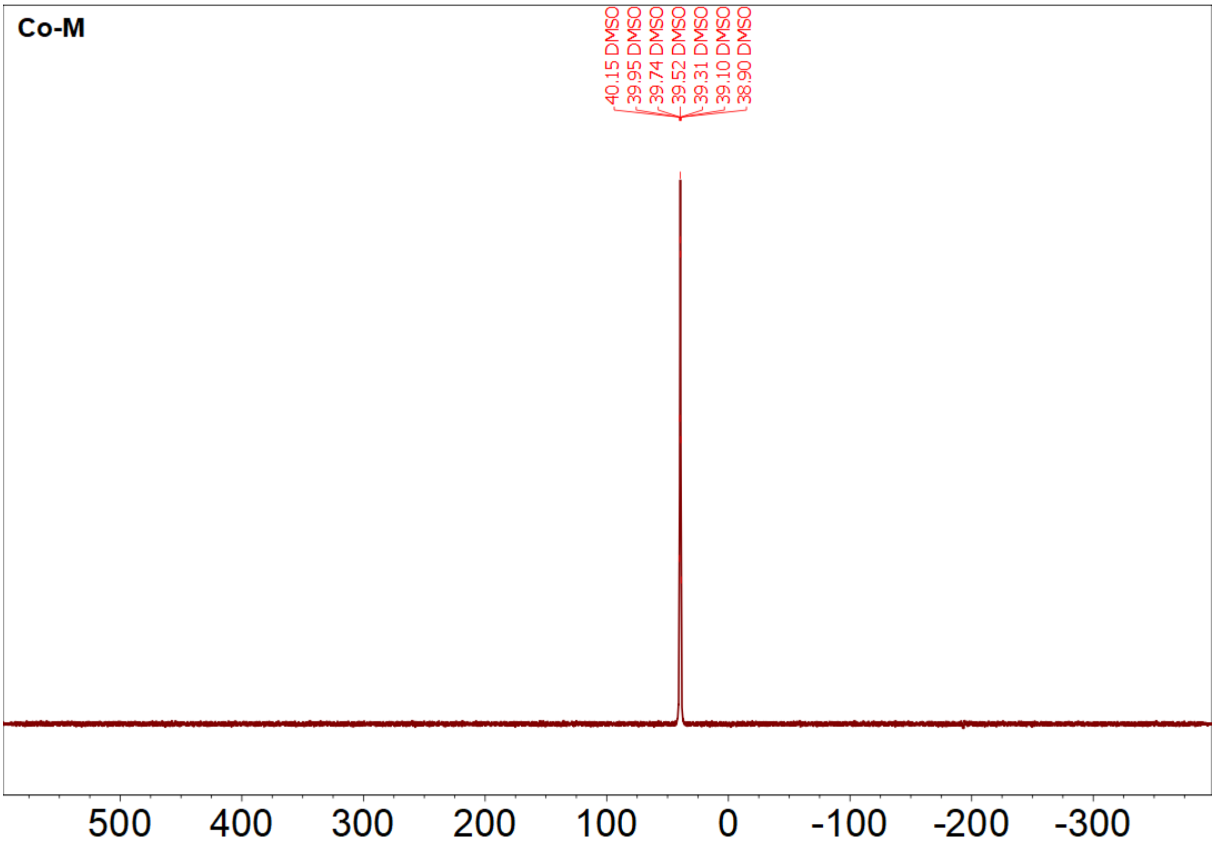

Figure S49 | ^{13}C NMR spectrum of paramagnetic **Co-M**.

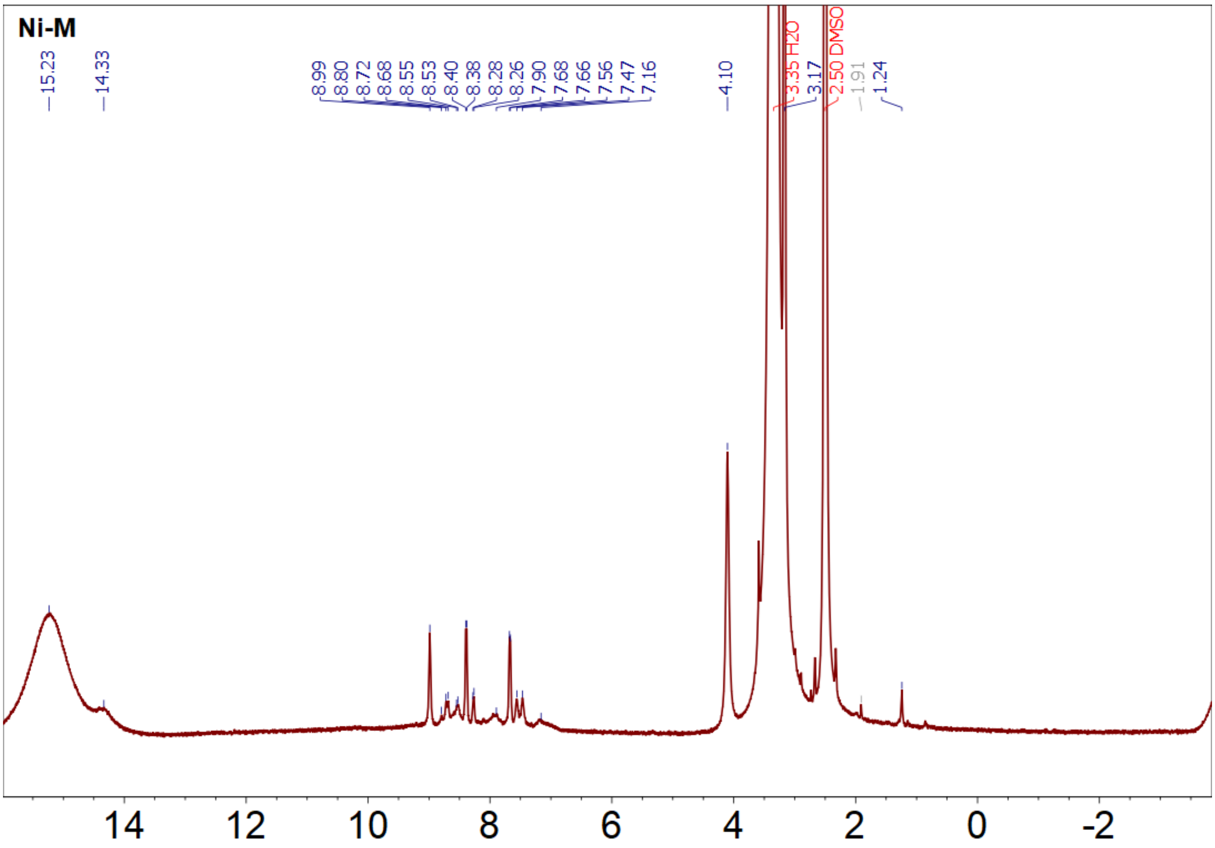

Figure S50 | ^{1}H NMR spectrum (narrow spectral width) of paramagnetic **Ni-M**.

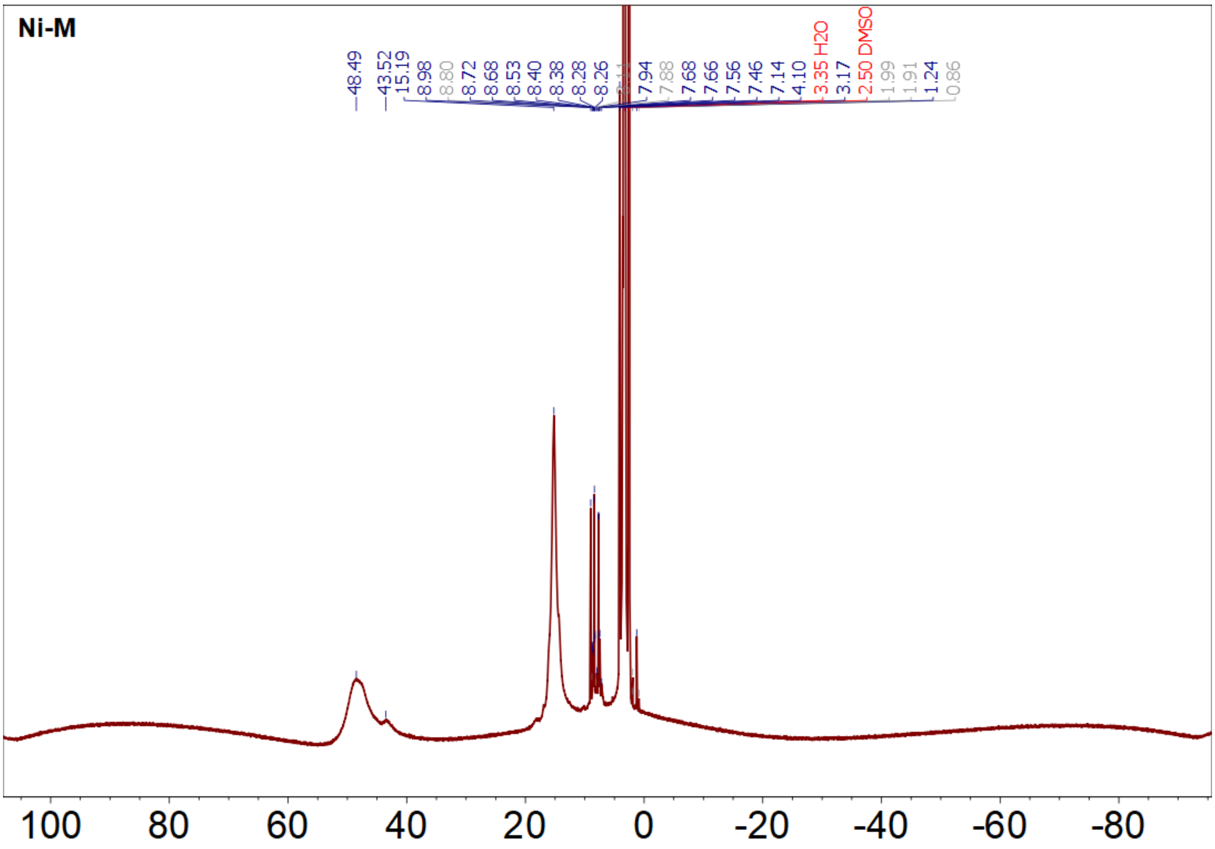

Figure S51 | ^{1}H NMR spectrum (wide spectral width) of paramagnetic **Ni-M**.

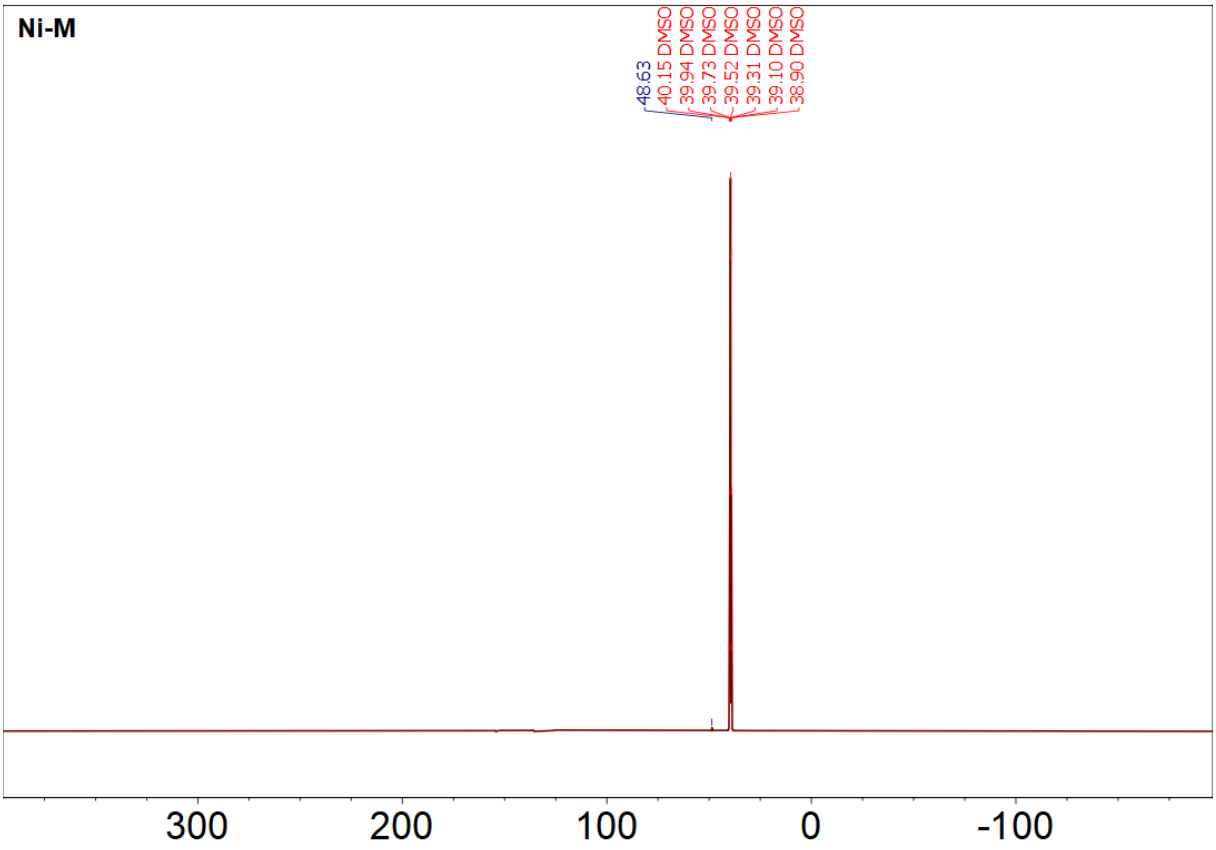

Figure S52 | ^{13}C NMR spectrum of paramagnetic **Ni-M**.

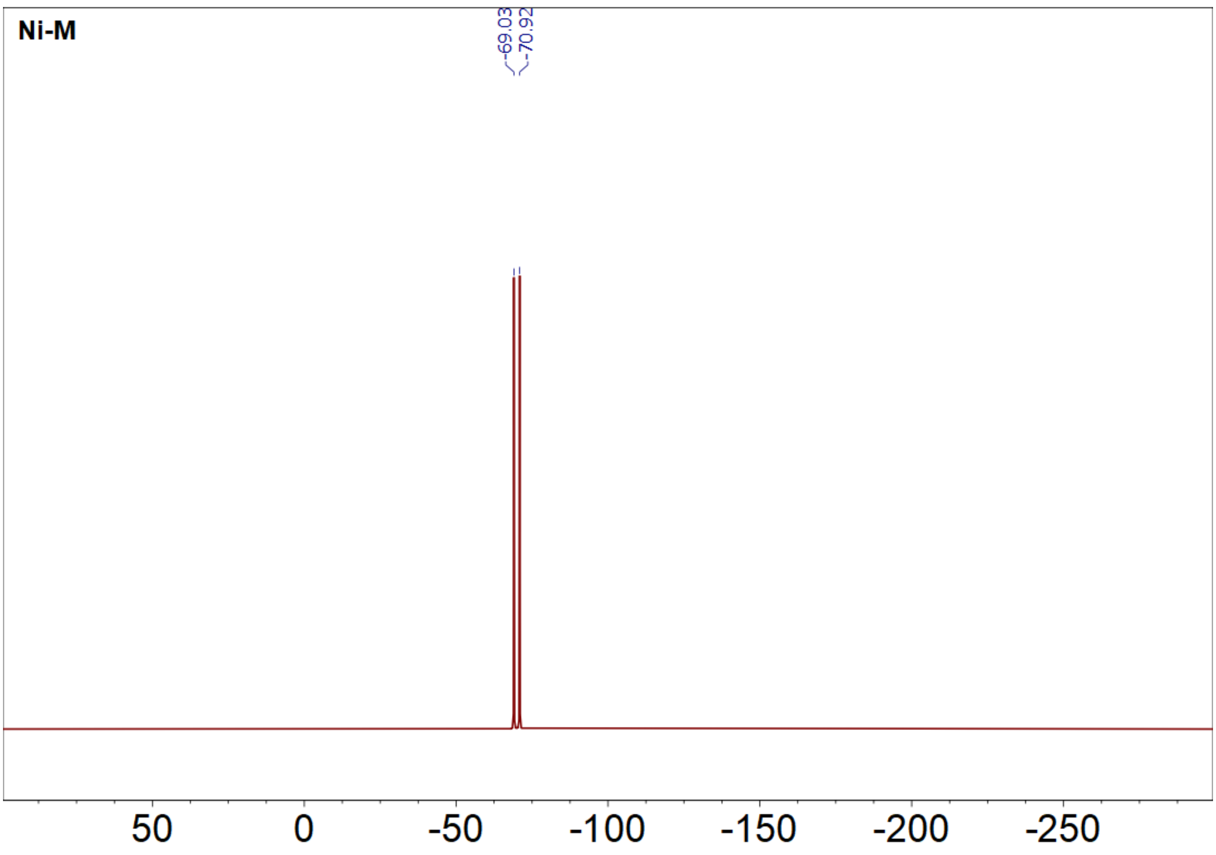

Figure S53 | ^{19}F NMR spectrum of paramagnetic **Ni-M**.

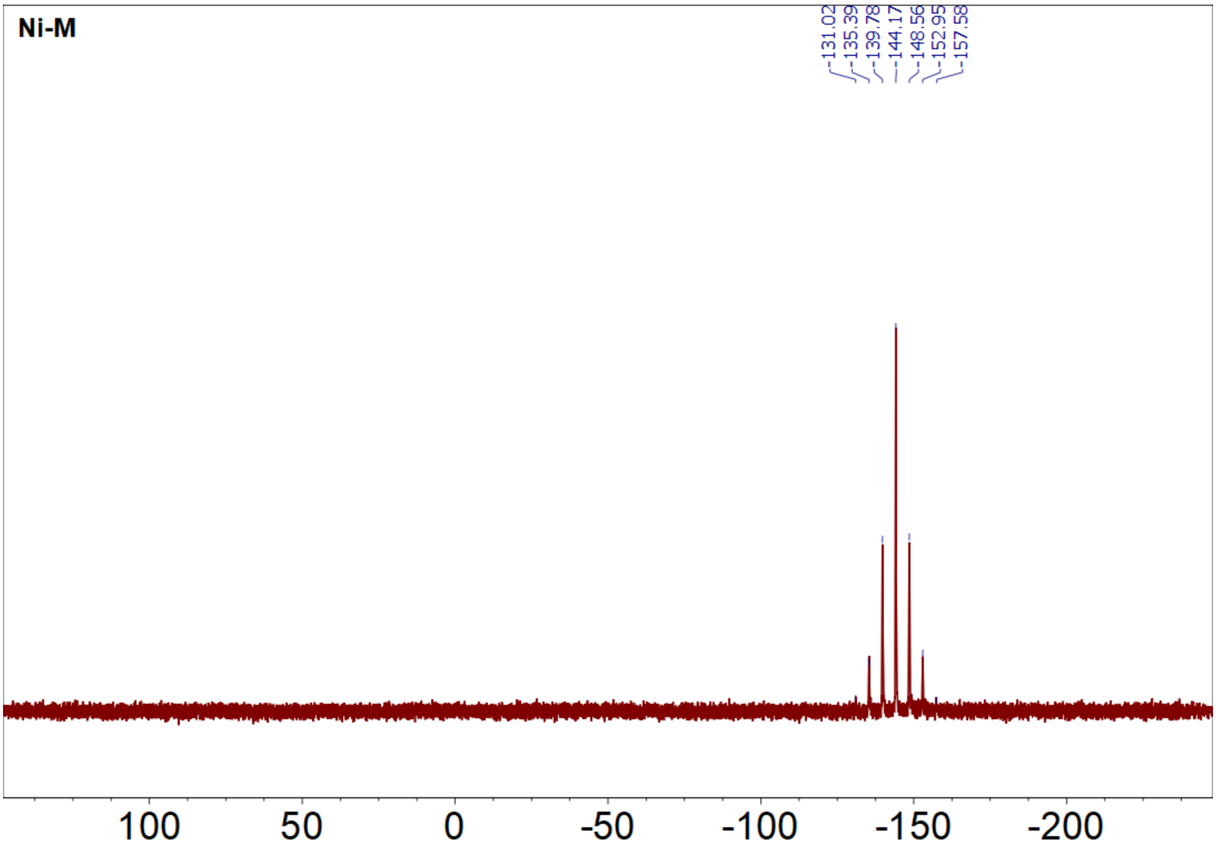

Figure S54 | ^{31}P NMR spectrum of paramagnetic **Ni-M**.

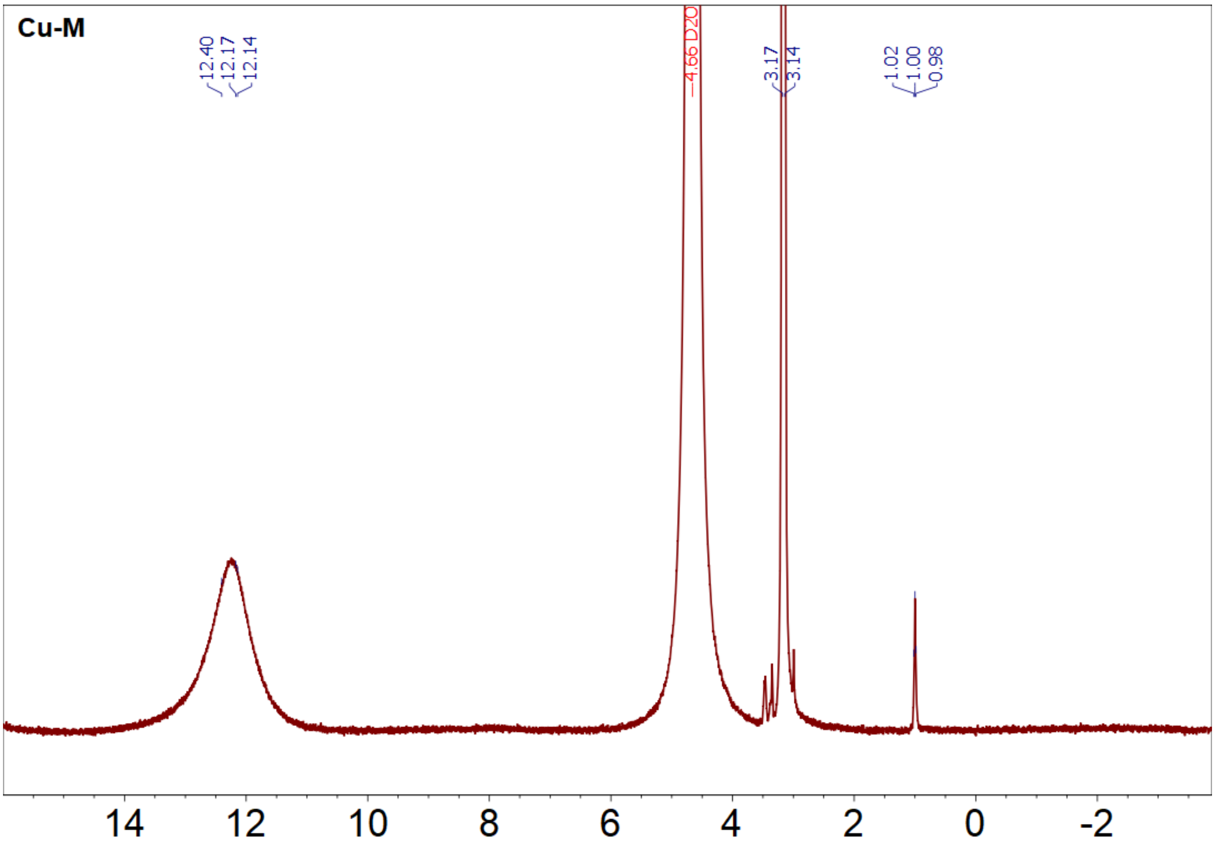

Figure S55 | ^{1}H NMR spectrum (narrow spectral width) of paramagnetic **Cu-M**.

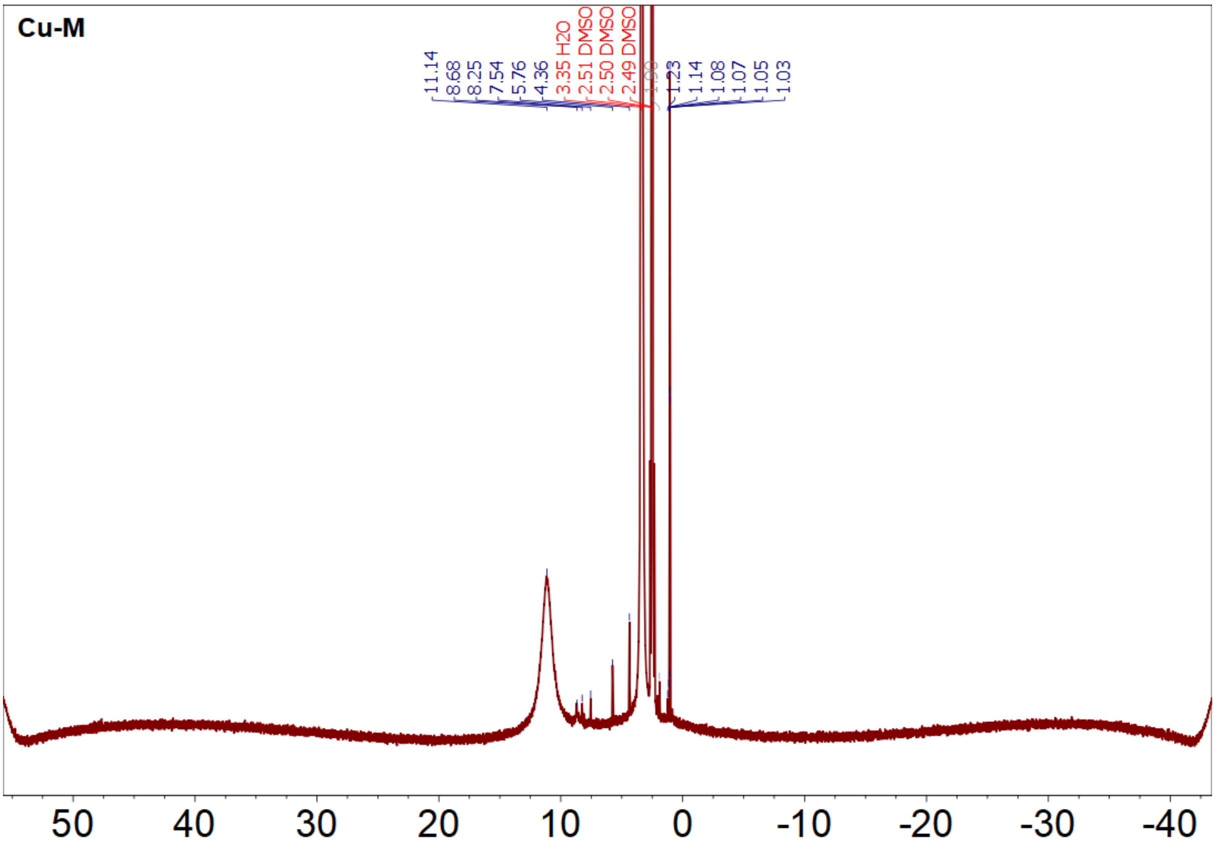

Figure S56 | ^{1}H NMR spectrum (wide spectral width) of paramagnetic **Cu-M**.

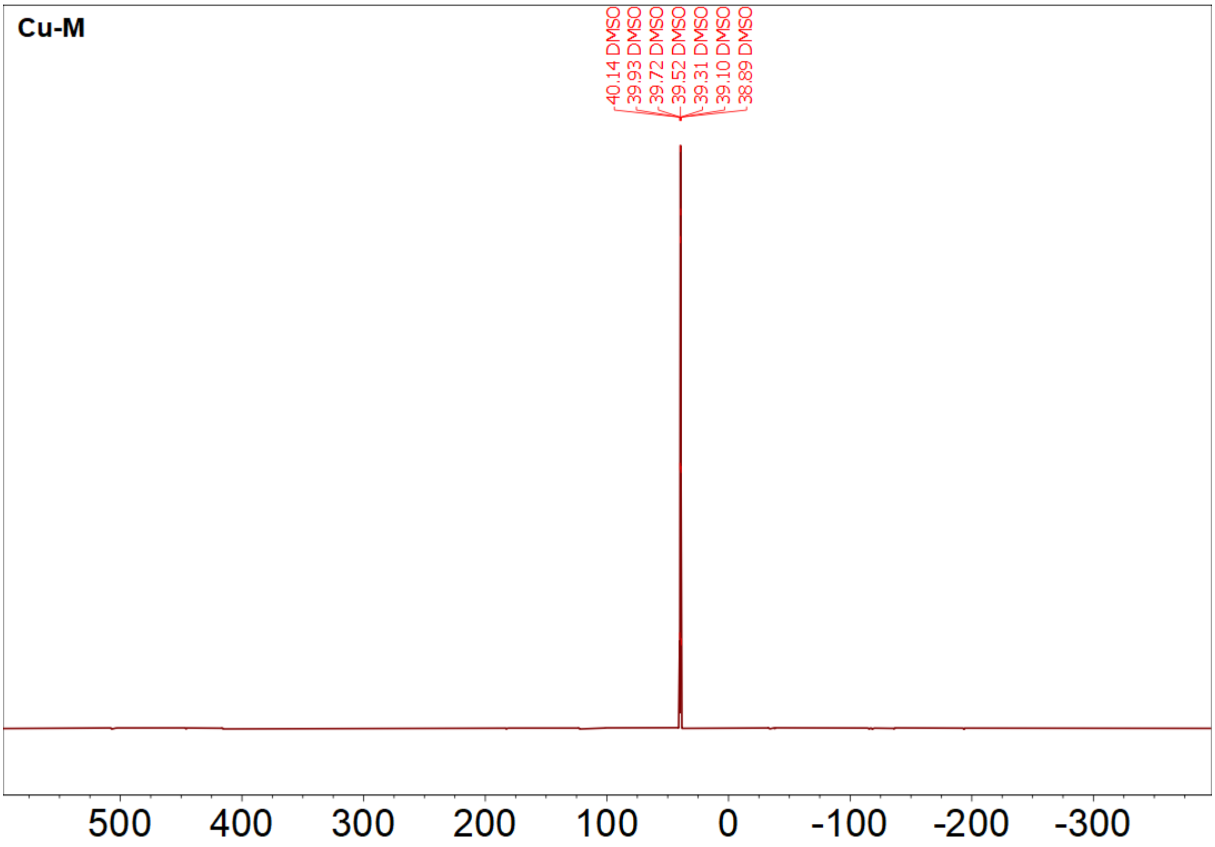

Figure S57 | ^{13}C NMR spectrum of paramagnetic **Cu-M**.

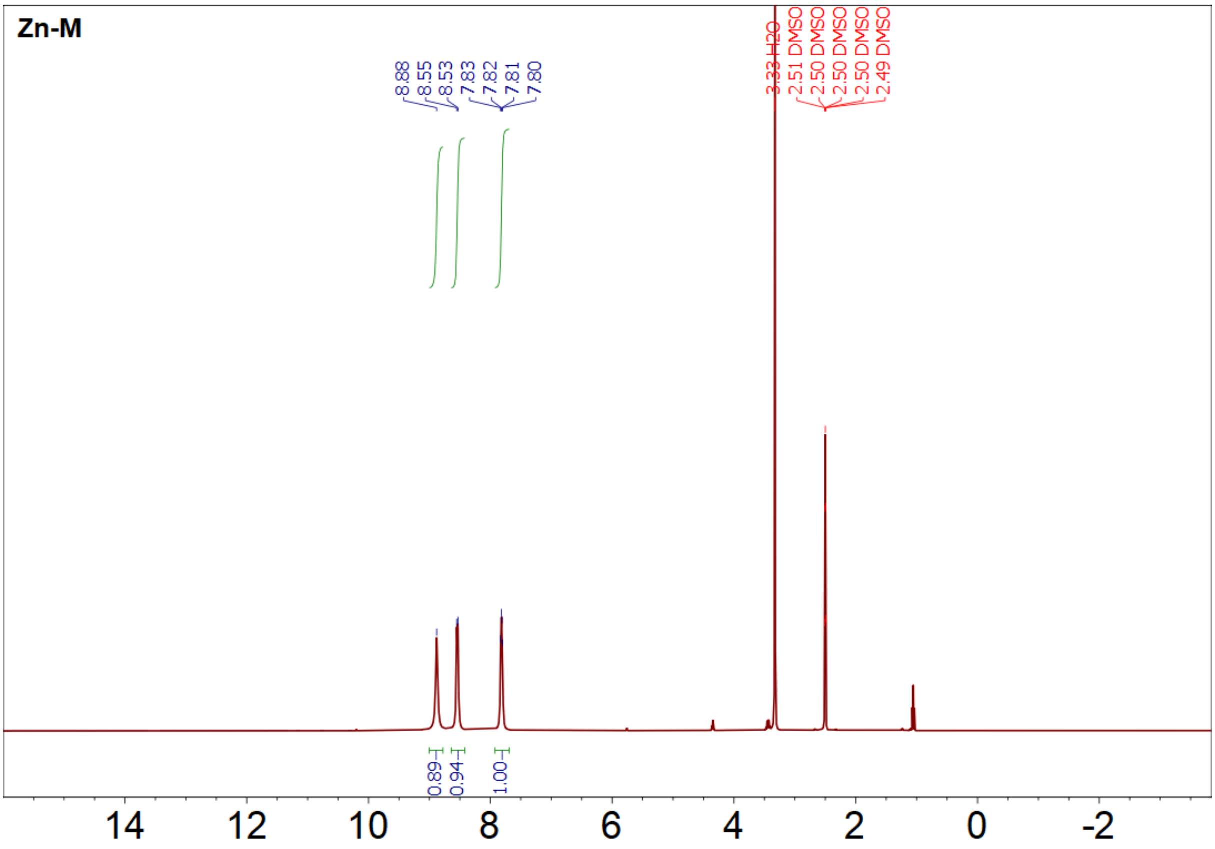

Figure S58 | ^{1}H NMR spectrum of **Zn-M**.

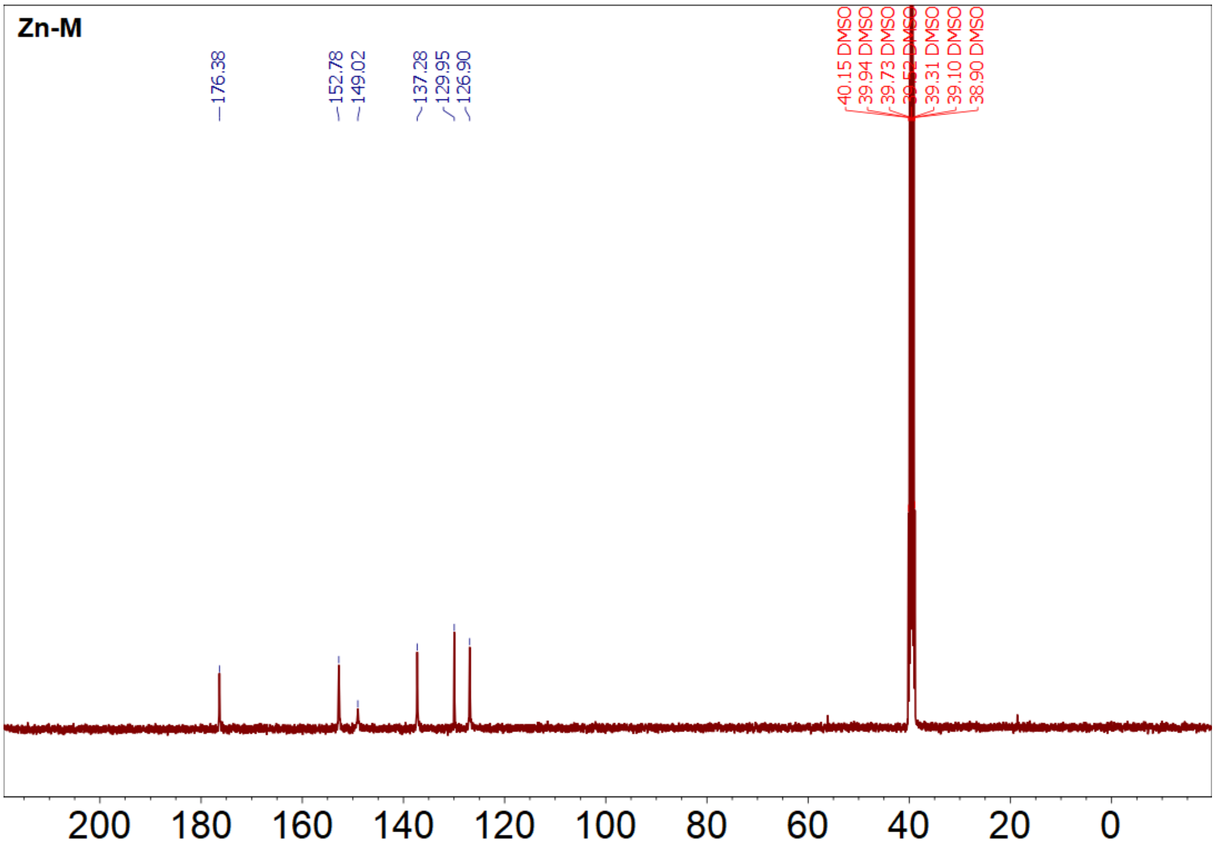

Figure S59 | ^{13}C NMR spectrum of **Zn-M**.

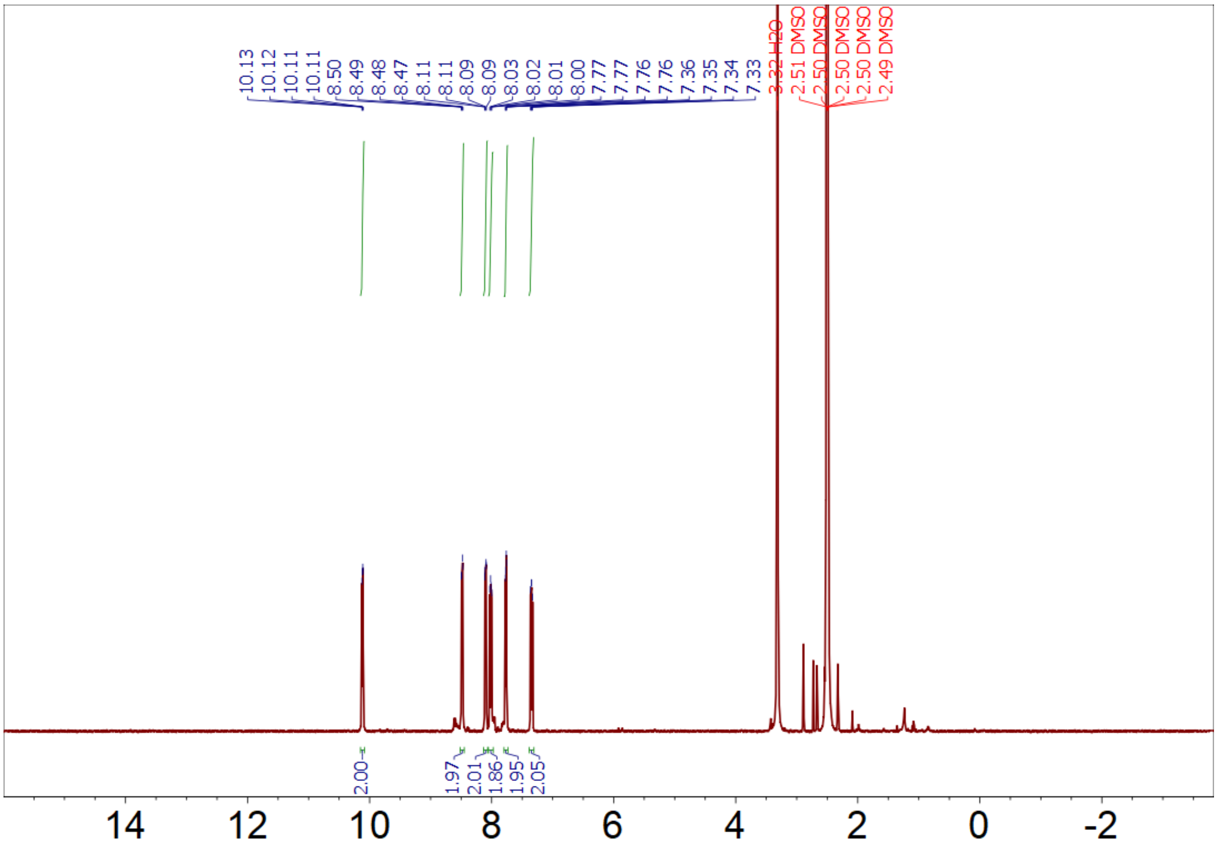

Figure S60 | ^{1}H NMR spectrum of **Ru-M**.

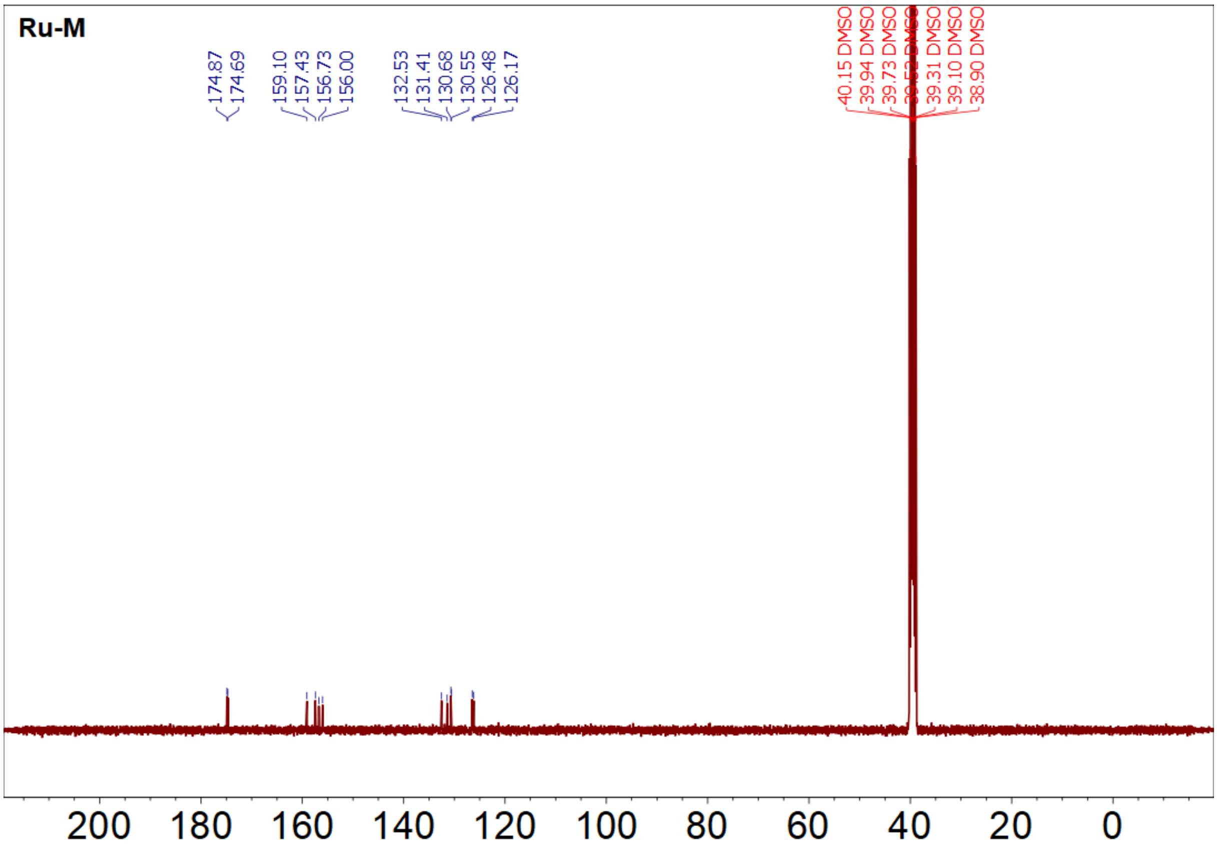

Figure S61 | ^{13}C NMR spectrum of **Ru-M**.

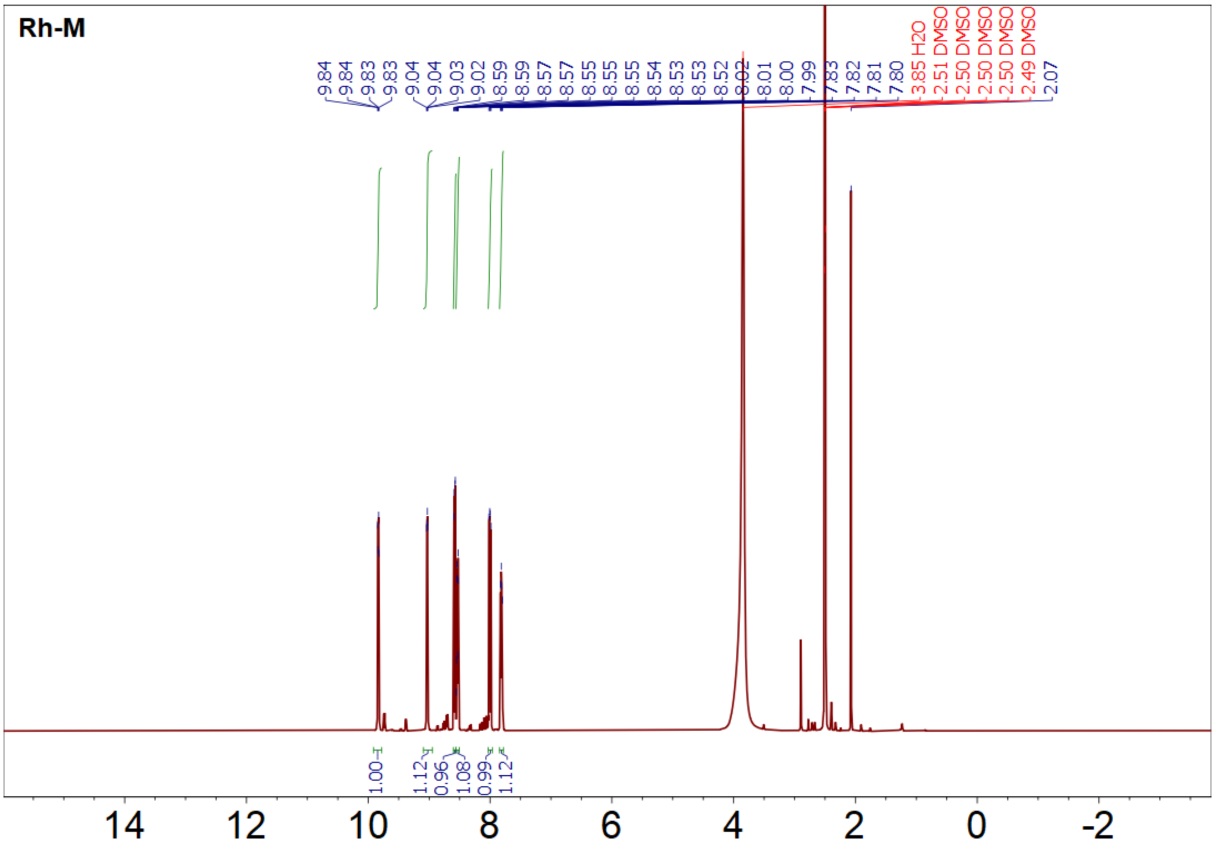

Figure S62 | ^{1}H NMR spectrum of **Rh-M**.

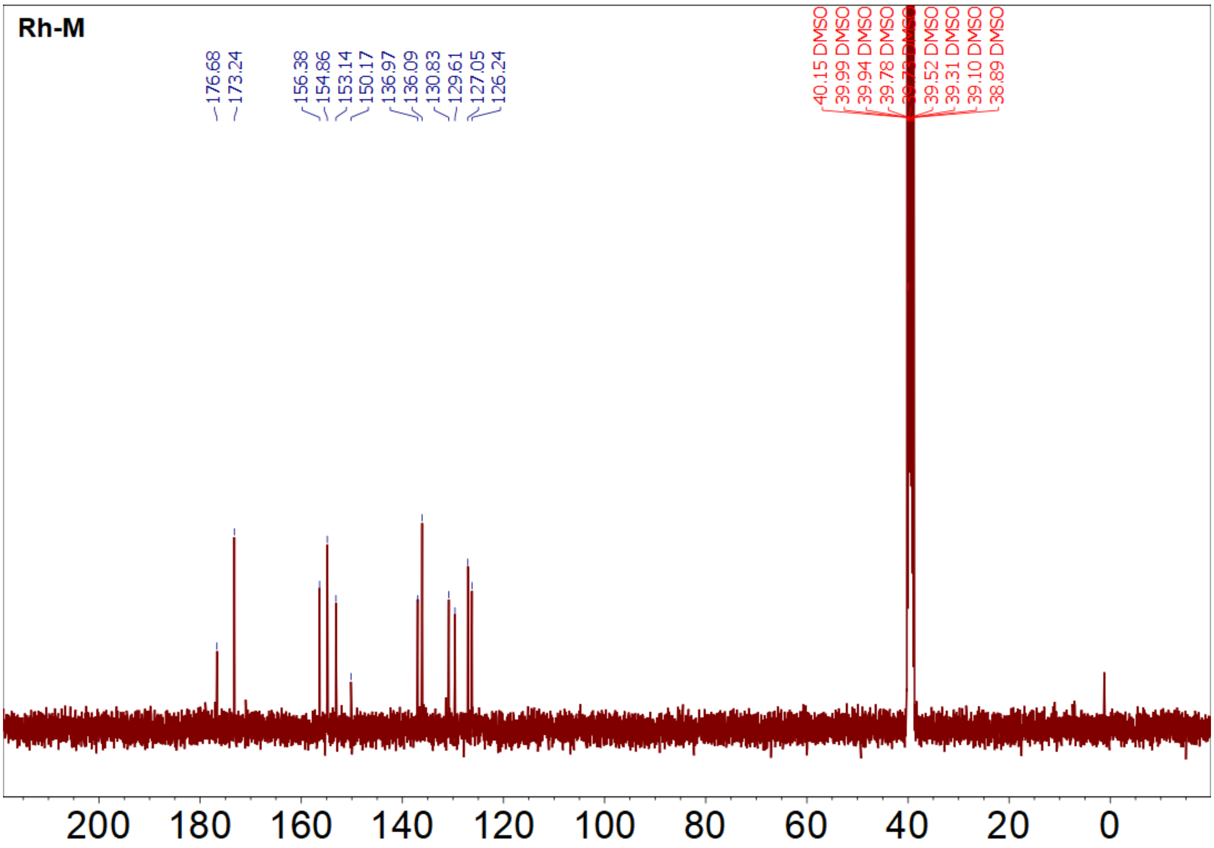

Figure S63 | ^{13}C NMR spectrum of **Rh-M**.

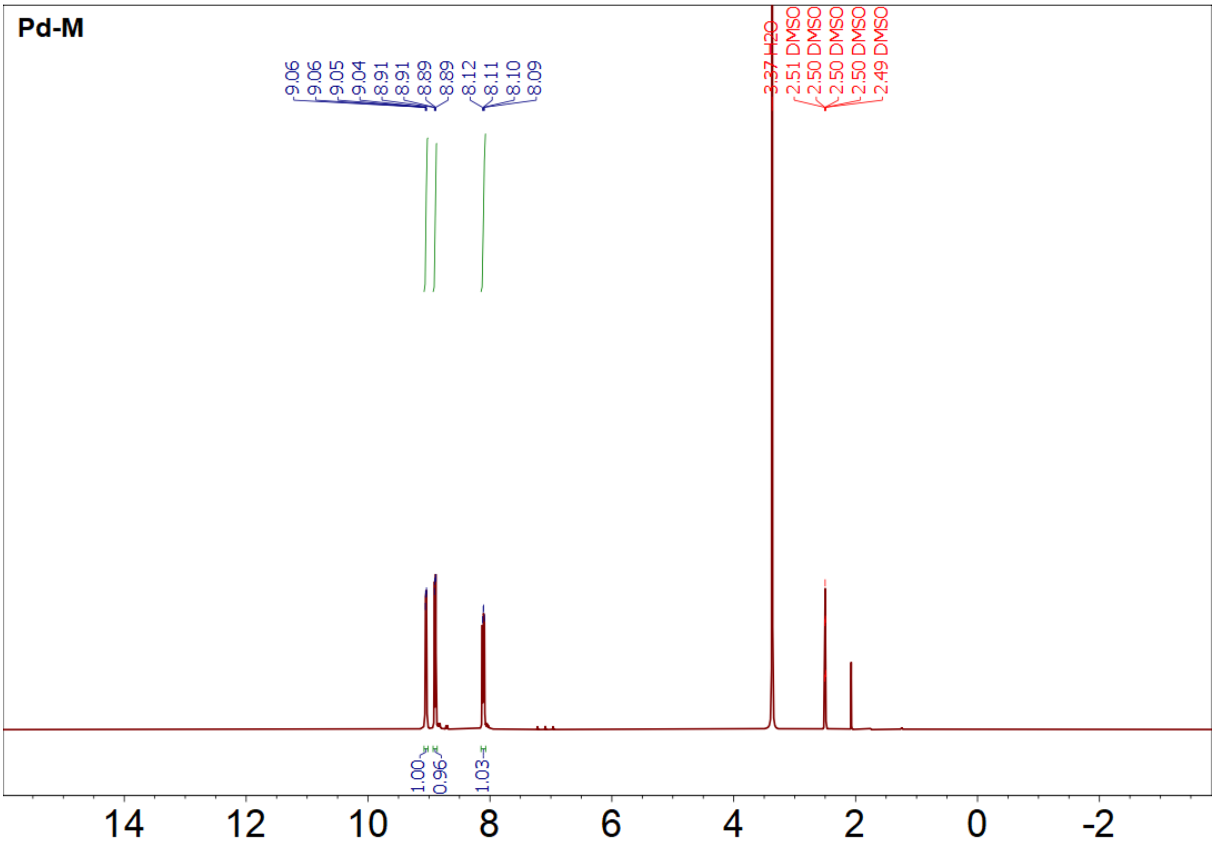

Figure S64 | ^{1}H NMR spectrum of **Pd-M**.

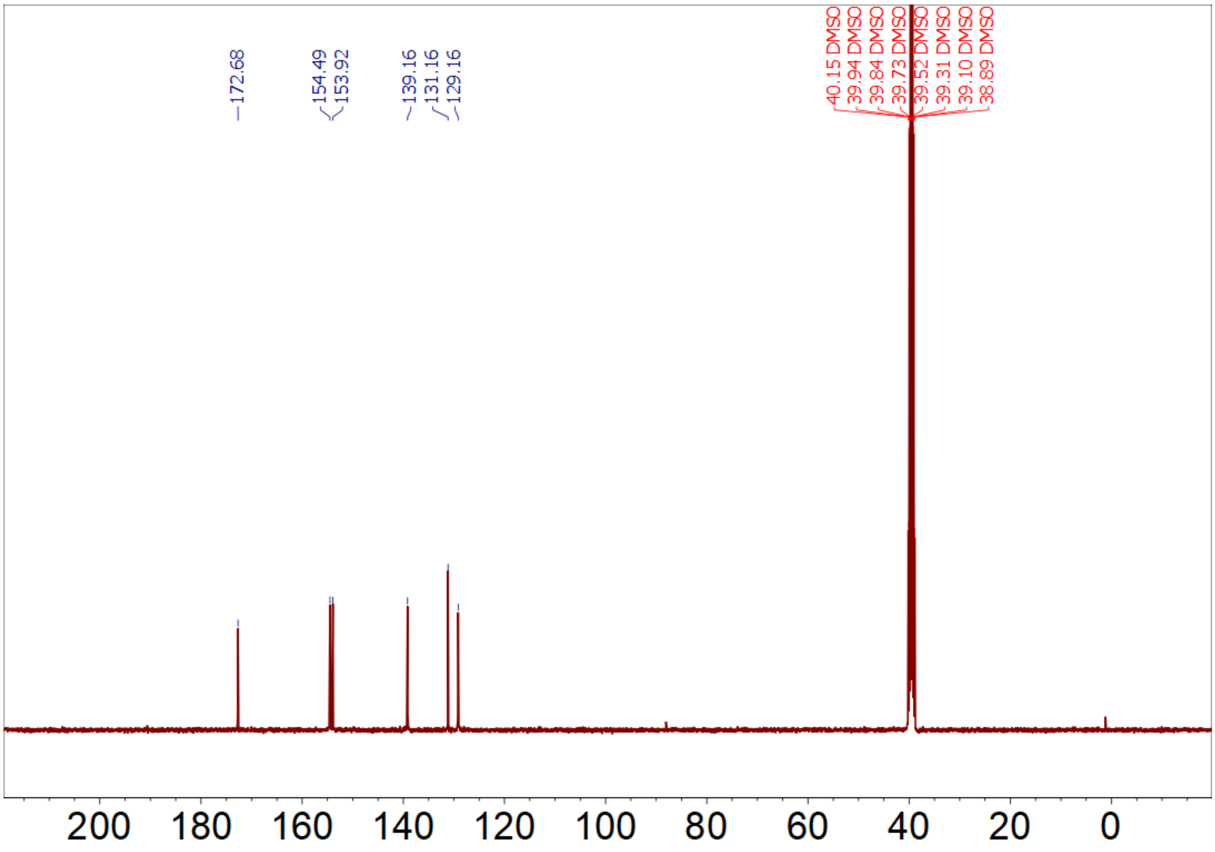

Figure S65 | ^{13}C NMR spectrum of **Pd-M**.

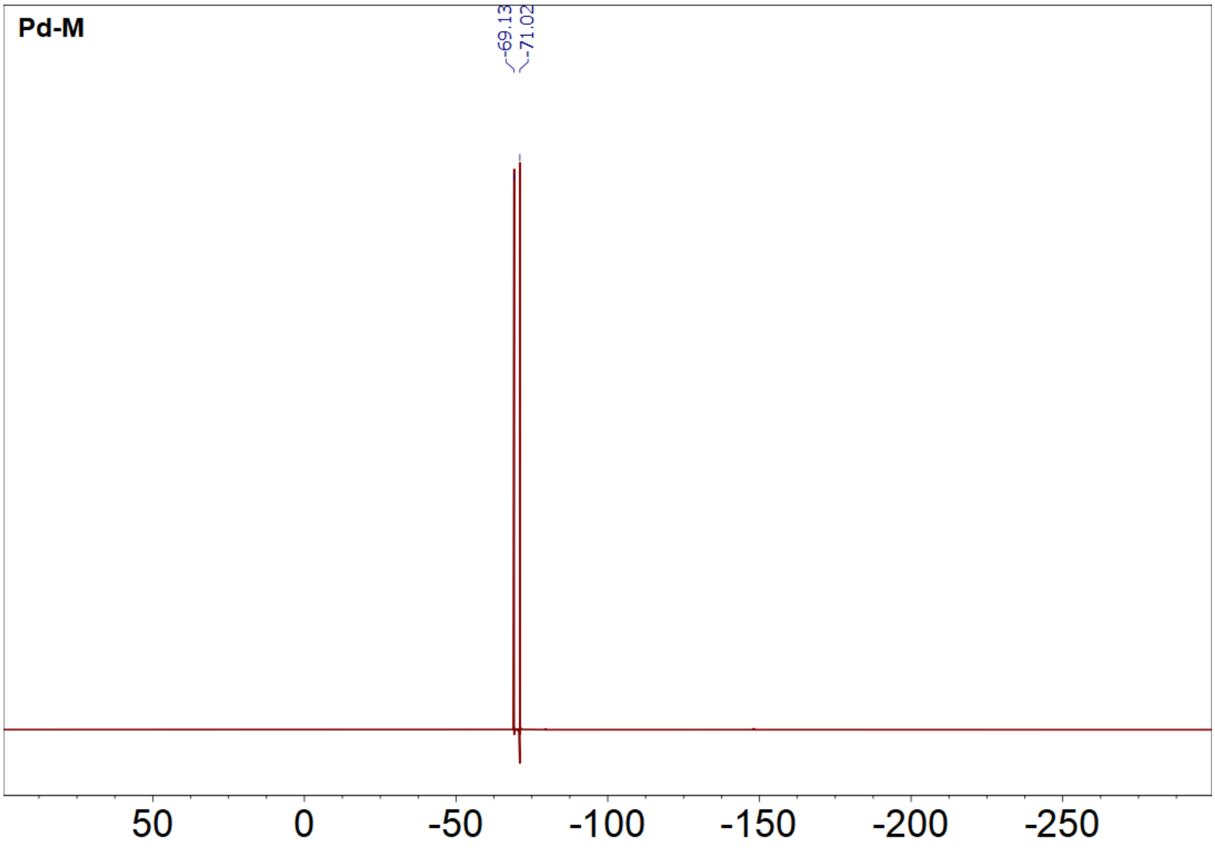

Figure S66 | ^{19}F NMR spectrum of **Pd-M**.

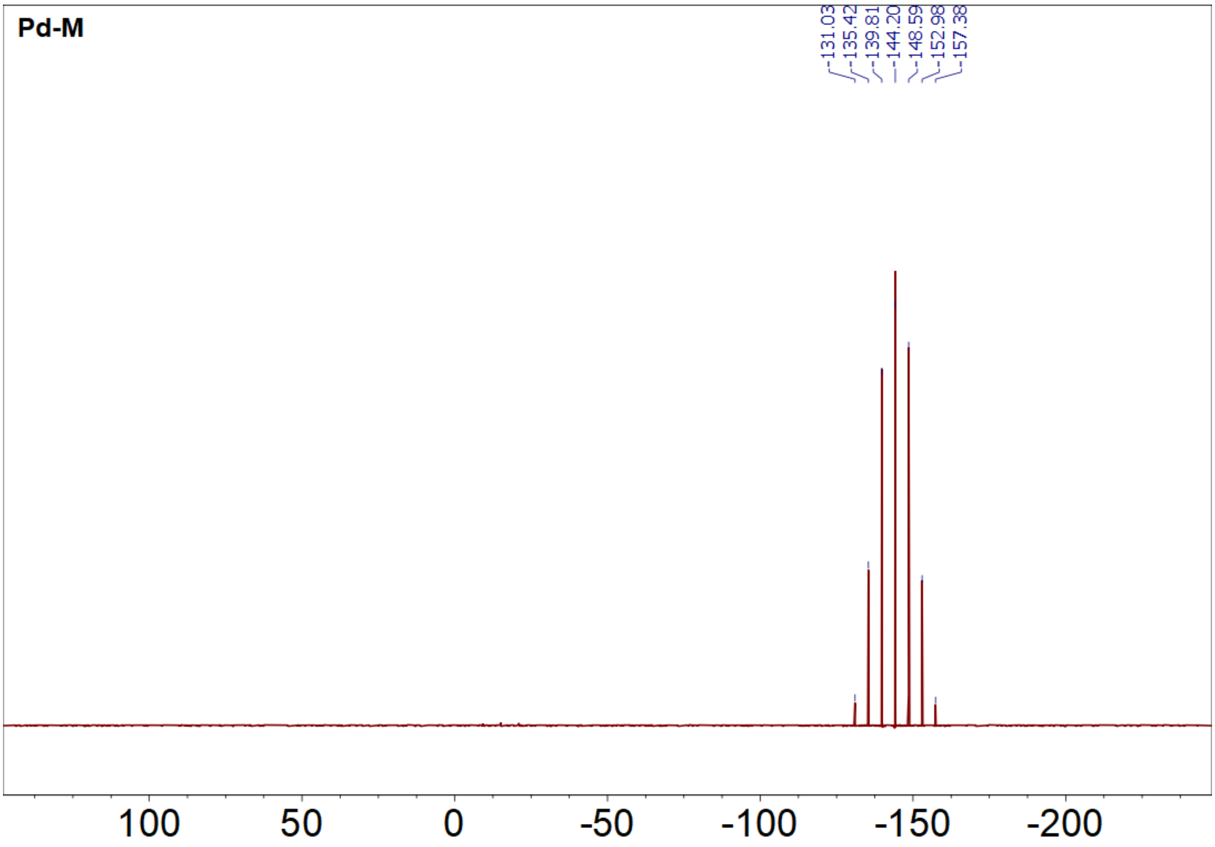

Figure S67 | ^{31}P NMR spectrum of **Pd-M**.

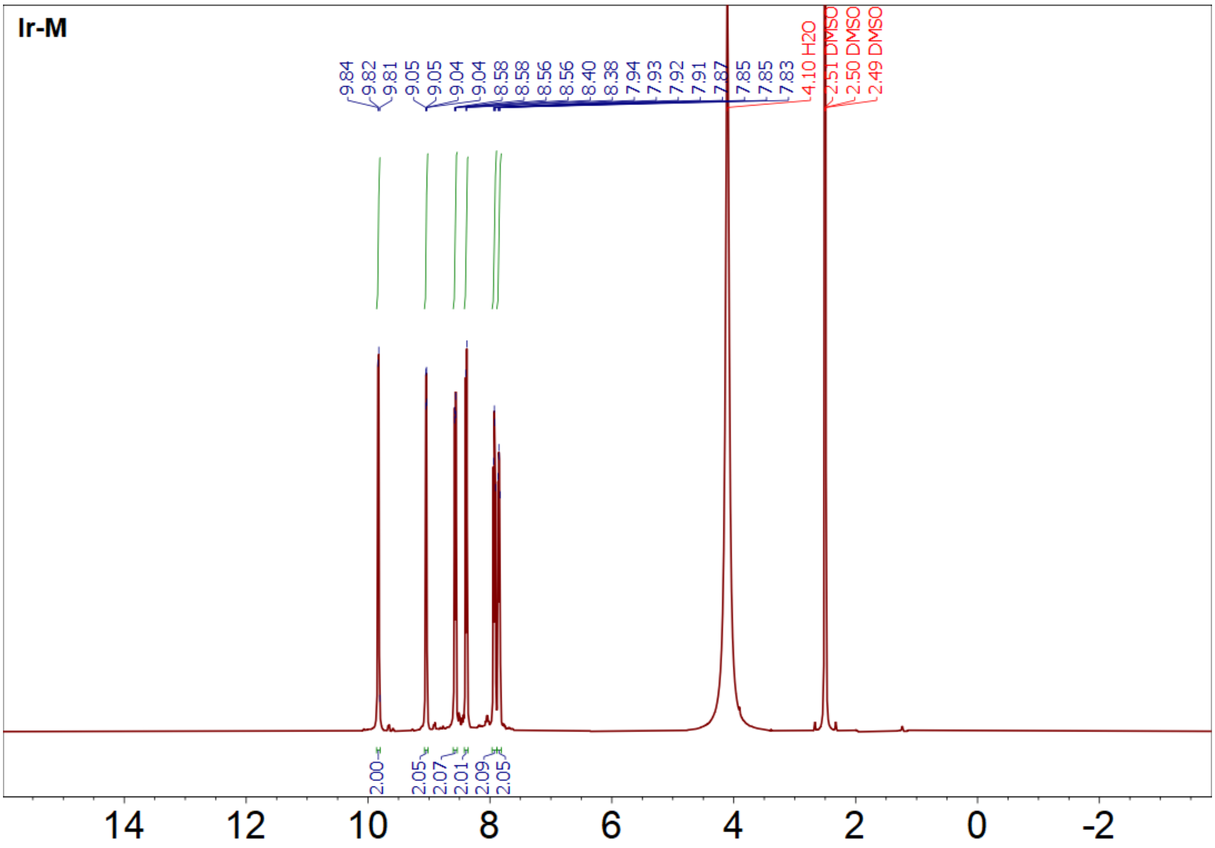

Figure S68 | ^{1}H NMR spectrum of **Ir-M**.

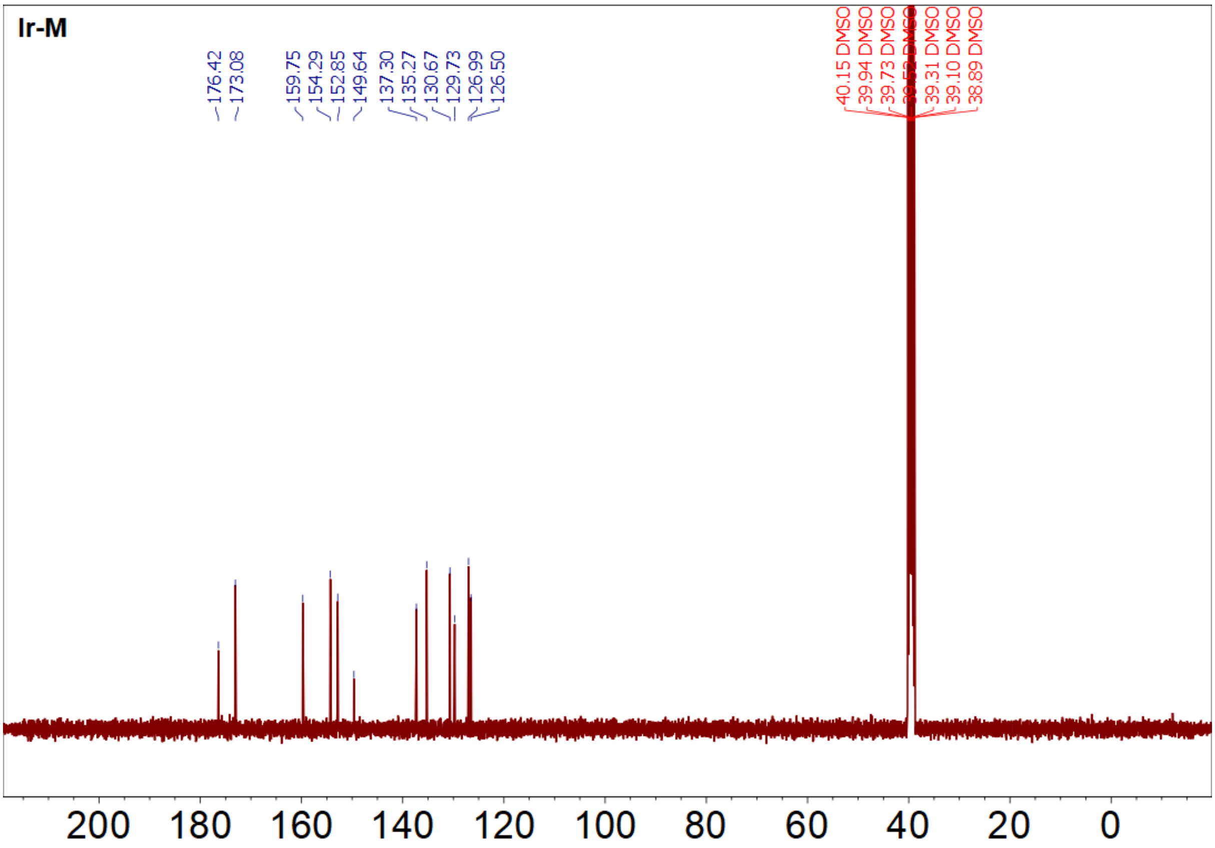

Figure S69 | ^{13}C NMR spectrum of **Ir-M**.

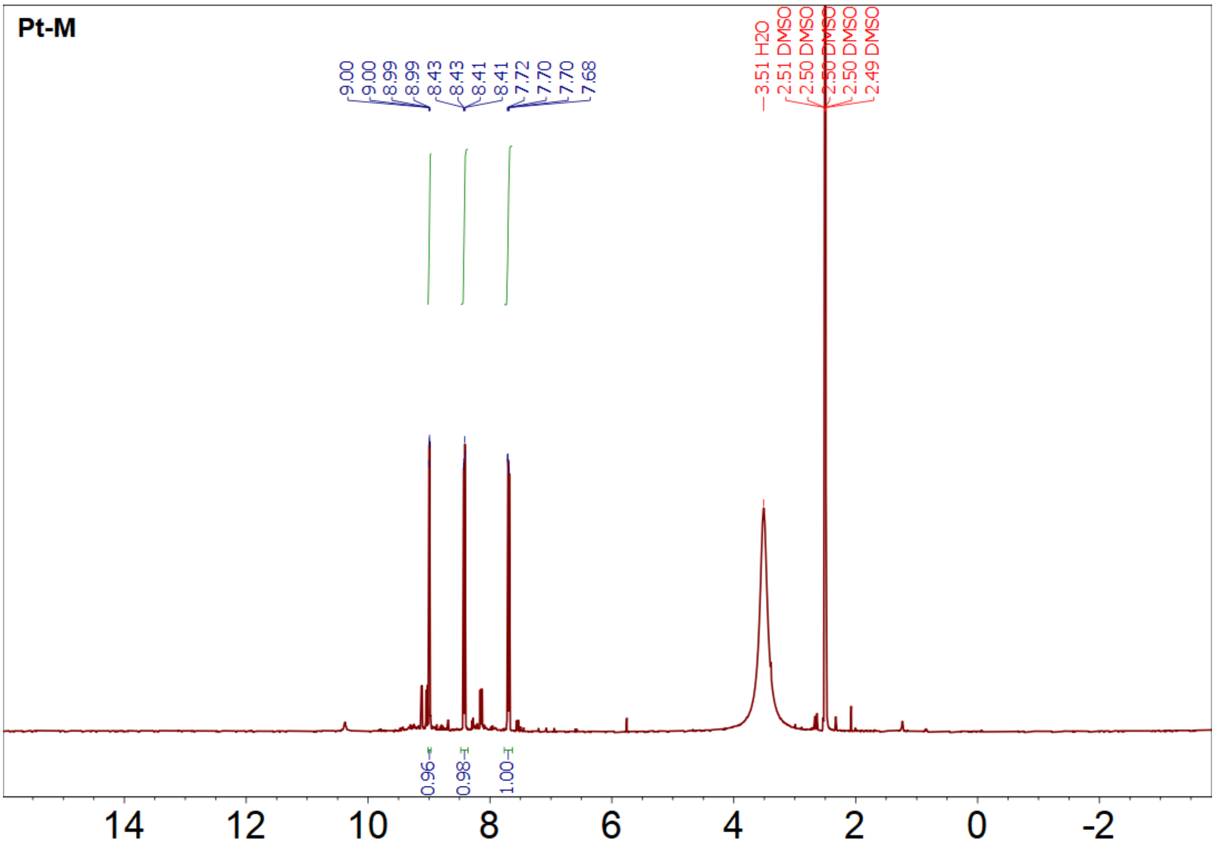

Figure S70 | ^{1}H NMR spectrum of **Pt-M**.

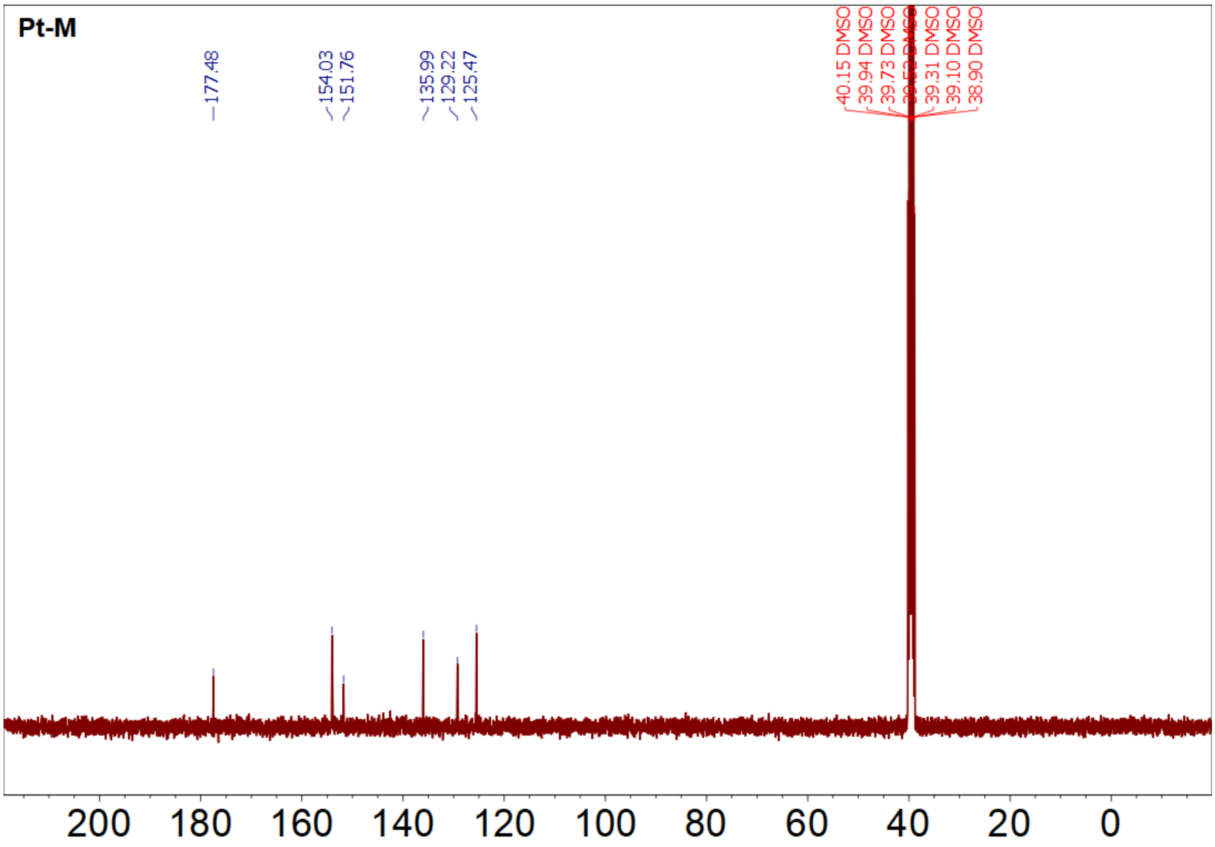

Figure S71 | ^{13}C NMR spectrum of **Pt-M**.

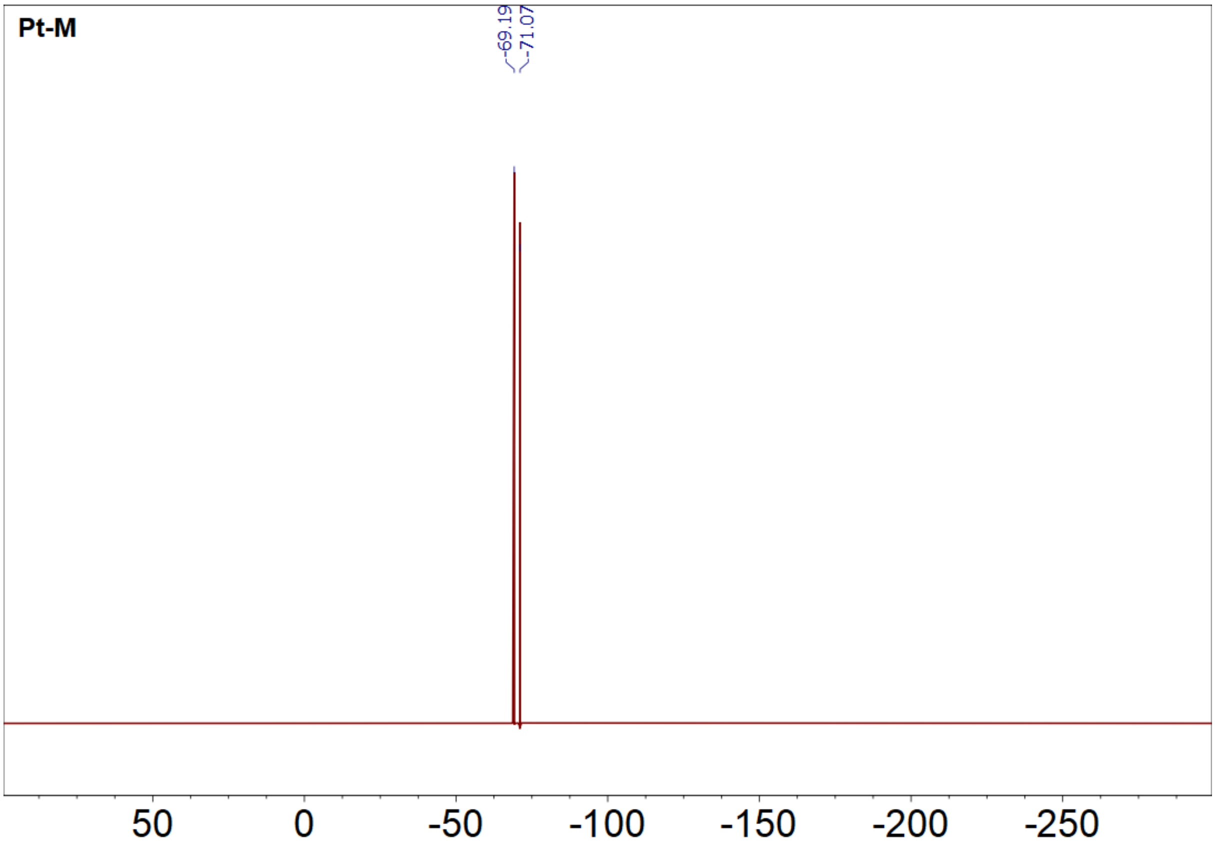

Figure S72 | ^{19}F NMR spectrum of **Pt-M**.

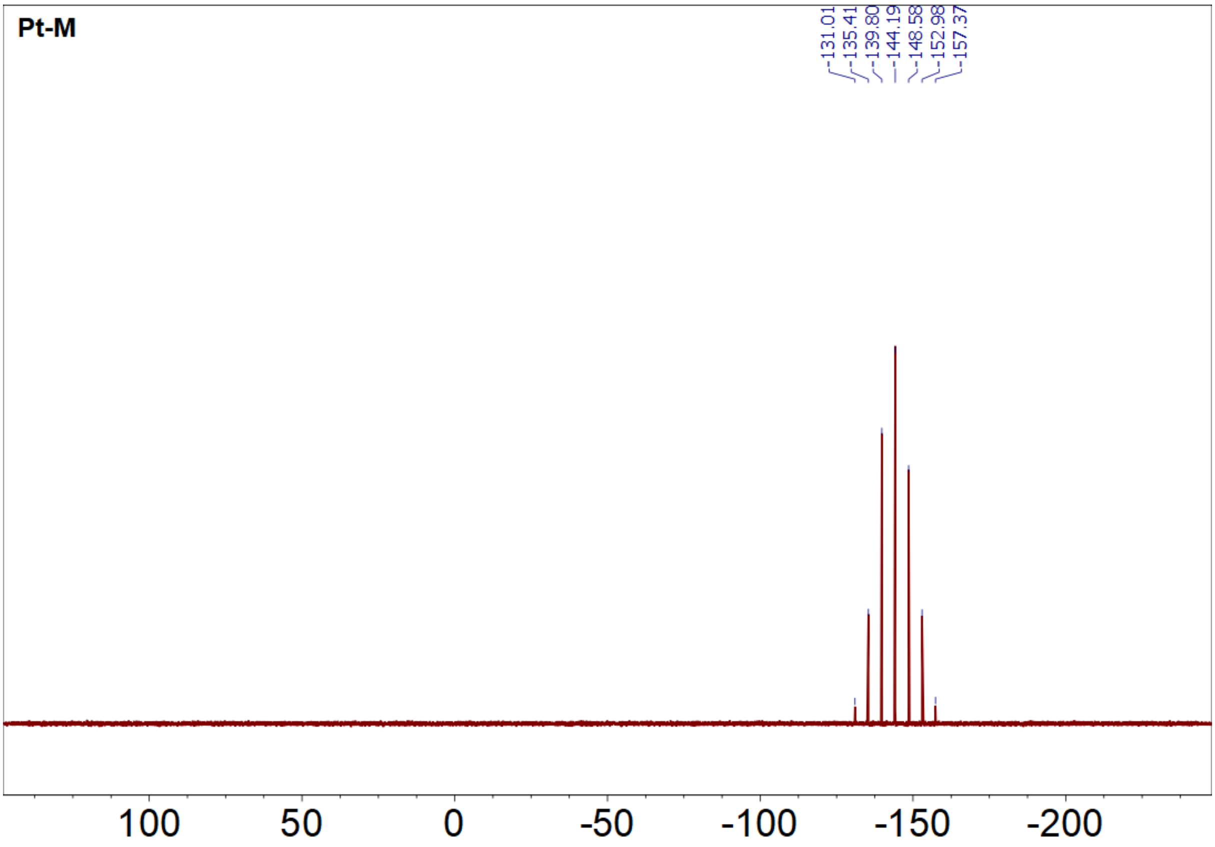

Figure S73 | ^{31}P NMR spectrum of **Pt-M**.

Table S5 | Crystallographic data for **Mn-M**, **Cu-M** and **Zn-M**.

**Compound**	**Mn-M**	**Cu-M**	**Zn-M**
Formula	$C_{24}H_{12}Cl_2MnN_4O_4$	$C_{24}H_{12}Cl_2CuN_4O_4$	$C_{24}H_{12}Cl_2N_4O_4Zn$
$D_{calc.}$/ g cm^{-3}	1.682	1.725	1.735
μ/mm^{-1}	7.630	4.123	4.288
Formula Weight	546.22	554.82	556.65
Color	clear intense orange	clear pale green	clear pale yellow
Shape	irregular	plate	plate
Size/mm^3	0.15×0.09×0.07	0.11×0.08×0.06	0.12×0.07×0.02
T/K	140.00(10)	140.00(10)	140.00(10)
Crystal System	orthorhombic	orthorhombic	orthorhombic
Flack Parameter	-0.008(9)	0.16(4)	-0.03(3)
Hooft Parameter	-0.008(9)	-0.020(8)	-0.03(3)
Space Group	Fdd2	*Fdd*2	*Fdd*2
a/Å	8.2929(3)	8.3070(4)	8.2896(6)
b/Å	41.6277(17)	41.4481(19)	41.175(4)
c/Å	12.4992(6)	12.4103(7)	12.4872(11)
α/°	90	90	90
β/°	90	90	90
γ/°	90	90	90
V/Å^3	4314.9(3)	4273.0(4)	4262.2(6)
Z	8	8	8
Z'	0.5	0.5	0.5
Wavelength/Å	1.54184	1.54184	1.54184
Radiation type	Cu K□	Cu K□	Cu K□
Θ_{min}/°	4.248	4.267	4.295
Θ_{max}/°	75.285	73.694	76.333
Measured Refl's.	7575	7075	10212
Indep't Refl's	2068	1988	2130
Refl's I≥2 σ(I)	1918	1849	1881
R_{int}	0.0282	0.0214	0.0511
Parameters	160	160	160
Restraints	1	1	1
Largest Peak	0.578	0.572	0.772
Deepest Hole	-0.532	-0.372	-0.830
GooF	1.060	1.040	1.089
wR_2 (all data)	0.1255	0.1174	0.1930
wR_2	0.1224	0.1147	0.1838
R_1 (all data)	0.0477	0.0459	0.0739
R_1	0.0442	0.0427	0.0675
**CCDC number**	**2453113**	**2453112**	**2453111**

Supporting references

[1] T. B. Rauchfuss, Ed., in *Inorg. Synth.*, Wiley, **2010**, pp. 148–163.

[2] R. Romeo, L. M. Scolaro, V. Catalano, S. Achar, in *Inorg. Synth.* (Ed.: Marcetta. Y. Darensbourg), Wiley, **1998**, pp. 153–158.

[3] O. B. Peersen, X. L. Wu, I. Kustanovich, S. O. Smith, *J. Magn. Reson. A* **1993**, *104*, 334.

[4] B. M. Fung, A. K. Khitrin, K. Ermolaev, *J. Magn. Reson.* **2000**, *142*, 97.

[5] J. W. M. Osterrieth, J. Rampersad, D. Madden, N. Rampal, L. Skoric, B. Connolly, M. D. Allendorf, V. Stavila, J. L. Snider, R. Ameloot, J. Marreiros, C. Ania, D. Azevedo, E. Vilarrasa-Garcia, B. F. Santos, X. Bu, Z. Chang, H. Bunzen, N. R. Champness, S. L. Griffin, B. Chen, R. Lin, B. Coasne, S. Cohen, J. C. Moreton, Y. J. Colón, L. Chen, R. Clowes, F. Coudert, Y. Cui, B. Hou, D. M. D'Alessandro, P. W. Doheny, M. Dincă, C. Sun, C. Doonan, M. T. Huxley, J. D. Evans, P. Falcaro, R. Ricco, O. Farha, K. B. Idrees, T. Islamoglu, P. Feng, H. Yang, R. S. Forgan, D. Bara, S. Furukawa, E. Sanchez, J. Gascon, S. Telalović, S. K. Ghosh, S. Mukherjee, M. R. Hill, M. M. Sadiq, P. Horcajada, P. Salcedo-Abraira, K. Kaneko, R. Kukobat, J. Kenvin, S. Keskin, S. Kitagawa, K. Otake, R. P. Lively, S. J. A. DeWitt, P. Llewellyn, B. V. Lotsch, S. T. Emmerling, A. M. Pütz, C. Martí-Gastaldo, N. M. Padial, J. García-Martínez, N. Linares, D. Maspoch, J. A. Suárez Del Pino, P. Moghadam, R. Oktavian, R. E. Morris, P. S. Wheatley, J. Navarro, C. Petit, D. Danaci, M. J. Rosseinsky, A. P. Katsoulidis, M. Schröder, X. Han, S. Yang, C. Serre, G. Mouchaham, D. S. Sholl, R. Thyagarajan, D. Siderius, R. Q. Snurr, R. B. Goncalves, S. Telfer, S. J. Lee, V. P. Ting, J. L. Rowlandson, T. Uemura, T. Iiyuka, M. A. Van Der Veen, D. Rega, V. Van Speybroeck, S. M. J. Rogge, A. Lamaire, K. S. Walton, L. W. Bingel, S. Wuttke, J. Andreo, O. Yaghi, B. Zhang, C. T. Yavuz, T. S. Nguyen, F. Zamora, C. Montoro, H. Zhou, A. Kirchon, D. Fairen-Jimenez, *Adv. Mater.* **2022**, *34*, 2201502.

[6] A. R. Blythe, *Polym. Test.* **1984**, *4*, 195.

[7] K. Klementiev, R. Chernikov, *J. Phys. Conf. Ser.* **2016**, *712*, 012008.

[8] B. Ravel, M. Newville, *J. Synchrotron Radiat.* **2005**, *12*, 537.

[9] F. Neese, *WIREs Comput. Mol. Sci.* **2022**, *12*, e1606.

[10] Y. Zhao, D. G. Truhlar, *Theor. Chem. Acc.* **2008**, *120*, 215.

[11] F. Weigend, R. Ahlrichs, *Phys. Chem. Chem. Phys.* **2005**, *7*, 3297.

[12] S. Nehru, J. A. Anitha Priya, S. Hariharan, R. Vijay Solomon, S. Veeralakshmi, *J. Biomol. Struct. Dyn.* **2020**, *38*, 2057.

[13] Z. Mtshali, J. Conradie, *Inorganica Chim. Acta* **2023**, *549*, 121422.

[14] M. D. Pozza, P. Mesdom, A. Abdullrahman, T. D. Prieto Otoya, P. Arnoux, C. Frochot, G. Niogret, B. Saubaméa, P. Burckel, J. P. Hall, M. Hollenstein, C. J. Cardin, G. Gasser, *Inorg. Chem.* **2023**, *62*, 18510.

[15] X. Lu, C. Zhao, *Nat. Commun.* **2015**, *6*, 6616.

[16] J. A. Zamora Zeledón, A. Jackson, M. B. Stevens, G. A. Kamat, T. F. Jaramillo, *J. Electrochem. Soc.* **2022**, *169*, 066505.

[17] Q. Wu, M. Maskus, F. Pariente, F. Tobalina, V. M. Fernández, E. Lorenzo, H. D. Abruña, *Anal. Chem.* **1996**, *68*, 3688.

[18] J. Onuegbu, R. J. Butcher, C. Hosten, U. C. Udeochu, O. Bakare, *Acta Crystallogr. Sect. E Struct. Rep. Online* **2009**, *65*, m1119.

[19] C.-B. Liu, Q.-Q. Wang, H.-Y. Bai, G.-B. Che, Q. Zhang, C.-X. Li, **2013**, DOI 10.5517/CCY6YHK.

[20] C. A. L. Figueiras, J. A. S. Bomfim, R. A. Howie, E. R. T. Tiekink, J. L. Wardell, *Acta Crystallogr. Sect. E Struct. Rep. Online* **2009**, *65*, m1645.
[21] X.-J. Wu, Z.-R. Chen, **2014**, DOI 10.5517/CCRLZ3D.
[22] D. Shen, T. Ren, H. Zhang, M. Chao, C. Sun, P. Gong, S. Zhang, Y.-M. Lee, S. Fukuzumi, W. Nam, *ACS Catal.* **2024**, *14*, 2162.
[23] F. Calderazzo, F. Marchetti, G. Pampaloni, V. Passarelli, *J. Chem. Soc. Dalton Trans.* **1999**, 4389.
[24] K. Choroba, B. Filipe, A. Świtlicka, M. Penkala, B. Machura, A. Bieńko, S. Cordeiro, P. V. Baptista, A. R. Fernandes, *J. Med. Chem.* **2023**, *66*, 8580.
[25] J. R. Hickson, S. J. Horsewill, C. Bamforth, J. McGuire, C. Wilson, S. Sproules, J. H. Farnaby, *Dalton Trans.* **2018**, *47*, 10692.
[26] A. M. Al-Ajlouni, A. Günyar, M. Zhou, P. N. W. Baxter, F. E. Kühn, *Eur. J. Inorg. Chem.* **2009**, *2009*, 1019.
[27] R. B. De Alencastro, J. A. S. Bomfim, C. A. L. Filgueiras, R. A. Howie, J. L. Wardell, *Appl. Organomet. Chem.* **2005**, *19*, 479.
[28] H. Braband, S. Imstepf, M. Felber, B. Spingler, R. Alberto, *Inorg. Chem.* **2010**, *49*, 1283.
[29] Z. Xie, X. Liu, A. J. S. Valentine, V. M. Lynch, D. M. Tiede, X. Li, K. L. Mulfort, *Angew. Chem. Int. Ed.* **2022**, *61*, e202111764.
[30] X. Li, J. Waser, *J. Am. Chem. Soc.* **2024**, *146*, 29712.
[31] H.-H. Li, M. Wang, S.-W. Huang, J.-B. Liu, X. Lin, Z.-R. Chen, *Synth. React. Inorg. Met.-Org. Nano-Met. Chem.* **2011**, *41*, 1351.
[32] L. Bertolo, S. Tamburini, P. A. Vigato, W. Porzio, G. Macchi, F. Meinardi, *Eur. J. Inorg. Chem.* **2006**, *2006*, 2370.
[33] O. Galangau, J. Flores Gonzalez, V. Montigaud, V. Dorcet, B. Le Guennic, O. Cador, F. Pointillart, *Magnetochemistry* **2020**, *6*, 19.
[34] J. Krzystek, J.-H. Park, M. W. Meisel, M. A. Hitchman, H. Stratemeier, L.-C. Brunel, J. Telser, *Inorg. Chem.* **2002**, *41*, 4478.
[35] G. Novitchi, S. Jiang, S. Shova, F. Rida, I. Hlavička, M. Orlita, W. Wernsdorfer, R. Hamze, C. Martins, N. Suaud, N. Guihéry, A.-L. Barra, C. Train, *Inorg. Chem.* **2017**, *56*, 14809.
[36] E. Ya. Misochko, A. V. Akimov, D. V. Korchagin, J. Nehrkorn, M. Ozerov, A. V. Palii, J. M. Clemente-Juan, S. M. Aldoshin, *Inorg. Chem.* **2019**, *58*, 16434.
[37] L. Escriche-Tur, M. Font-Bardia, B. Albela, M. Corbella, *Dalton Trans.* **2017**, *46*, 2699.
[38] L. Liu, X. Wu, L. Wang, X. Xu, L. Gan, Z. Si, J. Li, Q. Zhang, Y. Liu, Y. Zhao, R. Ran, X. Wu, D. Weng, F. Kang, *Commun. Chem.* **2019**, *2*, 18.
[39] B. Rusinque, S. Escobedo, H. De Lasa, *Catalysts* **2021**, *11*, 405.
[40] Y. Bai, C. Li, L. Liu, Y. Yamaguchi, M. Bahri, H. Yang, A. Gardner, M. A. Zwijnenburg, N. D. Browning, A. J. Cowan, A. Kudo, A. I. Cooper, R. S. Sprick, *Angew. Chem. Int. Ed.* **2022**, *61*, e202201299.
[41] W. Liu, L. Cao, W. Cheng, Y. Cao, X. Liu, W. Zhang, X. Mou, L. Jin, X. Zheng, W. Che, Q. Liu, T. Yao, S. Wei, *Angew. Chem. Int. Ed.* **2017**, *56*, 9312.
[42] H. Su, M. Liu, W. Cheng, X. Zhao, F. Hu, Q. Liu, *J. Mater. Chem. A* **2019**, *7*, 11170.
[43] Y. Li, Y. Wang, C.-L. Dong, Y.-C. Huang, J. Chen, Z. Zhang, F. Meng, Q. Zhang, Y. Huangfu, D. Zhao, L. Gu, S. Shen, *Chem. Sci.* **2021**, *12*, 3633.
